# A machine learning perspective on three decades of methanol synthesis: research framework and experimental operation insights

**DOI:** 10.1038/s44172-026-00706-4

**Published:** 2026-06-10

**Authors:** Mo Yan, Chengxi Yao, Shihao Wu, Muhammad Asif, Nuning Anugrah Putri Namari, Joohwan Ha, Ziyang Wang, Jaewon Lee, Dayoung Yu, Seokchan Lee, Seungjae Lee, Hao Yu, Xiaoming Duan, Taesung Kim

**Affiliations:** 1 Institute for Carbon-Neutral Energy Research (I²CNER), Kyushu University, Fukuoka, Japan; 2 School of Mechanical Engineering, Sungkyunkwan University, Suwon, Gyeong Gi-Do South Korea; 3 Institute for Advanced Ceramics, School of Materials Science and Engineering, Harbin Institute of Technology, Harbin, China; 4 Department of Environmental Sciences, COMSATS University, Islamabad Abbottabad Campus, Islamabad, Pakistan; 5 Department of Semiconductor Convergence Engineering, Sungkyunkwan University, Suwon, Gyeong Gi-Do South Korea; 6 SKKU Advanced Institute of Nanotechnology, Sungkyunkwan University, Suwon, Gyeong Gi-Do South Korea; 7 School of Engineering, The University of Edinburgh, Edinburgh, UK

**Keywords:** Chemical engineering, Scientific data, Chemical engineering, Heterogeneous catalysis

## Abstract

Heterogeneous catalysis optimization relies on the coordinated selection of catalyst composition, synthesis procedure, and reaction conditions, yet the empirical links between these variables and catalytic performance remain scattered across individual studies. Here, we show that an interpretable machine learning (ML) framework can decode this complexity by integrating disparate literature data. We curate, to our knowledge, the first unified dataset from 200 papers spanning three decades of CO_2_-to-methanol synthesis over Cu/ZnO-based catalysts, and derive a subset of apparent activation energy (*E*_app_). Using the dataset, we optimize six ML models via Bayesian hyperparameter tuning and apply regularization strategies to mitigate overfitting. We then use SHAP to quantify the non-linear impact of catalyst properties, synthesis procedures, and reaction conditions on methanol selectivity, CO_2_ conversion, and methanol space-time yield. Crucially, this data-driven approach uncovers previously obscured operating windows for achieving high methanol productivity at milder pressures than conventionally assumed. Extending the ML framework to the *E*_app_ kinetic subset further identifies regime-sensitive signatures. A controlled experiment over commercial catalysts corroborates the positive space velocity–*E*_app_ relationship inferred from ML models. These results show how interpretable ML built on curated legacy experiments can support operating-window selection, hypothesis-driven kinetic experiments, and practical optimization of mature catalytic technologies.

## Abstract (web summary)

Optimization of heterogeneous catalytic processes requires balancing catalyst formulation and reaction conditions. Mo Yan, Chengxi Yao and colleagues report a curated three-decade dataset of CO_2_-to-methanol and an interpretable machine learning framework to map nonlinear performance trends.

## Introduction

Catalytic performance arises from the complex interplay of interdependent variables, such as substrates, catalyst composition, temperature, pressure, and so on, the collective optimization of which remains a persistent challenge^1,2^. In a typical laboratory, particularly in resource-limited environments, discovery of optimal reaction parameters often begins with a small subset of these conditions due to time and cost limitations^3,4^. Although automated synthesis platforms have significantly accelerated reaction optimization^5–7^, exhaustive examination of high-dimensional reaction conditions remains analytically difficult and experimentally expensive to carry out. Traditionally, chemists navigate this complexity by drawing from literature on analogous reactions and leveraging their accumulated mechanistic insights and experience^3,8,9^. However, the interactions among variables influencing multiple performance metrics are too intricate, and there is no universally accepted figure of merit^1^. Industrial-scale catalytic processes exemplify these optimization challenges, where practical improvement often requires balancing conversion, selectivity, productivity, catalyst durability, hydrogen utilization, and pressure-related process penalties rather than optimizing isolated parameters^3,10,11^.

In recent years, there has been a growing appreciation that machine learning (ML) methods are having a transformative impact on predicting catalytic behavior and guiding the rational design of catalytic systems^12–16^. Moreover, by using interpretable models or post-hoc explanation techniques (for example, SHapley Additive exPlanations, SHAP), one can extract human-understandable knowledge from ML models, turning them into tools for scientific discovery rather than just prediction^17–19^.

In this regard, combining literature-extracted data with ML bears great potential as a decision-support layer that prioritizes promising operating windows and catalyst formulations before time-intensive validation experiments^20–22^. This potential is particularly relevant for mature, data-rich catalyst families, where decades of accumulated studies can serve as an engineering resource rather than merely historical background^23^. Despite this potential, purely data-driven ML models often suffer from data quality issues, including biases and a lack of negative examples^14^. Additionally, many current studies draw heavily from computational data (for example, density functional theory, DFT)^24,25^ and open databases (for example, Materials Project^26^ and Open Quantum Materials Database^27^), often overlooking critical experimental conditions and operational details^28–30^. This incompatibility represents a fundamental impasse to ML for practical ─ and therefore the understanding of underpinning data-driven reaction optimization ─ for engineering researchers.

Here, we develop a conceptually simple yet practically effective framework, utilizing archival experimental data and off-the-shelf ML tools to systematically guide condition optimization in heterogeneous catalytic studies. Given that sustainable methanol synthesis from CO_2_ and green hydrogen (CO_2_ + 3H_2_ → CH_3_OH + H_2_O) is promising for carbon neutrality and has a long history^31,32^, we selected CO_2_ hydrogenation to methanol as a model system to demonstrate the workflow. Although ML has already been applied to CO_2_-to-methanol catalysis, existing studies address substantially different problem definitions and data regimes. Some works rely on DFT-assisted or adsorption-energy-based screening to identify hypothetical alloy catalysts^33^. Other literature-based studies predict single or limited performance targets across broader catalyst families, such as methanol space-time yield or conversion and selectivity metrics^34,35^. More recent process-oriented studies further extend ML toward optimization, techno-economic analysis, and life-cycle assessment, while closed-loop Bayesian-optimization studies accelerate catalyst discovery through newly generated high-throughput experimental libraries^36^. These advances are important, yet they do not provide an experimentally grounded, Cu/ZnO-centered (the catalyst family with the deepest historical foundation and the most established benchmark status in methanol synthesis) framework that simultaneously resolves catalyst composition, synthesis history, operating conditions, multiple catalytic outputs, and regime-sensitive apparent kinetics within one benchmark catalyst family. A detailed comparison with representative prior ML studies is provided in Table S1.

Specifically, we constructed a new hand-curated dataset that systematically compiles three decades (1995–2025) of experimental studies on CO_2_ hydrogenation to methanol over Cu/ZnO-based catalysts. To the best of our knowledge, this represents the first comprehensive, human-curated dataset in this domain, encompassing 2476 data points detailing catalyst composition and preparation methods, reaction conditions, and performance indicators for methanol synthesis over Cu/ZnO-based catalysts. We trained six common ML algorithms via Bayesian hyperparameter tuning and applied regularization strategies to mitigate overfitting based on this curated dataset to predict methanol selectivity, CO selectivity, CO_2_ conversion, and space-time yield. The best-performing model underwent detailed interpretation via SHAP analysis to identify critical parameters and elucidate their impact on catalytic outcomes. Additionally, leveraging the trained model, we virtually screened operational conditions and catalyst compositions to identify scenarios promising superior methanol yields. Given the mechanistic complexity of CO_2_ hydrogenation, we extracted apparent activation energies (*E*_app_) from temperature-dependent data using the Arrhenius equation and, to our knowledge, established the first literature-based kinetic dataset for this system. We then performed our ML framework to  the *E*_app_ analysis. A targeted fixed-bed validation experiment independently confirms the positive relationship between GHSV and the observed methanol-based *E*_app_ derived from ML. Additionally, an auxiliary CO_2_ electroreduction case study supports transferability across catalytic regimes when sufficiently structured data are available. Our study highlights how accessible machine learning, coupled with human-curated literature data, can shift the paradigm of reaction optimization from resource-intensive trial-and-error to data-driven discovery.

## Methods

### Curating literature data into a machine-readable dataset

Machine learning models rely critically on the availability of structured, high-quality data. While in many domains such data are generated via automated experimentation or high-throughput pipelines^3,4^, in legacy catalysis such as CO_2_ hydrogenation, the relevant data are predominantly embedded in decades of dispersed literature. Cu/ZnO-based catalysts have long served as a benchmark catalyst family for CO_2_ hydrogenation to methanol, originally developed for syngas (CO and H_2_) conversion in the 1960s^37–39^. However, individual studies typically focus on a small slice of this multidimensional space, resulting in fragmented or even conflicting observations. Harnessing the collective knowledge embedded in decades of literature data is therefore an appealing strategy to discern the overarching trends^22,40^. As such, we manually curated a dataset for Cu/ZnO-catalyzed CO_2_ hydrogenation to methanol spanning over thirty years of experimental reports. Importantly, this curation was not merely clerical, as key experimental quantities were often scattered across figures, supplementary tables, and source-specific reporting formats, requiring expert judgment for unit harmonization, output reconstruction, and consistency screening before model training.

**Fig. 1:**
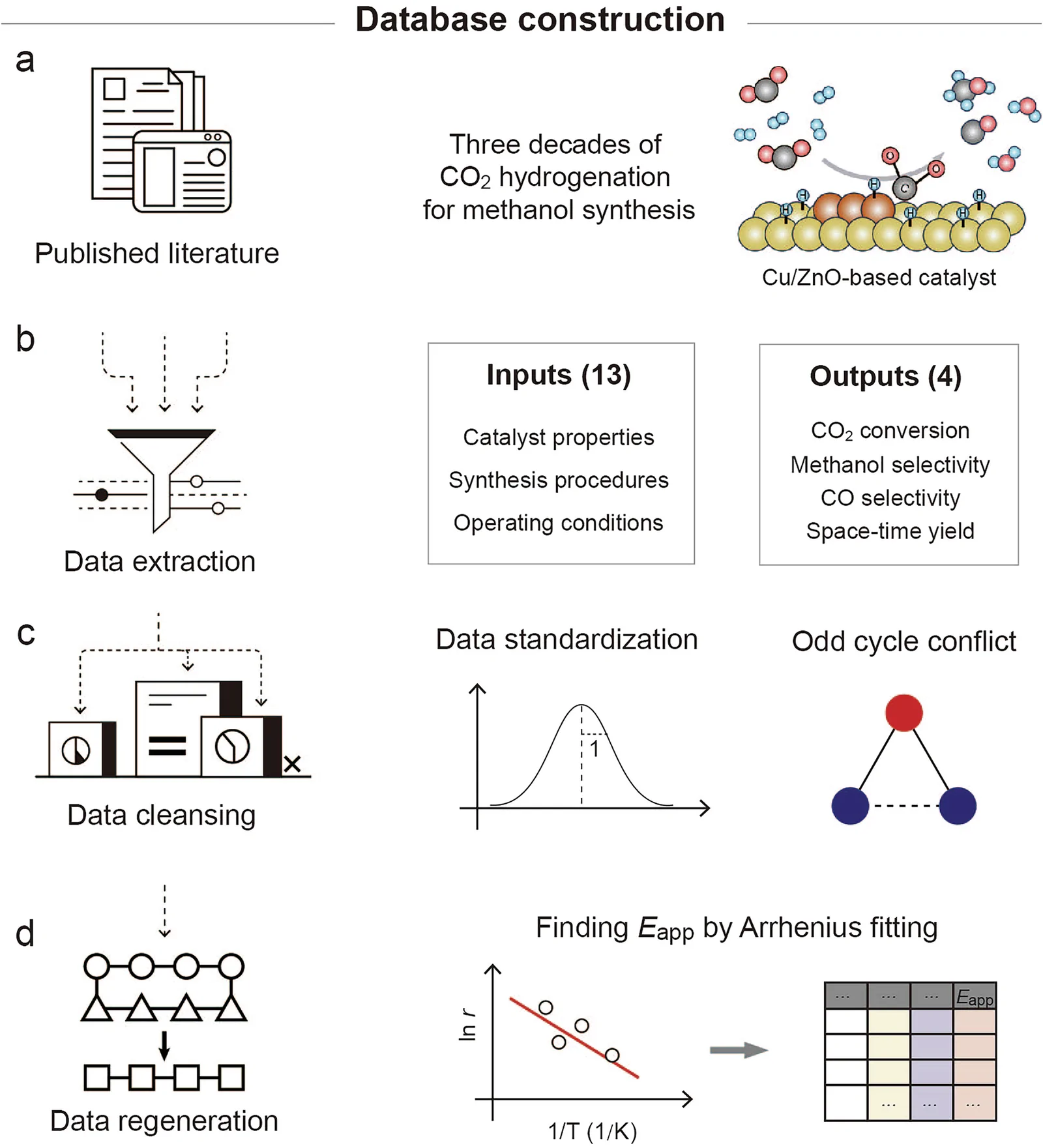
Workflow for constructing the Cu/ZnO-catalyzed CO_2_-to-methanol database for downstream interpretable ML analysis. **a** Three decades of published studies were systematically surveyed for experiments on CO_2_ hydrogenation over Cu/ZnO-based catalysts. **b** Manual extraction captured 13 input descriptors (catalyst composition, synthesis procedures and operating conditions) and four performance outputs (CO_2_ conversion, methanol selectivity, CO selectivity and space-time yield). **c** Entries were unit-standardized, normalized and screened for internal consistency, resolving odd-cycle conflicts and other anomalies. **d** Where temperature-dependent rate data were available, apparent activation energies (*E*_app_) were regenerated via Arrhenius equation fits, completing a machine-readable kinetic dataset.

**Fig. 2:**
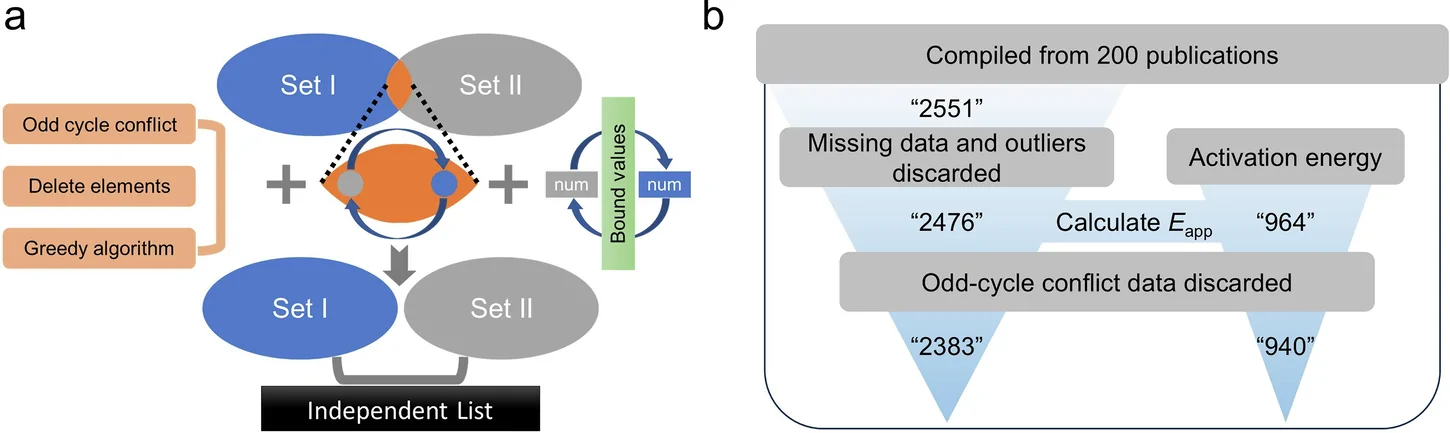
Data curation and symmetry conflict resolution workflow. **a** Schematic of the greedy algorithm used to identify and resolve odd-cycle conflicts arising from unordered promoter pairs. By detecting minimal sets of conflicting entries and removing redundancies, the dataset is reduced to a permutation-invariant, independent list suitable for modeling. **b** Dataset reduction flowchart. Of the 2551 initially extracted entries from 200 publications, 2476 remained after excluding records with non-reconstructable performance outputs and non-comparable or erroneous outlier reports (Suppl. Note 3). The remaining descriptor-level missingness was handled by imputation (Suppl. Note 3). A final refinement step, removing odd-cycle conflicts, yielded 2383 valid data points used for model training. A similar process was applied to the activation-energy subset.

Our data acquisition protocol began with a keyword-based search of the Web of Science and Scopus databases for literature published between 1995 and 2025 (Fig. 1a). The query ([“CO_2_” AND “methanol” AND “Cu” AND “Zn”]) yielded an initial corpus of 803 articles. Following a rigorous manual screening of these works, 200 publications were identified as containing suitable quantitative data for extraction on CO_2_ hydrogenation to methanol. From these, we compiled a dataset where each entry corresponds to a unique experimental result. As shown in Fig. 1b, the data were structured into two primary classes: input parameters and output performance metrics. The inputs were grouped into three categories: (i) catalyst properties, (ii) synthesis methods, and (iii) reaction conditions. The primary outputs quantifying catalytic performance were CO_2_ conversion (X_CO2_, %), methanol selectivity (S_MeOH_, %), CO selectivity (S_CO_, %), and methanol space-time yield (STY_MeOH_, g_MeOH_ kg_cat_^−1^ h^−1^). The full list of input and output features is summarized in Table 1. To ensure consistency across sources, all reported quantities were converted to standardized units prior to modeling. A detailed summary of unit conversions and normalization procedures is provided in Table S2 (Supplementary Note 2). For the ML models, replacing °C by K (or vice versa) corresponds to an affine transformation and, after feature standardization, does not alter the information content of the temperature descriptor. Absolute temperature in K was used wherever physically required, namely in the Arrhenius-based *E*_app_ analysis in a later section. For data not explicitly reported in tables or text, numerical values were extracted from plots using the open-source WebPlotDigitizer tool^41^. All data were transcribed impartially as reported in the source publication to ensure the integrity of the final dataset. Some studies reported only partial information, resulting in missing values in either (i) performance outputs (e.g., STY_MeOH_ or X_CO2_ not explicitly reported) or (ii) selected input descriptors (e.g., catalyst surface area or calcination time). For performance outputs, we reconstructed missing STY_MeOH_ or X_CO2_ whenever the complementary metrics were available using Eqs. (S1) and (S2) (Supplementary Note 2), while performance entries lacking sufficient information to reconstruct the key performance outputs were excluded (Supplementary Note 2), leaving 2476 entries from the 2551 extracted records (Fig. 2b). For the remaining dataset, sparse descriptor-level missingness was handled by kernel density estimation (KDE)-based stochastic imputation for continuous variables and empirical distribution sampling for categorical variables, thereby preserving the full entry count (Supplementary Note 3)^42^.

**Table 1:** Input features and output performance metrics used for ML modeling of CO_2_ hydrogenation to methanol over Cu/ZnO-based catalysts

Category	Features	Units
Catalyst properties	Molar ratio (Zn:Cu)	--
	Promoter 1	--
	Molar ratio (Promoter 1:Cu)	--
	Promoter 2	--
	Molar ratio (Promoter 2:Cu)	--
	Catalyst surface area	m^2^ g^−1^
Synthesis methods	Type of synthesis procedure	--
	Calcination temperature	°C
	Calcination time	h
Reaction conditions	Temperature	°C
	Pressure	bar
	Ratio of H_2_:CO_2_	--
	GHSV	cm^3^ g_cat_^−1^ h^−1^
Performance metrics	CO_2_ conversion efficiency (X_CO2_)	%
	Methanol selectivity (S_MeOH_)	%
	CO selectivity (S_CO_)	%
	Methanol space-time yield (STY_MeOH_)	g_MeOH_ kg_cat_^−1^ h^−1^

### Data cleansing and pre-processing

Data cleansing and pre-processing are essential for efficiently applying ML^34,43^. Prior to model training, the curated dataset underwent several targeted pre-processing steps to enhance reliability and robustness (Fig. 1c). Promoter identities were represented by Magpie-derived (Magpie, a composition-based feature engineering framework, well suited to small-to-medium materials datasets that require interpretable descriptors for conventional ML) chemistry-informed descriptors^44^, whereas the type of synthesis procedure was retained as a one-hot-encoded process category^13^. Several numerical features showed highly skewed distributions, which can bias gradient-based learning algorithms. After the initial screening (removal of non-reconstructable records) and the KDE-based imputation of the remaining descriptor missingness, these features were first transformed using a natural logarithm to produce a more Gaussian-like distribution. Subsequently, all numerical features were standardized to zero mean and unit variance (Z-score normalization) to help the ML model converge faster and improve performance (Fig. S1)^45^.

The final and most critical data pre-processing challenge arises from permutation-invariant features, particularly unordered promoter combinations. For instance, a catalyst promoted with both Ga and Pd is chemically identical regardless of the reporting order (e.g., Cu/Zn–Ga–Pd vs. Cu/Zn–Pd–Ga), assuming their roles are symmetric. Naively treating these features as ordered based on their position in a data table creates artificial distinctions between chemically equivalent catalysts^23,46,47^. This violation of physical symmetry leads to data duplication, which increases the risk of overfitting and inflates model error during extrapolation. To enforce this symmetry in our model, we implemented a greedy algorithm to establish a canonical representation for all unordered feature sets (Fig. 2a). This algorithm resolves simple symmetrical redundancies and is designed to handle more complex odd cycle conflicts that can arise with three or more promoters, all while minimizing the loss of valuable data points^48^. This final refinement yielded the 2383 unique, permutation-invariant data points of catalytic performance metrics used for model training, enhancing generalizability and interpretability (Fig. 2b).

### Construction of the kinetic subset

In addition to performance metrics, elucidating reaction mechanisms is a central yet challenging goal in catalysis^17^. To generate mechanistic insights, we constructed a dedicated kinetic dataset comprising apparent activation energies (*E*_app_) for CO_2_ hydrogenation to methanol (Fig. 1d). These values were obtained via two routes: a small fraction was directly extracted from publications that reported activation energies, while the majority was derived by fitting temperature-dependent reaction rate data from our primary dataset to the Arrhenius equation (details of the fitting procedure and filtering thresholds are provided in Supplementary Note 4). This subset was processed using the same procedure as the main dataset, yielding 940 data points and enabling integration of kinetic descriptors into subsequent modeling and mechanistic analysis. In the following, we performed descriptive analyses to visualize the distribution and relationships of key features in the dataset that provided an initial overview of the parameter space and informed subsequent modeling decisions.

### Machine learning models and training

To develop a robust predictive model for methanol synthesis performance, we systematically trained and evaluated six established ML models: decision tree (DT) regression, random forest (RF) regression, categorical boosting (CatBoost), eXtreme gradient boosting (XGB), artificial neural network (ANN), and support vector machine (SVM). These models were selected for their proven applicability in catalysis research and their diverse learning frameworks^20,22,49,50^. The dataset was randomly split into training and validation subsets (70:30 split), and model performance was evaluated using mean squared error (MSE), mean absolute error (MAE), coefficient of determination (R^2^), and residual error distributions (Supplementary Note 5).

### Fixed-bed validation experiment

A controlled fixed-bed experiment was performed to evaluate the ML-derived GHSV-*E*_app_ relationship under low-pressure CO_2_ hydrogenation conditions. A commercial Cu/ZnO/Al_2_O_3_ catalyst was purchased from Alfa Aesar. The nominal supplier-reported composition was 63.5 wt% CuO, 24.7 wt% ZnO, 10.5 wt% Al_2_O_3_, and 1.3 wt% MgO. High-purity CO_2_, H_2_, and N_2_ gases were used for all experiments.

Catalytic tests were conducted in a continuous-flow fixed-bed reactor operated at 1 bar. A total of 4.0 g of catalyst was packed in the tubular reactor and fixed between quartz-wool plugs. The reactor temperature was controlled using an electric furnace, and the bed temperature was monitored by a thermocouple placed close to the catalyst bed center. Gas flow rates were regulated using mass flow controllers.

Before performance measurements, the catalyst was reduced in situ under H_2_ at a total flow rate of 50 cm^3 ^min^−1^. The temperature was raised to 350 °C and maintained for 5 h. After activation, the reactor was cooled to 160 °C and purged with N_2_ for 30 min. The CO_2_/H_2_ feed ratio was then set to 1:3, and catalytic performance was measured at mass-normalized space velocities of 750, 1500, and 3000 cm^3^ g_cat_^−1^ h^−1^. The term GHSV was retained for consistency with the literature-derived dataset. At each space velocity, the temperature was increased from 160 to 240 °C in 10 °C increments. At each temperature, the reactor was stabilized for 20 min before the outlet stream was analyzed for 30 min. Each condition was measured at least three times.

The reactor outlet was analyzed using an online gas chromatograph (GC-2014, Shimadzu). Methanol was quantified using a flame ionization detector equipped with an HP-PLOT Q column. Permanent gases were quantified using a thermal conductivity detector equipped with Molecular Sieve and Porapak Q columns. Methanol formation was quantified using an external calibration curve. A 1.0 mL GC sampling loop was used, and STY_MeOH_ was calculated from the methanol amount per injection, total outlet flow rate, catalyst mass, and methanol molar mass. The resulting STY_MeOH_ values were reported in g_MeOH_ kg_cat_^−1^ h^−1^.

Apparent activation energies for methanol formation were extracted independently at each GHSV by fitting ln(STY_MeOH_) against 1/*T*. Only the quasi-linear temperature region from 160 to 210 °C was used for fitting. *E*_app_ was calculated as −*R* multiplied by the slope of the ordinary least-squares fit, with *R* = 8.314 J mol^−1^ K^−1^. For visualization, Arrhenius plots were presented as ln(STY_MeOH_) versus 1000/*T*.

## Results

### Mapping the experimental space for machine learning guidance

To provide physical intuition into the diversity of reaction conditions and catalyst compositions historically investigated, we first mapped the multidimensional experimental space captured in the curated dataset before model training. The direct hydrogenation of CO_2_ to methanol was first demonstrated in the 1990s by Lurgi and independently by Mitsui using commercial and tailored Cu/ZnO-based catalysts^51,52^. Given this historical context, we selected 1995 as a starting point for our dataset curation, marking the onset of a substantial expansion in research interest and experimental diversity.

**Fig. 3:**
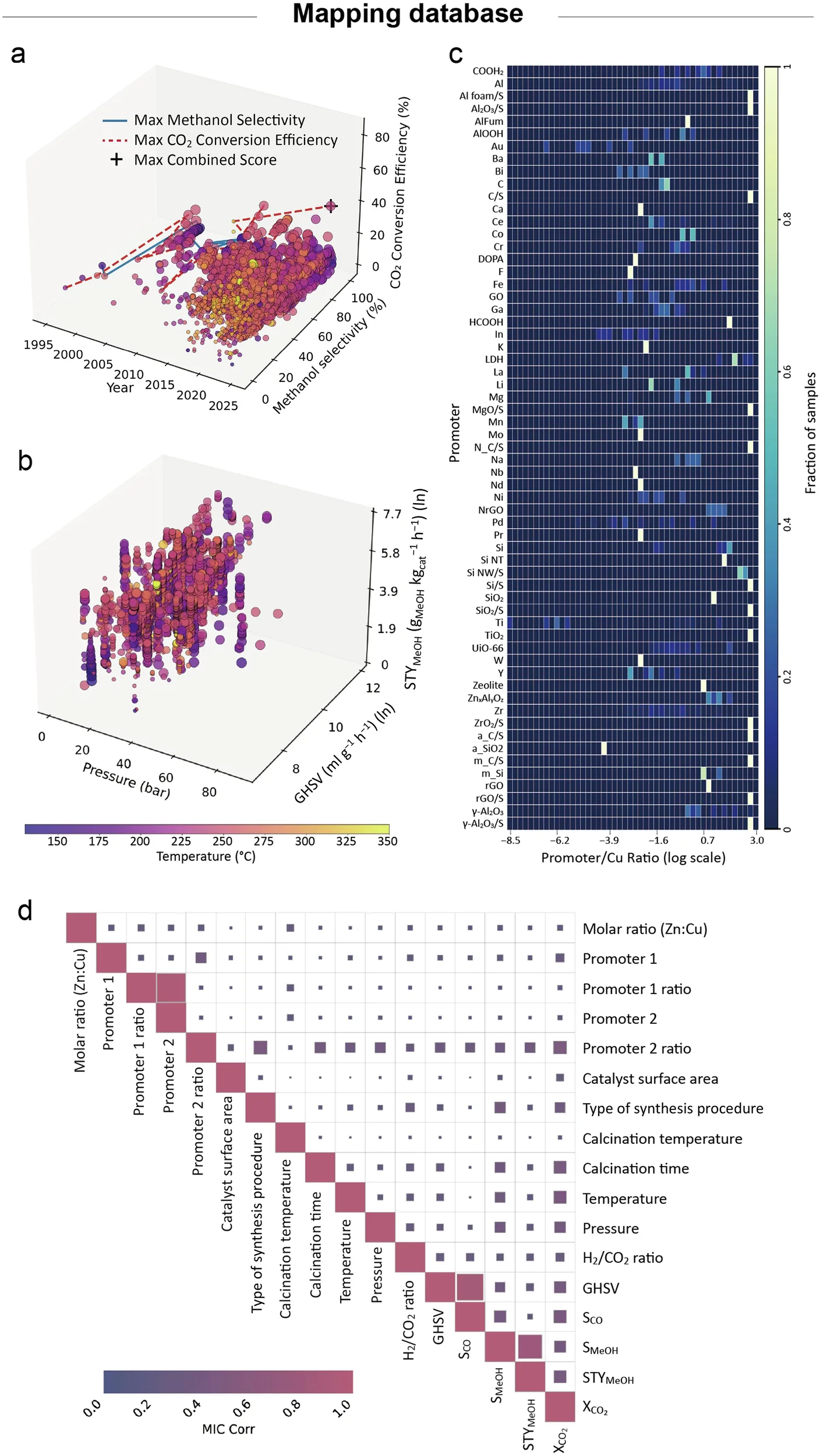
Mapping the dataset of CO_2_ hydrogenation to methanol on Cu/ZnO-based catalysts. **a** Maximum methanol selectivity (solid blue), maximum CO_2_ conversion efficiency (dashed red) and combined performance score (“+”) plotted against publication year (1995–2025). **b** Three-dimensional operating-condition map showing reaction pressure, GHSV and methanol space-time yield (STY_MeOH_) for all data points. Marker size and color in both (a) and (b) reflect methanol selectivity and reaction temperature (140–350 °C), respectively. **c** Distribution of promoter chemistries and loadings. Heatmap of the fraction of samples (color scale) versus promoter identity and promoter/Cu molar ratio on log scale. **d** Heatmap of pairwise MIC values between catalyst composition, synthesis descriptors, reaction conditions and performance metrics. Square area and color intensity both scale with MIC magnitude.

The complexity of CO_2_ hydrogenation is compounded by multiple interrelated variables, including catalyst bulk composition, preparation methods, and reaction conditions, all intricately influencing observed catalytic outcomes^53–58^. Over the past three decades, researchers have explored a vast parameter space, including reaction temperatures (140–350 °C)^59,60^, pressures (1–100 bar)^53,61^, H_2_/CO_2_ feed ratios (3:1 up to excess hydrogen)^62^, variations in Cu loading, surface area, and promoters^63–65^. As shown in Fig. 3a and b, reaction temperatures typically ranged from approximately 140 °C, associated with low-temperature kinetic studies^37,66^, to around 300 °C, constrained by equilibrium shifts toward the competing reverse water-gas shift (RWGS) reaction (CO_2_ + H_2_ → CO + H_2_O)^67–69^. Experimentally tested pressures varied dramatically, spanning from near-atmospheric conditions (ca. 1 bar in exploratory laboratory settings) to approximately 100 bar under highly optimized conditions, though industrial standards usually settle around 50–80 bar (Fig. 3b)^53,61,70–74^. Correspondingly, recorded CO_2_ conversions show substantial variability, from near-zero in mild conditions to peak efficiencies of ca. 50% at optimized high-pressure and high-temperature scenarios. Similarly, methanol selectivity ranged from minimal values (ca. 20%), dictated by competing RWGS to near-total selectivity under specific catalytic formulations and conditions favoring methanol formation (Fig. 3b). Notably, research on CO_2_ hydrogenation to methanol experienced a decade-long stagnation but has resurged with remarkable intensity over the past five to six years, as evidenced by the concentration of high-performance data points in recent publications (Fig. 3a). The dataset reveals a clear trend toward optimized conditions, with studies progressively converging on parameters that approach industrial relevance, especially around moderate temperatures (ca. 235 °C).

Apart from the reaction conditions, the bulk composition (Cu:Zn ratio and any promoters)^75,76^ and the preparation method (e.g., co-precipitation conditions, calcination, and reduction parameters)^77–80^ of catalysts critically determine observed CO_2_ conversion and product selectivities. Typical catalyst formulations feature substantial Cu excess, predominantly adopting a Cu:Zn ratio near 70:30 (with approximately 10 wt% Al_2_O_3_)^81^. The Cu content in the catalysts ranges roughly from approximately 20 to 80 mol% with the balance primarily ZnO and minor promoters, covering both Cu-rich and Zn-rich formulations. Moreover, as shown in Fig. 3c, more than fifty chemically distinct promoters have been screened on the Cu/ZnO platform, spanning eight orders of magnitude in promoter-to-Cu molar ratio. Despite decades of research, critical uncertainties remain regarding the effect of Cu particle size^82^, the role of the promoter/support^83^, the identity of the active site (Cu–ZnO interface versus CuZn alloy)^84–89^, and whether the reaction proceeds via formate or RWGS pathways^10,56,90–93^. Advanced characterization studies continue to provide new insights, but a unifying understanding and predictive design rules for Cu/ZnO-based catalysts are still lacking.

To elucidate the latent structure among input features and catalytic performance metrics, we evaluated pairwise associations using the maximal information coefficient (MIC), a metric well-suited for capturing nonlinear and non-monotonic dependencies relevant to catalysis (Fig. 3d)^94^. The majority of compositional features exhibit only weak correlations, indicating that variables such as Zn/Cu ratio, promoter identity and loading, calcination conditions, synthesis procedures, and surface area were largely varied independently across the dataset. Notably, the Cu-normalized ratio of promoter 2 displays moderate correlations with both synthesis and reaction parameters, suggesting occasional co-optimization in the literature. Reaction conditions, including temperature, pressure, H_2_/CO_2_ ratio, and GHSV, form a coherent sub-cluster with modest mutual correlations, reflecting common empirical trends toward increasing all four to achieve higher throughput. Among these, pressure positively correlates with S_MeOH_, STY_MeOH_, and X_CO2_, but inversely with S_CO_. In contrast, temperature promotes CO_2_ conversion and CO formation, while suppressing methanol selectivity, indicating a kinetic trade-off between conversion and product specificity. The sparsity of high-MIC values supports interpretable machine learning by indicating low collinearity while preserving key higher-order interactions governing catalytic performance.

### Training and selecting machine learning models

**Fig. 4:**
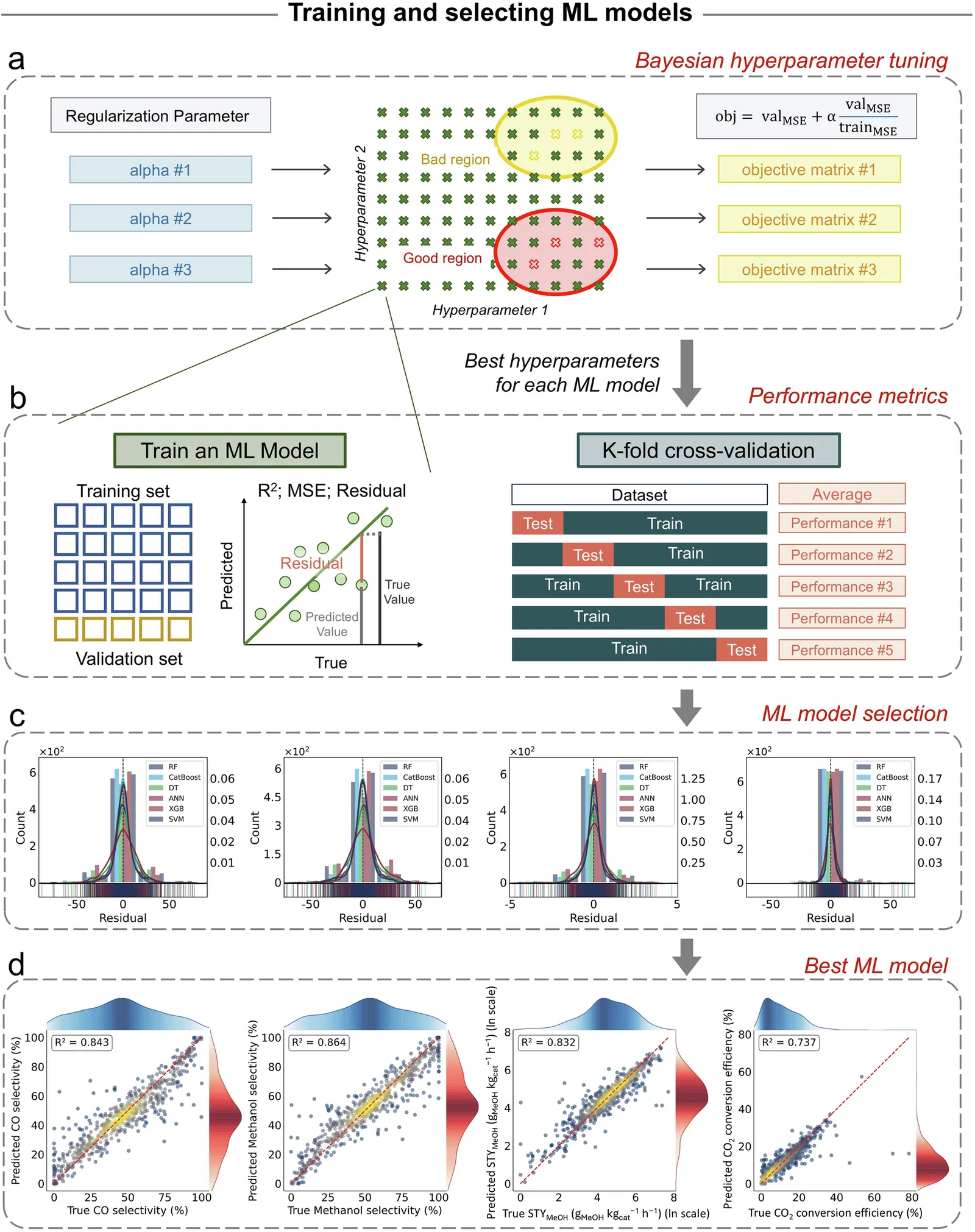
Workflow for training and selecting the optimal ML model. **a** Bayesian hyperparameter tuning. For each regularization strength (α), Optuna performs 1400 Tree-structured Parzen Estimator trials, directing the search from high-error (yellow) to low-error (red) regions and returning an objective-value matrix of best hyperparameters. **b** Robust performance assessment. Each optimal configuration is retrained to obtain MSE, MAE, R^2^, and residual statistics and validated under a 5-fold stratified scheme. **c** Residual histograms for DT, RF, ANN, XGB and CatBoost at α = 0.01 reveal that CatBoost gives the narrowest, zero-centered error profiles, whereas DT and RF show broader tails. **d** Parity plots of the validation dataset for the selected CatBoost model at α = 0.01. Predicted versus experimental values for S_CO_, S_MeOH_, STY_MeOH_ (ln scale) and X_CO2_ align closely with the 1:1 line (dashed).

A critical step in developing high-fidelity ML models is hyperparameter optimization^95^. Traditional strategies like grid and random search are often computationally prohibitive and inefficient as they do not learn from past evaluations^96^. To overcome these limitations, we employed Bayesian optimization, a probabilistic framework that iteratively refines a surrogate model of the objective function based on observed performance^97^. At every iteration, the surrogate predicts the performance landscape over the hyperparameter space, and an acquisition function selects the next evaluation point by balancing exploration (sampling uncertain regions) and exploitation (refining known optima), as shown in Fig. 4a. This approach accelerates convergence toward the global optimum while minimizing redundant evaluations.

We implemented Bayesian optimization via Optuna^98^, which employs a Tree-structured Parzen Estimator and adaptively selects hyperparameters based on expected improvement. To mitigate the risk of overfitting, a common challenge in ML^49^, we introduced a regularization parameter (α) into the objective function:

1 $${{{\rm{obj}}}}={{{\rm{va}}}}{{{{\rm{l}}}}}_{{{{\rm{MSE}}}}}+{{{\rm{\alpha }}}}\frac{{{{\rm{va}}}}{{{{\rm{l}}}}}_{{{{\rm{MSE}}}}}}{{{{{\rm{train}}}}}_{{{{\rm{MSE}}}}}}$$

Here, train_MSE_ and val_MSE_ are the MSEs on the training and validation sets, respectively. This function penalizes models where the validation error significantly exceeds the training error, thus promoting better generalization. For each model, we performed Bayesian optimization for 1400 trials across four different *α* values (0, 0.01, 0.03, 0.07), as shown in Fig. S2. The optimal hyperparameter configurations identified for each model at each α value were then retrained and subjected to rigorous 5-fold cross-validation to ensure the robustness of their performance (Fig. 4b).

The predictive performance for S_CO_, S_MeOH_, STY_MeOH_, and X_CO2_, evaluated by validation and cross-validated *R*² together with overfitting indices across six ML models at different α values, was summarized in Table 2. Detailed metrics, including MAE, MSE, and *R*² for both training and validation sets, were shown in Figs. S3–S6. Although the unregularized CatBoost and XGB models yielded the highest raw validation scores, they also exhibited pronounced overfitting, with MSE ratios of 21.02 and 56.75, respectively. We found that a small regularization strength (α = 0.01) markedly alleviated overfitting while largely preserving predictive accuracy, whereas further increases in α led to a gradual decline in both validation and cross-validated performance. A full comparison of fold-averaged MSE and *R*² across all models and α values was provided in Figs. S7–S10. Based on this comparison, CatBoost with α = 0.01 emerged as the optimal choice, showing the best overall trade-off between accuracy and generalizability and the highest CV *R*² among the regularized models (0.83). The error distributions on the validation set for the best-tuned version of each model (Fig. 4c) further supported this selection, with CatBoost displaying residuals tightly centered around zero across all four targets. The predictive capability of the selected CatBoost model was further visualized in the parity plots in Fig. 4d and Fig. S11 for the validation and training sets, respectively. The corresponding training- and validation-set parity plots for the other optimized models, including RF, DT, ANN, XGB, and SVM, are provided in Figs. S12–S21 for completeness. Specifically, the CatBoost model achieved validation-set R² values of 0.843 for S_CO_, 0.864 for S_MeOH_, 0.832 for STY_MeOH_, and 0.737 for X_CO2_ (Fig. 4d and Table 2).

**Table 2:** Validation and cross-validated R^2^ and overfitting indices for six ML models at different regularization strengths (α)

Model	${{{\rm{\alpha }}}}$	R^2^ (Valid)				Overfitting		CV (R^2^)
		S_CO_	S_MeOH_	STY_MeOH_	X_CO2_	MSE Ratio^†^	R^2^ difference ^‡^	
RF	**0**	**0.749**	**0.775**	**0.752**	**0.679**	**3.85**	**0.19**	**0.75**
	0.01	0.749	0.775	0.752	0.679	3.82	0.19	0.75
	0.03	0.738	0.756	0.733	0.662	2.81	0.17	0.73
	0.07	0.706	0.723	0.716	0.641	2.12	0.15	0.69
CatBoost	0	0.867	0.887	0.858	0.755	21.02	0.15	0.85
	**0.01**	**0.843**	**0.864**	**0.832**	**0.737**	**4.14**	**0.13**	**0.83**
	0.03	0.808	0.834	0.818	0.719	2.53	0.12	0.80
	0.07	0.786	0.812	0.806	0.707	2.11	0.11	0.78
DT	0	0.624	0.670	0.631	0.564	4.32	0.29	0.58
	**0.01**	**0.620**	**0.667**	**0.629**	**0.559**	**3.81**	**0.28**	**0.58**
	0.03	0.555	0.603	0.629	0.550	2.21	0.22	0.54
	0.07	0.545	0.601	0.570	0.492	1.54	0.14	0.51
ANN	**0**	**0.655**	**0.647**	**0.675**	**0.632**	**1.60**	**0.12**	**0.67**
	0.01	0.605	0.633	0.681	0.629	1.38	0.08	0.66
	0.03	0.629	0.650	0.639	0.592	1.50	0.11	0.68
	0.07	0.645	0.660	0.693	0.643	1.54	0.10	0.66
XGB	0	0.854	0.876	0.867	0.760	56.75	0.16	0.85
	**0.01**	**0.822**	**0.840**	**0.833**	**0.743**	**4.56**	**0.14**	**0.82**
	0.03	0.782	0.808	0.813	0.725	2.76	0.13	0.79
	0.07	0.751	0.778	0.782	0.696	2.09	0.12	0.77
SVM	**0**	**0.762**	**0.779**	**0.777**	**0.663**	**4.03**	**0.18**	**0.76**
	0.01	0.751	0.779	0.773	0.656	3.28	0.17	0.75
	0.03	0.744	0.769	0.766	0.659	2.98	0.17	0.75
	0.07	0.694	0.717	0.729	0.656	2.00	0.14	0.71

^†^*MSE Ratio* mean(MSE_valid_/MSE_train_) across S_CO_, S_MeOH_, STY_MeOH_, and X_CO2_; ^‡^*R² difference* mean(R^2^_train_ – R^2^_valid_) across the same four targets.

Concretely, our framework enables automated model tuning and selection across multiple ML algorithms, with regularization to mitigate overfitting. By combining quantitative metrics with residual analysis, CatBoost emerges as the top performer, offering the best balance of low bias and tight error spread across all four targets. These results confirm its accuracy and generalizability, establishing a robust foundation for SHAP-based mechanistic interpretation in what follows.

### Translating ML-derived insights across catalytic chemical space

**Fig. 5:**
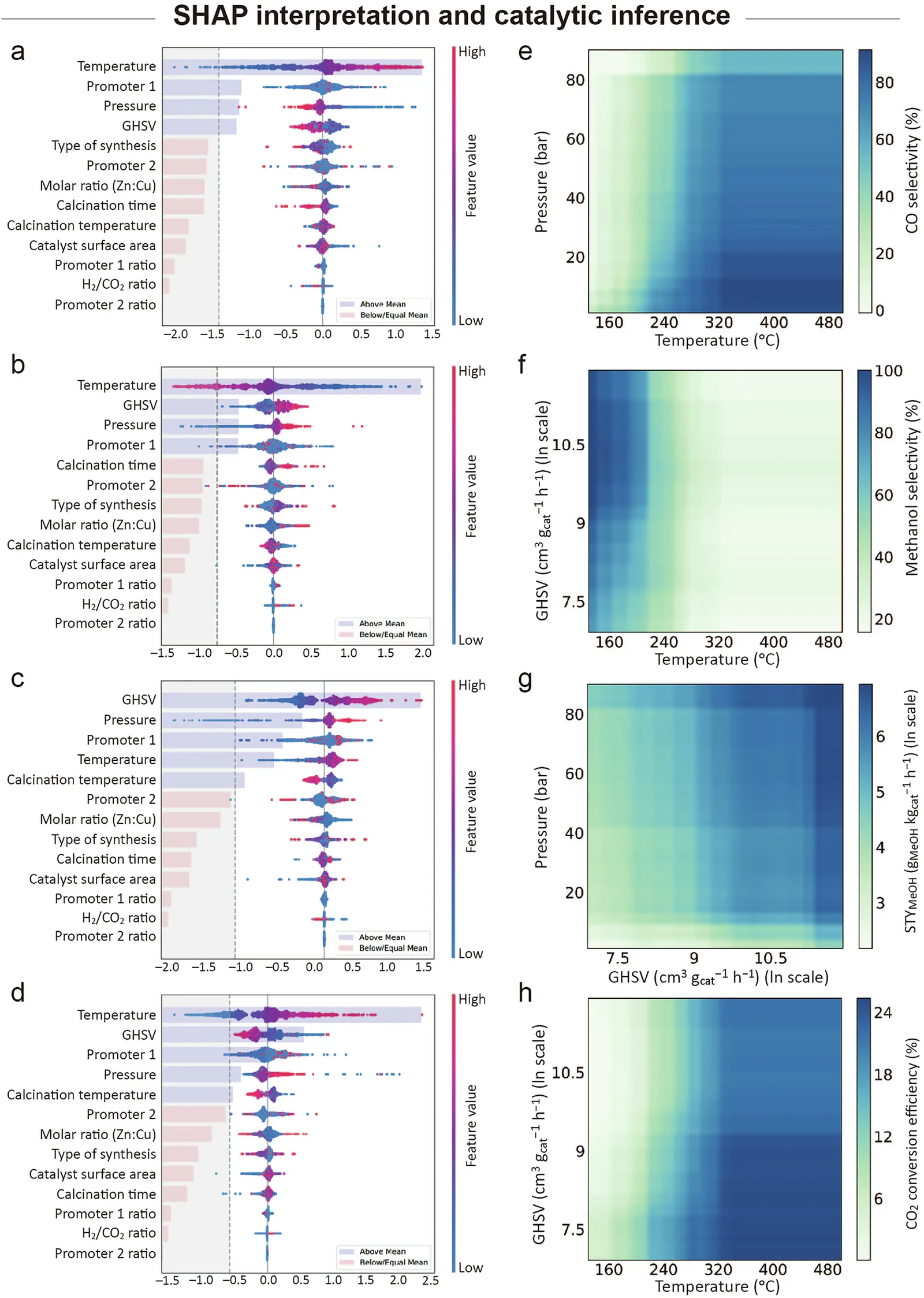
Translating ML insights to catalytic chemical space. **a**–**d** SHAP summary plots of the optimal CatBoost model, showing feature importance and directional impact for (**a**) S_CO_, (**b**) S_MeOH_, (**c**) STY_MeOH_ (ln scale), and (**d**) X_CO2_. Each dot represents one literature data point; color denotes the relative feature value, and the horizontal position indicates whether that feature value contributes positively or negatively to the predicted output. **e**–**h** SHAP-guided pairwise inference heatmaps constructed from the most influential continuously varying operating descriptors for each target. Accordingly, (**e**) S_CO_ is mapped against temperature and pressure, (**f**) S_MeOH_ against temperature and ln(GHSV), (**g**) STY_MeOH_ against pressure and ln(GHSV), and (**h**) X_CO2_ against temperature and ln(GHSV). Promoter-combination effects are resolved separately in Fig. S23.

To extract insight from the best-performing ML model, we implemented SHAP to explain the output of the ML model and perform inference based on the dominant variables. SHAP is a unified framework based on cooperative game theory that assigns each feature a Shapley value representing its contribution to a particular prediction relative to a reference baseline^99^. For each prediction, we calculated SHAP values to determine the impact of each parameter on the performance metrics of S_CO_, S_MeOH_, STY_MeOH_, and X_CO2_. This yields an interpretation of how each input feature influences the output of the model for each experiment in the database. Positive SHAP values indicate that a given feature setting enhances the predicted value, while negative values imply suppression. For example, as shown in Fig. 5a, low temperature values (blue points) have strongly negative SHAP values for CO selectivity, whereas high temperature (red points) increases CO selectivity. Importantly, SHAP is a post-hoc explanation of a data-trained model and thus captures associations present in the compiled literature dataset rather than proving causality. Accordingly, we used SHAP primarily as a hypothesis-generating tool and corroborated mechanistic interpretations with independent physicochemical evidence and targeted validation experiments, in line with recent guidance that interpretable ML insights in catalysis should be experimentally and mechanistically validated to avoid spurious correlations^100^. Consequently, SHAP-derived relationships between operating variables and *E*_app_ (e.g., GHSV) are treated as testable hypotheses inferred from the literature dataset.

Such analysis enables a human-interpretable mapping of catalytic chemical space embedded within the high-dimensional dataset. Indeed, the SHAP analysis converts the qualitative chemical intuition into quantitative thresholds. Figure S22 summarizes the aggregate SHAP importance across the four outputs, whereas the target-specific rankings in Fig. 5a–d show that the dominant descriptor pair depends on the selected metric. Temperature remains the leading operational descriptor for S_CO_, S_MeOH_, and X_CO2_, GHSV dominates STY_MeOH_, pressure is a recurrent secondary operating lever, and promoter-related descriptors emerge as major composition-level contributors. Physically, high temperature enhances CO formation and CO_2_ conversion but suppresses methanol selectivity, reflecting the kinetic–thermodynamic tension between the exothermic methanol synthesis (ΔH = −49.5 kJ mol^−1^) and the competing endothermic RWGS pathway (ΔH = 41.2 kJ mol^−1^)^101^. Low temperature and relatively high GHSV favors methanol selectivity, whereas lower GHSV mainly extends residence time and improves STY_MeOH_ at the expense of throughput^34,102^. Pressure positively influences S_MeOH_, STY_MeOH_, and X_CO2_, while slightly lowering S_CO_^103^. Among material descriptors, a high Zn/Cu molar ratio exerts a positive effect on S_MeOH_, but a negative impact on X_CO2_, leading to an overall reduction in methanol productivity (STY_MeOH_), which suggests an optimal regime where Zn promotes Cu dispersion and stabilizes active Cu–ZnO interfacial sites^64,65,104–106^. The importance of promoter content was accurately recognized by the model. The SHAP analysis reveals that the CO_2_-to-methanol performance is not governed by isolated descriptors, but emerges from the complex interplay of high-dimensional features, including synergistic effects among reaction conditions, catalyst composition, and synthesis history.

Beyond the operating windows, promoter effects can be interpreted at the pair level. This point is especially important because promoter combinations were encoded through chemistry-informed descriptors under a canonical permutation-invariant representation, such that Promoter 1 and Promoter 2 denote two slots in the representation rather than mechanistically distinct primary and secondary roles. Accordingly, promoter effects are visualized separately in the promoter-pair response matrix (Fig. S23 and Supplementary Note 6), which provides composition-level guidance complementary to the operating heatmaps in Fig. 5. The matrix further shows that promoter combinations favorable for X_CO2_ or STY_MeOH_ do not necessarily maximize S_MeOH_ or minimize S_CO_, reinforcing the target-specific nature of catalyst optimization.

The maps (Fig. 5 and S23) allow concrete optimization suggestions to be formulated. For instance, when CO_2_ conversion efficiency is prioritized, the model points to a high-temperature, low-GHSV regime, with a representative high-conversion window at ca. 320–350 °C and ln(GHSV) ca. 7.5–8.5. Within the promoter-pair matrix, combinations such as AlOOH–m_Si, m_C/S–m_Si, AlOOH–MCM, and m_C/S–MCM fall into the most favorable X_CO2_ regions. By contrast, maximizing methanol selectivity requires the opposite operating regime, namely low temperature and high GHSV. A representative high-selectivity window is ca. 150–180 °C and ln(GHSV) ca. 9.5–11.0, while the most favorable promoter-pair regions are associated with TPABr-containing pairs such as TPABr–rGO, TPABr–Zeolite, TPABr–N_C/S, and TPABr–C. These examples highlight that catalyst optimization is strongly target-specific and that the preferred windows for conversion and methanol selectivity are fundamentally distinct.

These predictions reinforce the trade-offs revealed in SHAP analysis and highlight how explainable, regularized ML with reduced overfitting transforms three decades of data into a practical pattern for process engineers seeking to balance conversion and plant throughput in CO_2_-to-methanol synthesis. Although optimization of reaction conditions has traditionally relied on empirical tuning and mechanistic elucidation has required labor-intensive experimentation, our framework offers a data-driven shortcut to both. To test its physical relevance, we use *E*_app_ as a kinetic benchmark. In the following, we examine whether the same descriptors that predict catalytic performance can also capture mechanistic trends reflected in *E*_app_.

### Deriving kinetic insight from the ML framework

To assess the physical relevance and generalizability of our ML framework beyond empirical performance correlations, we analyzed a curated kinetic subset of apparent activation energies for methanol formation, *E*_app_. These values were extracted from the literature when explicitly reported or regenerated by fitting temperature-series methanol formation rate (typically STY_MeOH_ measured at multiple temperatures under otherwise fixed catalyst and feed conditions) to the Arrhenius equation (see Supplementary Note 4 for details). Here, *E*_app_ is defined as the empirical Arrhenius slope of the observed methanol formation rate *r*:

2 $${E}_{{{{\rm{app}}}}}=-R{\left(\frac{\partial {{\mathrm{ln}}}r}{\partial \left(1/T\right)}\right)}_{p,{y}_{i},{{{\rm{GHSV}}}}}$$

where *R* is the universal gas constant, *T* is the absolute temperature, and *p*, *y*_*i*_, GHSV denote the total pressure, feed composition (or partial pressures) for all gas-phase species *i*, and space-velocity window reported for each temperature series. As such, *E*_app_ is not expected to coincide with a unique elementary-step barrier, because the observed rate generally reflects both intrinsic kinetics and temperature-dependent coverage or inhibition effects. A compact representation of the observed rate is

3 $$r={k}_{{{\mathrm{int}}}}\left(T\right)f\left(T,\left\{{p}_{i}\right\},\left\{{\theta }_{j}\left(T,\left\{{p}_{i}\right\}\right)\right\}\right)$$

where *k*_int_(*T*) denotes the intrinsic temperature-dependent rate constant for the kinetically relevant event, while the function ${f}$ captures coverage and thermodynamic driving-force effects through the set of fractional surface coverages {*θ*_*j*_} of adsorbed intermediates *j*. Differentiation yields

4 $${E}_{{{{\rm{app}}}}}={E}_{{{\mathrm{int}}}}-R{\left(\frac{\partial {{\mathrm{ln}}}f}{\partial \left(1/{{{\rm{T}}}}\right)}\right)}_{\left\{{p}_{i}\right\}}$$

where ${E}_{{{\mathrm{int}}}}$ denotes the intrinsic Arrhenius contribution associated with ${k}_{{{\mathrm{int}}}}$, showing that *E*_app_ generally contains additional contributions whenever f varies with temperature. In heterogeneous catalysis, such variations commonly arise because adsorption equilibria and surface coverages depend on both T and $\left\{{p}_{i}\right\}$, so that changes in pressure or feed composition can systematically shift *E*_app_ even for the same catalyst. Moreover, transitions in the rate-determining step within the experimental window, potentially driven by surface coverage evolution, water inhibition, or transport constraints, can lead to curvature in Arrhenius plots. Such non-Arrhenius behavior results in variable or discontinuous apparent activation energies, which likely accounts for the broad range of literature values reported for Cu/ZnO-based catalysts, even among nominally identical formulations.

**Fig. 6:**
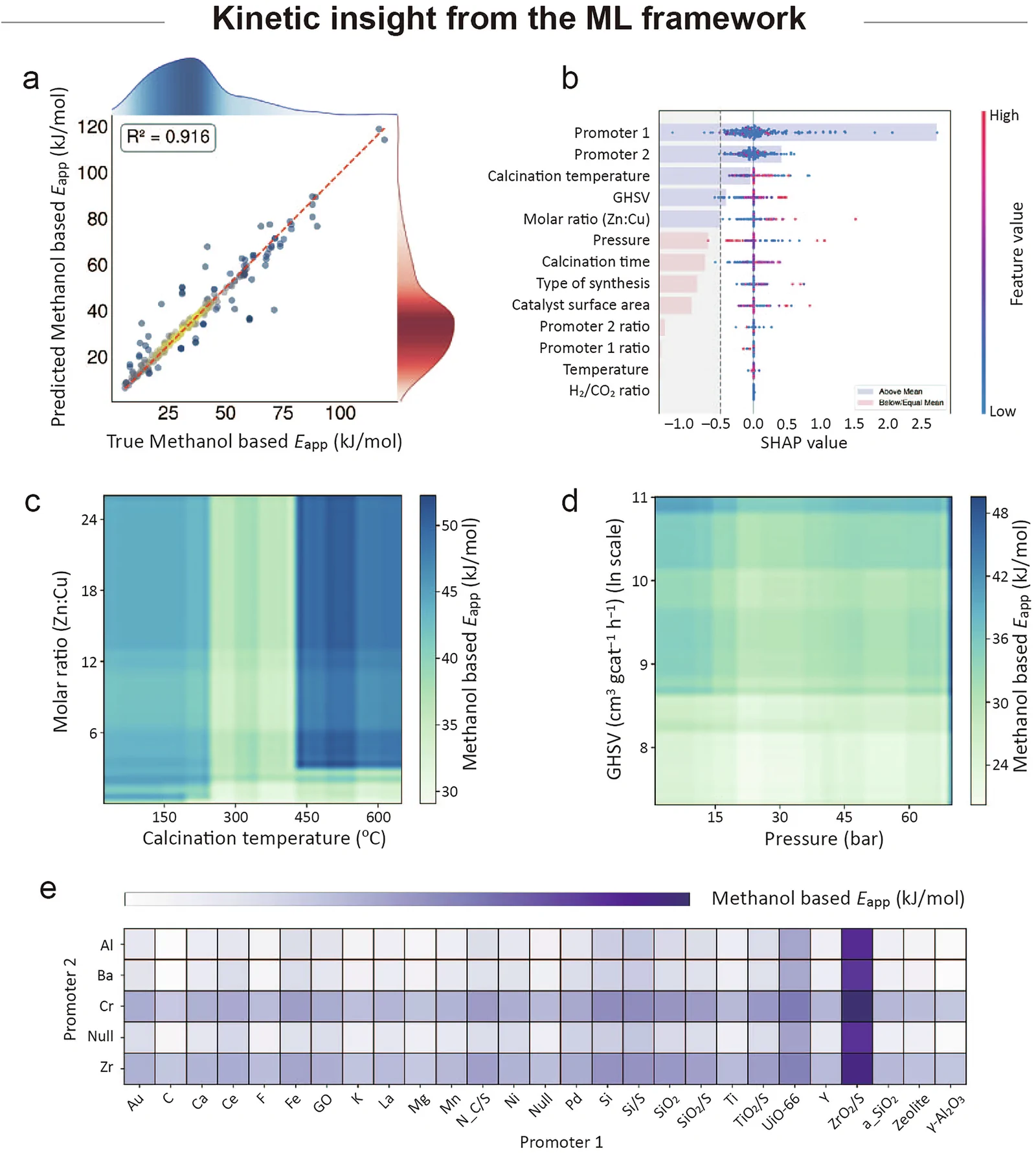
Kinetic insight from the ML model. **a** Predicted versus reported methanol-based apparent activation energy (*E*_app_), showing good agreement for the XGB model at α = 0.001. **b** SHAP values for *E*_app_ prediction. **c**, **d** SHAP-guided pairwise inference heatmaps constructed from the leading continuously varying descriptors, showing the predicted *E*_app_ variation as a function of calcination temperature and Zn:Cu ratio (**c**) and of pressure and ln(GHSV) (**d**), with the remaining variables fixed at representative reference values. **e** Promoter-pair response matrix for *E*_app_ across the literature-covered promoter space. Promoters 1 and 2 do not imply mechanistically distinct primary and secondary roles.

Therefore, *E*_app_ should be treated as a regime-sensitive kinetic observable, and the ML framework can be used to map where such regime sensitivity is most pronounced across the literature-derived feature space, which may serve as the transition points of the reaction pathway. Training on the same feature space used previously, the prediction accuracy for *E*_app_ evaluated by R^2^ and MSE of each model with different α was summarized in Table S3. As a result, the XGB model at α = 0.001 achieves high accuracy (R^2^ = 0.916; Fig. 6a). The SHAP analysis (Fig. 6b) shows that promoter chemistry emerges as the leading contributor to the kinetic landscape, followed by calcination temperature, GHSV, and the Zn:Cu molar ratio, whereas pressure, synthesis procedure, and promoter loadings exert secondary effects within the sampled domain. Notably, promoter chemistry contributes more strongly than promoter loading, indicating that elemental identity is a major discriminator of the reported apparent kinetic regimes.

To translate these ranked descriptors into interpretable kinetic maps, we generated pairwise inference heatmaps for the leading continuously varying variables while resolving promoter effects separately. Figure 6c identifies a low-*E*_app_ basin at intermediate calcination temperatures together with moderate Zn enrichment, consistent with an interfacial optimum in Cu/ZnO-based catalysts^107^. This pattern is consistent with the notion that calcination can tune catalyst structure and interfacial characteristics in ways that affect observed kinetics, while also underscoring that the literature spans heterogeneous preparation protocols and reporting practices. By contrast, Fig. 6d clearly shows *E*_app_ rises markedly upon entering the high GHSV regime, whereas pressure modulates this landscape only weakly. *E*_app_ in heterogeneous catalysis depends on surface coverage and the prevailing kinetic regime; we therefore interpret this association as indirect evidence that GHSV changes an intrinsic elementary barrier. The model associates higher GHSV with higher reported *E*_app_ in the compiled literature, which we interpret as an operational effect that may reflect residence-time-driven changes in local coverages (H* and H_2_O)^108^ and, consequently, the dominant pathway and the apparent Arrhenius slope. To directly evaluate the GHSV–*E*_app_ hypothesis suggested by the ML analysis, we additionally performed a controlled fixed-bed experiment at 1 bar using a commercial Cu/ZnO/Al_2_O_3_ catalyst by varying only the space velocity; the experimental details and discussion are provided in the subsequent section and Supplementary Information.

Because promoter identities were encoded through chemistry-informed descriptors under a canonical permutation-invariant representation, promoter effects must be interpreted at the pair level. Accordingly, Fig. 6e summarizes the predicted *E*_app_ landscape across promoter-slot combinations. The matrix indicates that the reported apparent kinetic behavior varies substantially across promoter-pair space, but these contrasts should be interpreted as screening-level guidance because promoter identity, synthesis history, and operating conditions are frequently co-varied in the literature.

### Experimental validation and cross-system transferability of the ML framework

To directly test the ML-derived hypothesis that GHSV modulates the observed kinetic barrier for methanol formation, we performed controlled fixed-bed CO_2_ hydrogenation experiments over a commercial Cu/ZnO/Al_2_O_3_ catalyst at 1 bar while varying only the flow rate. The catalyst identity, feed composition, and activation protocol were held constant, and methanol productivity was measured between 160 and 240 °C at GHSV values of 750, 1500, and 3000 cm^3^ g_cat_^−1^ h^−1^. Detailed experimental procedures, Arrhenius fitting, and mechanistic discussion are provided in Supplementary Note 7.

**Fig. 7:**
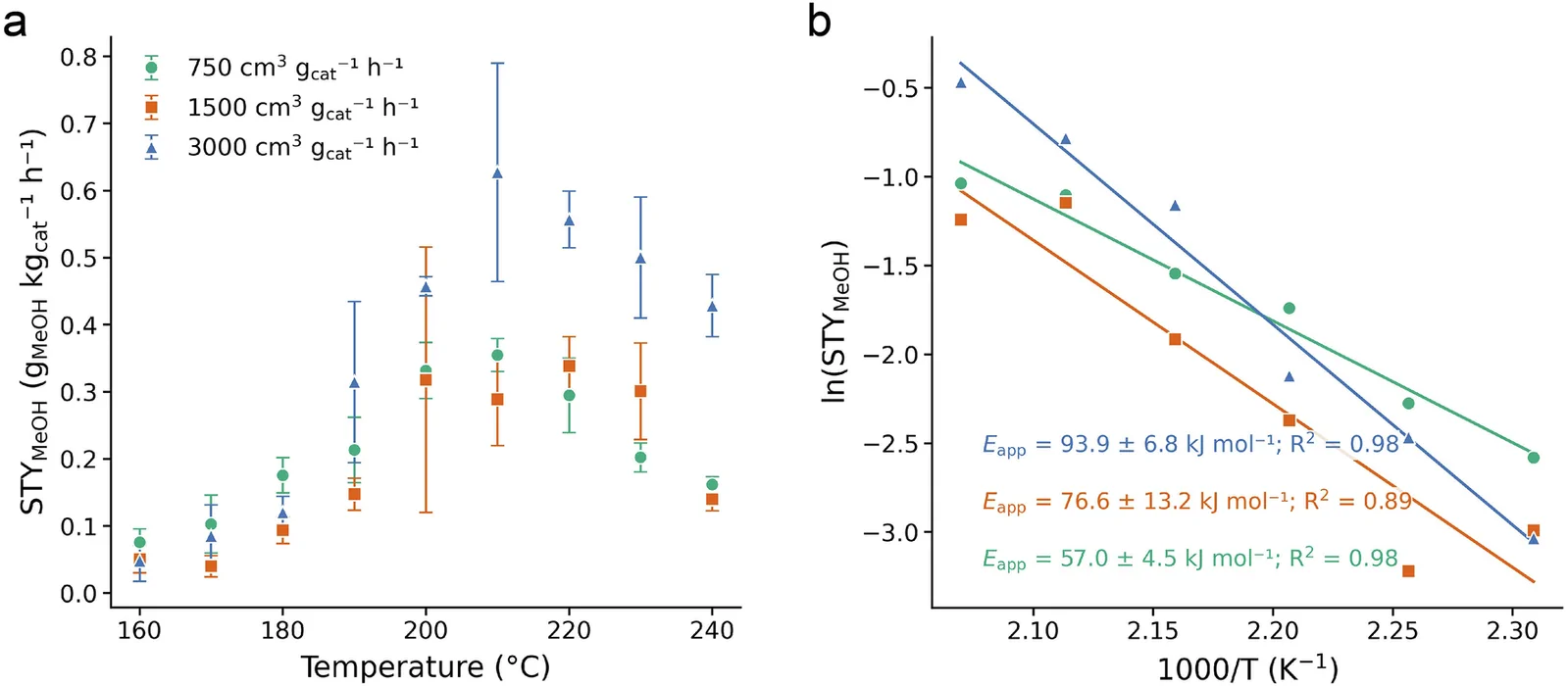
Experimental validation of the ML-derived GHSV–*E*_app_ relationship over a commercial Cu/ZnO/Al_2_O_3_ catalyst at 1 bar. **a** Methanol space-time yield, STY_MeOH_, as a function of reaction temperature at space velocities of 750, 1500, and 3000 cm^3^ g_cat_^−1^ h^−1^. Error bars represent the standard deviation of repeated measurements. **b** Arrhenius plots of ln(STY_MeOH_) versus 1000/T with linear fits applied to the quasi-linear temperature windows used for *E*_app_ extraction. The fitting windows were 160 to 210 °C. The resulting methanol-based apparent activation energies are 57.0 ± 4.5, 76.6 ± 13.2, and 93.9 ± 6.8 kJ mol^−1^, respectively.

Apparent activation energies were extracted from the quasi-linear Arrhenius regions (Fig. 7). The experimentally determined methanol-based *E*_app_ increased monotonically with GHSV, from 57.0 ± 4.5 kJ mol^−1^ at 750 cm^3^ g_cat_^−1^ h^−1^ to 76.6 ± 13.2 kJ mol^−1^ at 1500 cm^3^ g_cat_^−1^ h^−1^ and 93.9 ± 6.8 kJ mol^−1^ at 3000 cm^3^ g_cat_^−1^ h^−1^ (Fig. 7**and** S24). Because only the flow rate was changed while the catalyst, pressure, and feed ratio were fixed, this experiment isolates GHSV as an operational variable and directly corroborates the positive GHSV–*E*_app_ relationship inferred from the literature-trained ML model. Such behavior is consistent with prior heterogeneous catalysis studies showing that apparent activation energies can remain insensitive to, or become strongly modulated by, flow and residence-time changes depending on the prevailing kinetic regime and surface-coverage state^109–112^.

Direct literature on a GHSV-dependent methanol-based apparent activation energy in CO_2_-to-methanol synthesis remains limited. Under a higher-pressure operating window, Bukhtiyarova and co-workers reported nearly unchanged methanol *E*_app_ values over a commercial Cu/ZnO/Al_2_O_3_ catalyst across the investigated space-velocity range, indicating that space velocity did not perturb the controlling regime under those conditions^113^. By contrast, in other heterogeneous catalytic systems, residence time or space velocity has been shown to influence measured *E*_app_ and regime transitions^109,110^. These precedents support interpreting the present result as a flow-induced change in the observed kinetic regime.

For Cu-based CO_2_ hydrogenation specifically, recent mechanistic studies indicate that the apparent kinetics of methanol formation are highly sensitive to the local abundance of adsorbed hydrogen and water^114^. Methanol formation has been linked to methoxy hydrolysis, and water can facilitate the CH_3_O* to CH_3_OH step^108^. Accordingly, one plausible interpretation of the present data is that lowering the space velocity promotes a more water-rich and coverage-modified surface microenvironment, thereby lowering the observed Arrhenius slope for methanol formation. However, excessive space velocity shortens residence time, limiting hydrogen availability and disrupting the Cu^0^–Zn^δ+^ interface, thus shifting the system toward higher-barrier pathways (e.g., carboxyl and direct CO_2_ dissociation)^34^. Thus, controlling GHSV is essential to preserve low-barrier reactivity and avoid kinetic masking under transport-limited regimes. This interpretation is proposed at the level of observed kinetics and does not imply that the intrinsic elementary activation barrier of the catalyst itself is directly altered by flow rate.

A related practical question concerns the extent to which literature data availability and curation quality constrain the applicability of data-driven catalysis frameworks. In practice, well-studied catalytic systems remain the most accessible starting point, because the limiting factor is not the number of publications surveyed, but the number of high-quality, consistently defined data points that remain after harmonized curation. Our literature survey spans 200 publications and the model training uses 2383 curated, permutation-invariant experimental entries. This scale is moderate for machine learning on experimental catalysis data (Table S4 and Fig. S25). Across reaction classes, experimental dataset sizes span from single-digit to over ten thousand data points, indicating that datasets on the order of 10^3^ are not atypical once multiple sources are harmonized. The surveyed studies cover thermocatalysis (e.g., PDH, DRM, WGS, OCM, etc.), electrocatalysis (e.g., CO_2_RR, NRR, OER/HER), photocatalysis, organic catalysis, and process-related datasets. CO_2_ thermocatalysis (e.g., CO_2_-to-methanol and CO_2_-to-olefins) is listed separately. To our knowledge, this is among the most extensive curated datasets focused on CO_2_-to-methanol synthesis over Cu/ZnO-based catalysts.

To distinguish the transferability of the workflow from the availability of sufficiently curated data, we applied the same end-to-end protocol of data schema definition, curation and standardization, model training, SHAP-based interpretation, and inference to an independent electrocatalytic system, electrochemical CO_2_ reduction (CO_2_RR), using a curated dataset of 379 literature-derived entries. Full details of the CO_2_RR dataset, pre-processing, model validation, SHAP analysis, and representative inference maps are provided in Supplementary Note 8. For this auxiliary CO_2_RR transferability test, the descriptor distributions after preprocessing are shown in Fig. S26 before applying the same tuning and validation protocol. CatBoost was selected as the final model, and the α = 0.3 setting was retained as the best accuracy–generalization compromise (Table S5). Interpretability analysis further showed that the selected model recovered chemically meaningful descriptor–performance relationships for the selectivity of Cu-based CO_2_RR. SHAP summaries and pairwise inference maps in Fig. S27 identify applied potential and particle size as leading descriptors and reveal output-specific, non-monotonic response surfaces for H_2_, CO, C_1_, and C_2_+ . The categorical inference map in Fig. S28 further shows that active phase and catalyst shape jointly redistribute the predicted product fractions.

Taken together, the targeted GHSV–*E*_app_ experiment and the auxiliary CO_2_RR case study validate the framework at experimental corroboration within the benchmark methanol system and cross-system transferability across catalytic regimes.

## Discussion

By systematically curating three decades of experimental data for Cu/ZnO-catalyzed CO_2_-to-methanol synthesis, we demonstrate, in principle, that the data from existing publications can be mined to build predictive ML models. If successful, such a model could serve as a meta-experiment, integrating diverse findings to highlight which factors most strongly affect performance and to guide optimal catalytic reaction system design^22,115^. The central advance is therefore not merely the application of machine learning to methanol synthesis, but the integration of catalyst composition, synthesis history, operating conditions, catalytic outputs, and apparent kinetics within a single experimentally grounded framework. In a field where optimization is still often pursued through stepwise empirical tuning and individual studies cover only limited regions of parameter space, this integrated view is itself a substantive result. The present workflow shows that literature-derived data can do more than support retrospective correlation analysis; when carefully curated and coupled with low-overfitting models, they can generate testable hypotheses and actionable guidance for catalyst and process design.

The model outcomes are notable both for what they confirm and for what they newly reveal. On the one hand, the framework recovers established chemical intuition: higher pressure generally favors methanol formation, elevated temperature promotes CO formation and CO_2_ conversion at the expense of methanol selectivity, and the Cu/Zn balance remains a critical descriptor of catalyst performance. On the other hand, the SHAP-guided inference maps expose non-linear interactions that are difficult to extract from isolated reports, including saturation behavior in promoter loading and strongly target-dependent trade-offs across temperature, pressure, and GHSV. Particularly important is the emergence of a broad STY_MeOH_ regime in which industrially relevant methanol productivity can be approached without relying on conventionally high pressure. From a reaction-engineering perspective, this result suggests that process intensification may be achievable through coordinated control of flow and pressure rather than pressure escalation alone.

A second major outcome is the extension of the framework from performance prediction to regime-sensitive kinetic interpretation. The predicted GHSV ridge and pressure relationship with *E*_app_ do not, by themselves, prove an elementary-step switch, but they do provide a structured basis for targeted mechanistic inquiry. The dedicated fixed-bed validation at 1 bar was carried out when only GHSV was varied. The monotonic increase in methanol-based *E*_app_ with space velocity directly supports the ML-inferred trend and indicates that flow can reshape the observed kinetic regime. In this sense, the framework makes such experimentation more selective, more hypothesis-driven, and better informed by the collective structure of the literature.

The auxiliary CO_2_RR case study further clarifies the scope of the method. Transferability was retained despite the smaller dataset and the substantially different descriptor space, indicating that the workflow is not intrinsically limited to Cu/ZnO methanol chemistry, but to catalytic systems for which sufficiently structured experimental data can be assembled. This distinction is important. The present study should not be interpreted as evidence that literature-driven interpretable ML is uniformly deployable across all catalytic problems. Its most realistic application domain is mature, data-rich chemistries in which variables can be harmonized across sources and represented within a consistent schema.

At the same time, the present framework has clear boundaries that should be stated explicitly. First, the models necessarily inherit the strengths and weaknesses of the literature from which they are built. Uneven coverage of catalyst formulations and operating windows, inconsistent reporting standards, publication bias, and the scarcity of explicitly negative results all shape the learned response surface. Second, although the curated dataset captures a broad range of catalyst, synthesis, and reaction descriptors, several mechanistically decisive variables remain absent or only weakly represented, including oxidation-state distributions, Cu particle morphology, interface density, and specific Cu–Zn^δ+^ motifs under reaction conditions. Third, the SHAP relationships reported here are associative rather than causal. Their value lies in converting a high-dimensional experimental record into chemically intelligible hypotheses, but they should not be overinterpreted as direct mechanistic proof. The same caution applies to literature-derived *E*_app_ values, which reflect operating-window-dependent observed kinetics.

These limitations also point directly to the next stage of development. The predictive and explanatory power of literature-driven ML should improve as standardized reporting, digitized supporting information, and machine-readable kinetic datasets become more common. At the data-curation stage, however, fully automated extraction remains limited by heterogeneous reporting formats, nonstandard catalyst nomenclature, graphical data, and context-dependent definitions of performance metrics. Recent progress in automatic text summarization and machine-assisted literature reviewing suggests that LLM-assisted tools could accelerate literature triage, table and figure identification, and preliminary record extraction, but quantitative catalysis datasets will still require human-in-the-loop verification of units, reconstructed outputs, and chemical consistency^116^. Incorporating richer descriptors from operando spectroscopy, microscopy, and physics-informed surrogate variables should help narrow the gap between experimentally reported inputs and the true catalyst state under reaction conditions. More broadly, coupling interpretable ML with targeted closed-loop experimentation and emerging reaction-engineering strategies may provide a more efficient route to catalyst discovery than either retrospective data mining or isolated experiments alone. Viewed in this way, the value of the present work lies not only in the specific maps obtained for Cu/ZnO-based methanol synthesis, but in demonstrating how decades of fragmented experimental effort can be reassembled into forward-looking design principles.

## Supplementary information

Transparent Peer Review file

Supporting Information

## Data Availability

All data supporting the results of this study are available within the paper and its Supplementary Information section. The curated dataset is available at https://github.com/MoYan-101/CommsEng. Additional raw data are available from the corresponding author upon reasonable request.

## Code availability

The codes used in this study are available at https://github.com/MoYan-101/CommsEng.
