# Supplementary material for: A machine learning perspective on three decades of methanol synthesis: research framework and experimental operation insights
Source: Commun Eng. 2026 Jun 10;5:162. doi: 10.1038/s44172-026-00706-4 (PMC13594224; doi:10.1038/s44172-026-00706-4)
Supplement: Supplementary file 1 [file 44172_2026_706_MOESM1_ESM.pdf]

nature portfolio

Peer Review File

A Machine Learning Perspective on Three Decades of Methanol Synthesis: Research Framework and Experimental Operation Insights

Corresponding Author: Professor Taesung Kim

This file contains all reviewer reports in order by version, followed by all author rebuttals in order by version.

Version 0:

Reviewer comments:

Reviewer #1

(Remarks to the Author)
In this paper Duan, Kim , et al. demonstrated an application of machine learning for tuning experimental parameters rather than using intuition-guided as in traditional method which could be very useful for industrial process in reaction optimization. The data was well curated from 200 papers since 1995 of CO2-to-methanol synthesis using Cu/ZnO-based catalysts and activation energy (Ea). 5 models were trained to predict CO2 conversion efficiency , methanol selectivity, CO selectivity and methanol space-time yield. This paper can be accepted after a major revision.
Here are my comments.

1. In Page 4 Line 111-113, the authors used mean imputation to fill data such as surface area or GHSV, can these values be estimated by a more scientific way? Such as interpolation or using Monte Carlo methods.
2. Why the reaction temperature used is in degree Celsius instead of Kelvin, does it yield difference results?
3. In figure 2b, it is not clear, as the author mentioned that missing data was filled, but “Missing and outlier data” was dropped, why there are still missing data at this point as you have dropped in the previous process, more explanation needed.
4. As you only have around two thousands points, Support Vector Machine (SVM) could provide better accuracy in small dataset (Please see https://www.nature.com/articles/s41467-020-19594-z)
5. Figure 6 has too much details, it is extremely hard to interpret the results and understand to results. This figure can be rearranged or moved to supporting materials if there is no better way to depict the results.
6. The author should have a demonstration or suggestion of how to maximize/minimize the output for example, with combination of promoter A and promoter B in temperature C can maximize CO2 conversion efficiency, I suggested graphs of a heatmap where x and y are 2 most feature importance and performance metrics as heat points, so the chemists who are not ML/AI based can also understand.
7. Fix the typo in Figure 7a, it should be Methanol-based instead of Methonal-based.
8. The author can try using pre-train embedding model (such as Chemberta) instead of one-hot encoding, this can enhance model’s performance as the embedding vector already contains chemical properties.
9. The author should publish the code used in this paper.

Reviewer #2

(Remarks to the Author)
This study integrates 2,476 experimental data points from 200 literature sources between 1995 and 2025, establishing a machine learning dataset that covers catalyst composition, synthesis methods, reaction conditions, and performance indicators. By employing Bayesian optimization, regularization strategies, and SHAP interpretive analysis, it reveals the nonlinear relationships in methanol synthesis. However, the following aspects need to be further revised and improved.
1. There are already many studies on the application of machine learning to accelerate the optimization design of catalysts for CO2-to-methanol. The uniqueness of this research needs to be described and discussed in detail. The uniqueness of this study needs to be elaborated and discussed in detail.
2. In the discussion section, add an analysis of the limitations of the data, for instance, explain that the literature data might

have omitted failed experiments or extreme conditions. Additionally, it is suggested to supplement the estimation methods for unreported variables (such as particle size) or future work directions, such as combining characterization data.
3. In the discussion section, add an analysis of the limitations of the data, for instance, explain that the literature data might have omitted failed experiments or extreme conditions. Additionally, it is suggested to supplement the estimation methods for unreported variables (such as particle size) or future work directions, such as combining characterization data.
4. Although SHAP analysis reveals the importance of variables, some of its conclusions (such as the relationship between GHSV and Ea) lack experimental verification and may overly rely on correlation.
5. The application of the developed machine learning model still needs to be enhanced. Additionally, could experimental verification be introduced?

Reviewer #3

(Remarks to the Author)
I was pleased to read the study by Yan et al., entitled “A Machine Learning Perspective on Three Decades of Methanol Synthesis: Research Framework and Experimental Operation Insights”. The study aims to apply machine learning to identify optimal operating conditions in heterogeneous catalysis. The authors claim that their data-driven approach uncovers previously underexplored operating windows for achieving high methanol productivity in CO_2-to-methanol synthesis. Furthermore, they argue that the apparent activation energy should not be regarded as an intrinsic material property, but rather as a reaction-condition-dependent observable. Based on the fundamental limitations outlined in the following comments regarding generality, scope, and overclaimed novelty, I recommend rejection of the manuscript in its current form, with the possibility of resubmission should the authors substantially broaden the applicability of the framework and revise the central conceptual claims.

Comment 1: The machine-learning framework is rigorous. However, the generality of the authors’ claims should be reconsidered. The approach relies on an unusually large body of literature data (200 papers spanning three decades), which limits its practical applicability. For most reactions and emerging catalyst systems, such extensive datasets are unlikely to exist, restricting the framework’s use as a broadly deployable guidance tool. The main strength of the approach lies in extracting design rules from mature, well-studied catalytic systems.

Comment 2: Closely related to Comment 1, the analysis is restricted to a single catalyst family (Cu/ZnO-based systems) and a single reaction (CO_2-to-methanol synthesis), resulting in a limited exploration of chemical degrees of freedom. While the framework is valuable for rationalizing trends within this well-established system, its practical generality remains unproven. To justify the breadth of the claims and the scope of the journal, the framework should be extended to include automated data curation and demonstrated on at least one additional catalytic system. If such an extension is not practically feasible, this would itself highlight the limited applicability of the framework, and the manuscript cannot be accepted in its current form.

Comment 3: The authors claim to reveal that the activation energy (Ea) is a reaction-condition-dependent property. This claim is not acceptable. By definition, Ea is an empirical parameter characterizing the temperature dependence of a reaction rate coefficient and is therefore inherently condition dependent. Consequently, this statement does not constitute a novel finding, and the importance of the work cannot be based on this claim.

Version 1:

Reviewer comments:

Reviewer #1

(Remarks to the Author)
The manuscript can be accepted for publication in its current form, as the authors have clearly addressed and satisfactorily explained all comments and questions.

Reviewer #2

(Remarks to the Author)
I am fully satisfied with the current version of the manuscript and recommend it for acceptance.

Reviewer #3

(Remarks to the Author)
Thank you very much for the detailed rebuttal and the substantial revisions to the manuscript. The authors have addressed most of the previously raised concerns satisfactorily. A few points remain, which are outlined below. The comment numbering follows the original numbering to avoid confusion.

Comment 4 (related to Comment 1):
Thank you for the detailed response and the additional case study. While the inclusion of a smaller dataset is appreciated, both examples still rely on well established catalytic systems supported by decades of accumulated literature data. Importantly, this should not be seen as a weakness, but rather as a key strength that is currently underemphasized in the

manuscript. In its present form, the storyline aligns well with a catalysis focused journal, whereas the connection to engineering practice, particularly expected for Communications Engineering, remains less clearly articulated. For example, the motivation in the abstract ([Presently, the optimization of heterogeneous catalysis relies on navigating a complex parameter space through intuition-guided, resource-intensive experimentation]) would benefit from a clearer engineering context. The authors are therefore encouraged to more explicitly position their work within an engineering framework, highlighting its relevance for industrial applications such as identifying improved operating conditions, enhancing process efficiency, or enabling cost reductions in established catalytic systems. In this context, it would be particularly valuable to identify and discuss existing gaps between current industrial practice and data-driven optimization approaches, supported by appropriate references. This perspective should be consistently reflected in the Abstract, Introduction, and Conclusions. While no additional quantitative analysis is required, a clearer articulation of the practical and industrial relevance would significantly strengthen the manuscript and its suitability for the target journal.

Comment 5 (related to Comment 2):
Thank you for the detailed response. Regarding the discussion on data curation, it would be helpful if the authors more explicitly highlight the current limitations of automated data extraction within the manuscript. In particular, a concise discussion of future perspectives, such as the potential role of large language models (LLMs) and automated summarization tools in facilitating literature data extraction, would strengthen the outlook of the study. Recent work in this direction may be briefly cited, for example https://arxiv.org/abs/2403.02901 and https://doi.org/10.1080/00219592.2024.2360928. This addition can remain concise but would provide useful context regarding scalability and future development of the framework.

Open Access This Peer Review File is licensed under a Creative Commons Attribution 4.0 International License, which permits use, sharing, adaptation, distribution and reproduction in any medium or format, as long as you give appropriate credit to the original author(s) and the source, provide a link to the Creative Commons license, and indicate if changes were made.
In cases where reviewers are anonymous, credit should be given to 'Anonymous Referee' and the source.
The images or other third party material in this Peer Review File are included in the article's Creative Commons license, unless indicated otherwise in a credit line to the material. If material is not included in the article's Creative Commons license and your intended use is not permitted by statutory regulation or exceeds the permitted use, you will need to obtain permission directly from the copyright holder.
To view a copy of this license, visit https://creativecommons.org/licenses/by/4.0/

Reviewers' comments:

Reviewer #1:

Comment 1-0:

In this paper Duan, Kim, et al. demonstrated an application of machine learning for tuning experimental parameters rather than using intuition-guided as in traditional method which could be very useful for industrial process in reaction optimization. The data was well curated from 200 papers since 1995 of CO2-to-methanol synthesis using Cu/ZnO-based catalysts and activation energy (Ea). 5 models were trained to predict CO2 conversion efficiency, methanol selectivity, CO selectivity and methanol space-time yield. This paper can be accepted after a major revision.
Here are my comments.

Response:

We sincerely appreciate the reviewer's positive comment. We have performed a major revision to improve the rigor and clarity of data preprocessing, add an additional baseline model (SVM), use pre-training model for promoters, and provide more practitioner-friendly visualizations and optimization guidance. Below we respond to each comment point-by-point. All changes have been incorporated into the revised manuscript and Supporting Information.

Comment 1-1:

In Page 4 Line 111-113, the authors used mean imputation to fill data such as surface area or GHSV, can these values be estimated by a more scientific way? Such as interpolation or using Monte Carlo methods.

Response:

We thank the reviewer for this important suggestion. In the revised workflow, we replaced the previous simple mean filling with a kernel-density-estimation (KDE) based stochastic imputation scheme for the small fraction of remaining descriptor-level missingness. Continuous descriptors, such as catalyst surface area, GHSV, and calcination time, were imputed by sampling from a KDE estimated from the observed values of each descriptor, whereas categorical descriptors were imputed by sampling from their empirical distributions. Promoter-ratio descriptors were treated separately: entries explicitly labeled as "Null" were assigned a ratio of zero, while missing ratios for non-null promoters were imputed from promoter-specific observed distributions when available, with fallback to the global observed ratio pool.

We did not apply interpolation because the missing entries in our database do not arise from ordered experimental sequences, but from independent literature reports collected across different studies. In this sense, the revised imputation is closer to a data-driven stochastic, Monte Carlo-style sampling procedure constrained by the observed

descriptor distributions.

We have revised the relevant description in the main text and Supplementary Note 2 accordingly, and the implementation details are documented in the updated preprocessing code.

Modified and added sentences (Page 5):

"For the remaining dataset, sparse descriptor-level missingness (overall below 5%) was handled by kernel-density-estimation (KDE)-based stochastic imputation for continuous variables and empirical-distribution sampling for categorical variables, thereby preserving the full entry count (*Supplementary Note 2*)."

Modified sentences in Supplementary Information (Supplementary Note 2):

"After excluding records with non-reconstructable key performance outputs, the remaining descriptor-level missingness was sparse and was handled without removing additional entries. Because these missing values originated from different literature reports rather than ordered experimental sequences, interpolation was not applied. Instead, continuous descriptors were imputed using a kernel-density-estimation-based stochastic sampling procedure fitted to the observed values of each descriptor, whereas categorical descriptors were imputed by sampling from their empirical frequency distributions. Promoter-ratio descriptors were treated separately: entries explicitly reported as "Null" were assigned a ratio of zero, while missing ratios associated with non-null promoters were imputed from promoter-specific observed distributions when available, with fallback to the global observed ratio pool. Where necessary, sampled values were constrained to physically meaningful ranges. Full implementation details are provided in the accompanying preprocessing code."

Comment 1-2:

Why the reaction temperature used is in degree Celsius instead of Kelvin, does it yield difference results?

Response:

Thank you for raising this question. Converting °C to K (and vice versa) is a constant shift. Since we standardize numerical features using Z-score normalization, this constant shift does not change the standardized values, and therefore does not change ML predictions or learned relationships. For kinetic analysis (Arrhenius fits), we explicitly use absolute temperature in Kelvin, as required by the Arrhenius equation. We have clarified this point in the revised main text.

Modified and added sentences (Page 4):

"For the ML models, replacing °C by K (or vice versa) corresponds to an affine transformation and, after feature standardization, does not alter the information content

of the temperature descriptor. Absolute temperature in K was used wherever physically required, namely in the Arrhenius-based E_{app} analysis in Section 2.5."

Comment 1-3:

In figure 2b, it is not clear, as the author mentioned that missing data was filled, but "Missing and outlier data" was dropped, why there are still missing data at this point as you have dropped in the previous process, more explanation needed.

Response:

We appreciate the reviewer's point. In Fig. 2b, the step labeled "Missing and outlier data discarded" does not refer to the missing entries of input descriptors that can be imputed. Instead, it refers to datapoints that are unusable because (i) essential performance targets are missing and cannot be reconstructed, or (ii) the reported values are not directly comparable or are physically inconsistent with the reported reaction conditions (outliers).

For the missing performance targets, we attempted to reconstruct missing STY_{MeOH} or X_{CO2} whenever the complementary quantities were available, as follows.

For studies that did not explicitly report the methanol space-time yield (STY_{MeOH}), we calculated it using the reported CO_2 conversion (X_{CO_2}), methanol selectivity (S_{MeOH}), and CO_2 gas hourly space velocity ($GHSV_{CO_2}$):

$$STY(g_{MeOH}\ kg_{cat}^{-1}\ h^{-1}) = \frac{GHSV_{CO_2} \times X_{CO_2} \times S_{MeOH} \times M_{MeOH}}{22.4} \qquad (S1)$$

where $M_{MeOH} = 32.04\ g\ mol^{-1}$ is the molar mass of methanol. When $GHSV_{CO_2}$ was not directly available, it was estimated by multiplying the total GHSV by the molar fraction of CO_2 in the feed gas mixture. This approach enabled consistent derivation of STY values across the dataset, even when not explicitly reported in the original sources.

Conversely, in cases where CO_2 conversion was not reported but STY_{MeOH} and S_{MeOH} were available, we rearranged Equation (S1) to calculate CO_2 conversion:

$$X_{CO_2} = \frac{STY(g_{MeOH}\ kg_{cat}^{-1}\ h^{-1}) \times 22.4}{GHSV_{CO_2} \times S_{MeOH} \times M_{MeOH}} \qquad (S2)$$

This reciprocal approach allowed us to consistently reconstruct missing conversion or productivity values and maximize the usable datapoints in the final dataset.

However, if a publication reported only a single metric (e.g., STY_{MeOH} alone) without the additional information required by Equations. S1–S2 (e.g., selectivity and/or $GHSV_{CO2}$), we could not reconstruct the missing targets in a consistent way, and such entries were excluded.

After the above filtering, the remaining missing values were in input descriptors (e.g., surface area, calcination time, or type of synthesis procedure). These were filled using a

KDE-based Monte Carlo imputation scheme as described in the response to Comment 1-1.

To avoid ambiguity, we have revised the label and caption of Fig. 2b to explicitly state that "missing data discarded" refers to "Non-reconstructable outputs removed", whereas missing values in input descriptors were imputed.

Revised the corresponding text in Section 2.1 (Page 5):

"Some studies reported only partial information, resulting in missing values in either (i) performance outputs (e.g., STY_{MeOH} or X_{CO2} not explicitly reported) or (ii) selected input descriptors (e.g., catalyst surface area or calcination time). For performance outputs, we reconstructed missing STY_{MeOH} or X_{CO2} whenever the complementary metrics were available using Eqs. (S1) and (S2) (*Supplementary Note 1*), while performance entries lacking sufficient information to reconstruct the key performance outputs were excluded (*Supplementary Note 2*), leaving 2476 entries from the 2,551 extracted records (**Fig. 2b**). For the remaining dataset, sparse descriptor-level missingness (overall below 5%) was handled by kernel-density-estimation (KDE)-based stochastic imputation for continuous variables and empirical-distribution sampling for categorical variables, thereby preserving the full entry count (*Supplementary Note 2*)."

Revised the corresponding text in Section 2.1 (Page 5):

"After the initial screening (removal of non-reconstructable records) and the KDE-based imputation of the remaining descriptor missingness, these features were first transformed using a natural logarithm to produce a more Gaussian-like distribution."

Revised Figure 2b and its caption:

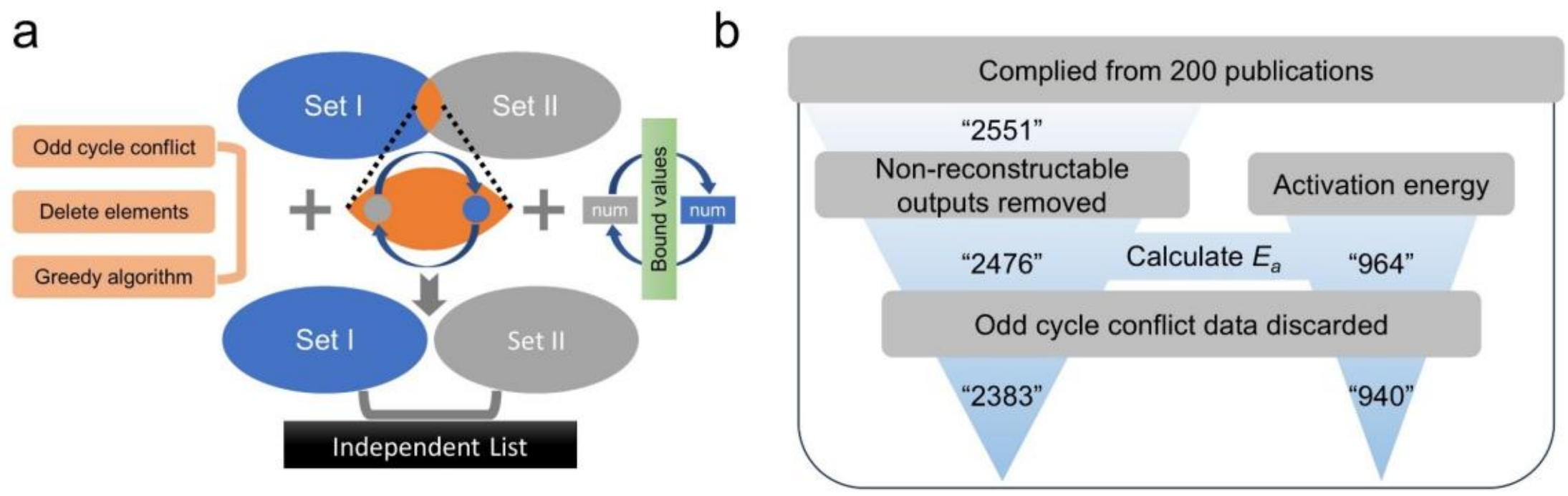

Figure 2. Data curation and symmetry conflict resolution workflow. **(a)** Schematic of the greedy algorithm used to identify and resolve odd-cycle conflicts arising from unordered promoter pairs. By detecting minimal sets of conflicting entries and removing redundancies, the dataset is reduced to a permutation-invariant, independent list suitable for modeling. (b) Dataset reduction flowchart. Of the 2551 initially extracted entries from 200 publications, 2476 remained after excluding records with non-reconstructable performance outputs and non-comparable/erroneous outlier reports

(*Supplementary Note 2*). The remaining descriptor-level missingness was handled by imputation. A final refinement step, removing odd-cycle conflicts, yielded 2383 valid data points used for model training. A similar process was applied to the activation-energy subset.

Comment 1-4:

As you only have around two thousands points, Support Vector Machine (SVM) could provide better accuracy in small dataset (Please see https://www.nature.com/articles/s41467-020-19594-z)

Response:
We thank the reviewer for this valuable suggestion and reference. Support vector methods can be highly competitive for datasets of moderate size. Following this suggestion, we expanded the model benchmark by including Support Vector Machine (SVM) under the same Bayesian hyperparameter optimization, regularization, and 5-fold cross-validation framework used for the other models.

In the revised benchmark, SVM indeed showed competitive predictive performance, confirming that margin-based learning is suitable for the present dataset size. However, CatBoost still provided the best overall trade-off between accuracy and generalizability under the unified evaluation protocol at $\alpha = 0.01$. This likely reflects the structured yet highly heterogeneous nature of the curated catalysis dataset, in which nonlinear feature interactions and regime-dependent effects are more effectively captured by boosted tree ensembles than by a single kernel-based regressor. Therefore, although SVM has now been included in the comparison, CatBoost was retained as the final model for the subsequent SHAP analysis and optimization mapping.

We have added the results of SVM in Figure 4, Table 2, and the corresponding Supporting Information accordingly.

Comment 1-5:

Figure 6 has too much details, it is extremely hard to interpret the results and understand to results. This figure can be rearranged or moved to supporting materials if there is no better way to depict the results.

Response:
Thank you for this helpful comment. In the revised manuscript, we removed the former promoter–synthesis matrix (original Figure 6) from the main article and relocated the corresponding detailed materials-level attribution analysis to the Supporting Information.

In addition, as part of the broader revision associated with Comment 1-8, the promoter representation was updated from one-hot labels to Magpie-based chemistry-informed descriptors (pre-train embedding model). Accordingly, in the revised SHAP attribution results, the top two categorical features are now identified as Promoter 1 and Promoter 2,

as shown in **Figure S20**. This representation provides a more compact and chemically more meaningful summary of promoter effects. To preserve the experimentally recognizable identity-level information, we further included a detailed promoter-pair response map in the Supporting Information (**Figure S21**) and added an expanded explanation of how this map should be interpreted.

Specifically, the revised Supporting Information now clarifies that the promoter-pair map should be read as a qualitative trend atlas under representative fixed background conditions. These changes substantially improve readability in the main text while retaining the chemically useful pairwise promoter information for interested readers.

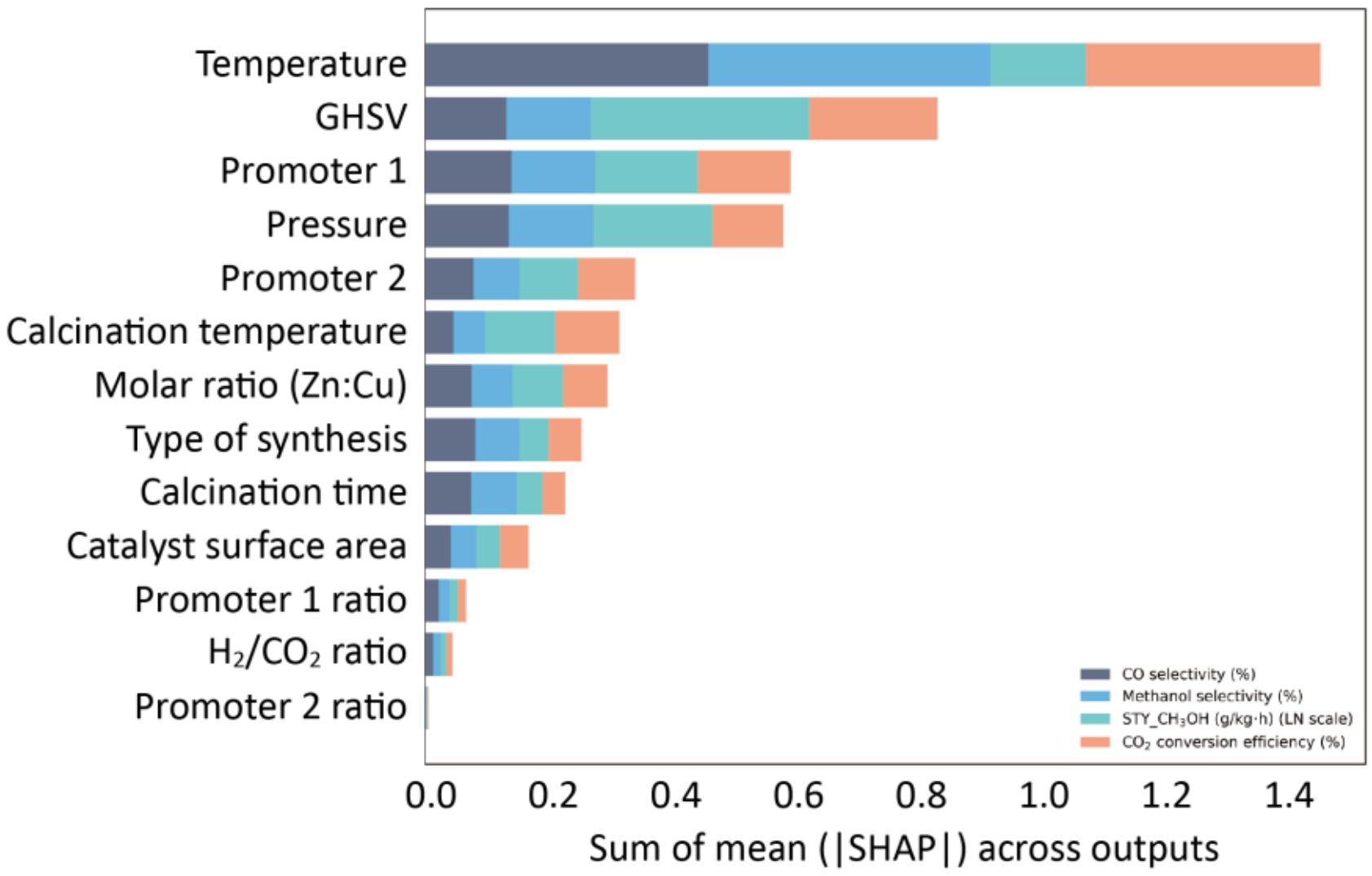

Figure S20. Global feature importance analysis for normalized SHAP values of the trained optimal CatBoost model for the summation of the four output targets.

Modified sentences in Section 2.4 related to promoter-pair map (Page 14):

"Beyond the operating windows, promoter effects can be interpreted at the pair level. This point is especially important because promoter combinations were encoded through chemistry-informed descriptors under a canonical permutation-invariant representation, such that Promoter 1 and Promoter 2 denote two slots in the representation rather than mechanistically distinct primary and secondary roles. Accordingly, promoter effects are visualized separately in the promoter-pair response matrix (**Fig. S21** and *Supplementary Note 5*), which provides composition-level guidance complementary to the operating heatmaps in **Fig. 5**. The matrix further shows that promoter combinations favorable for X_{CO2} or STY_{MeOH} do not necessarily maximize S_{MeOH} or minimize S_{CO}, reinforcing the target-specific nature of catalyst optimization."

Added sentences in Supporting Information (Supplementary Note 5):

"To retain an experimentally recognizable view of promoter effects, we back-projected the trained CatBoost model from the Magpie-based promoter-descriptor space to promoter-identity space. For each promoter identity in the curated library, the

corresponding Magpie descriptor vector was assigned to the Promoter 1 or Promoter 2 slot of the canonical catalyst representation, while all remaining catalyst, synthesis, and reaction descriptors were fixed at representative baseline values. The two promoter slots were systematically enumerated across the promoter library after canonical permutation-invariant assignment, while all non-promoter variables were fixed at the same representative reference setting used for the main-text inference maps. The resulting predictions are summarized in the promoter-pair response map shown in **Fig. S21**.

Each square in **Fig. S21** corresponds to one promoter-identity pair and is partitioned into four triangular sectors reporting S_{CO} (bottom), S_{MeOH} (right), STY_{MeOH} (top, ln scale), and X_{CO2} (left), respectively. The color in each sector denotes the model-predicted response under the same background setting. The figure should therefore be interpreted as a compact multi-output screening atlas. Entries involving "Null" represent single-promoter formulations, whereas the Null–Null entry corresponds to the unpromoted baseline under the same descriptor setting, i.e., Cu/ZnO catalyst.

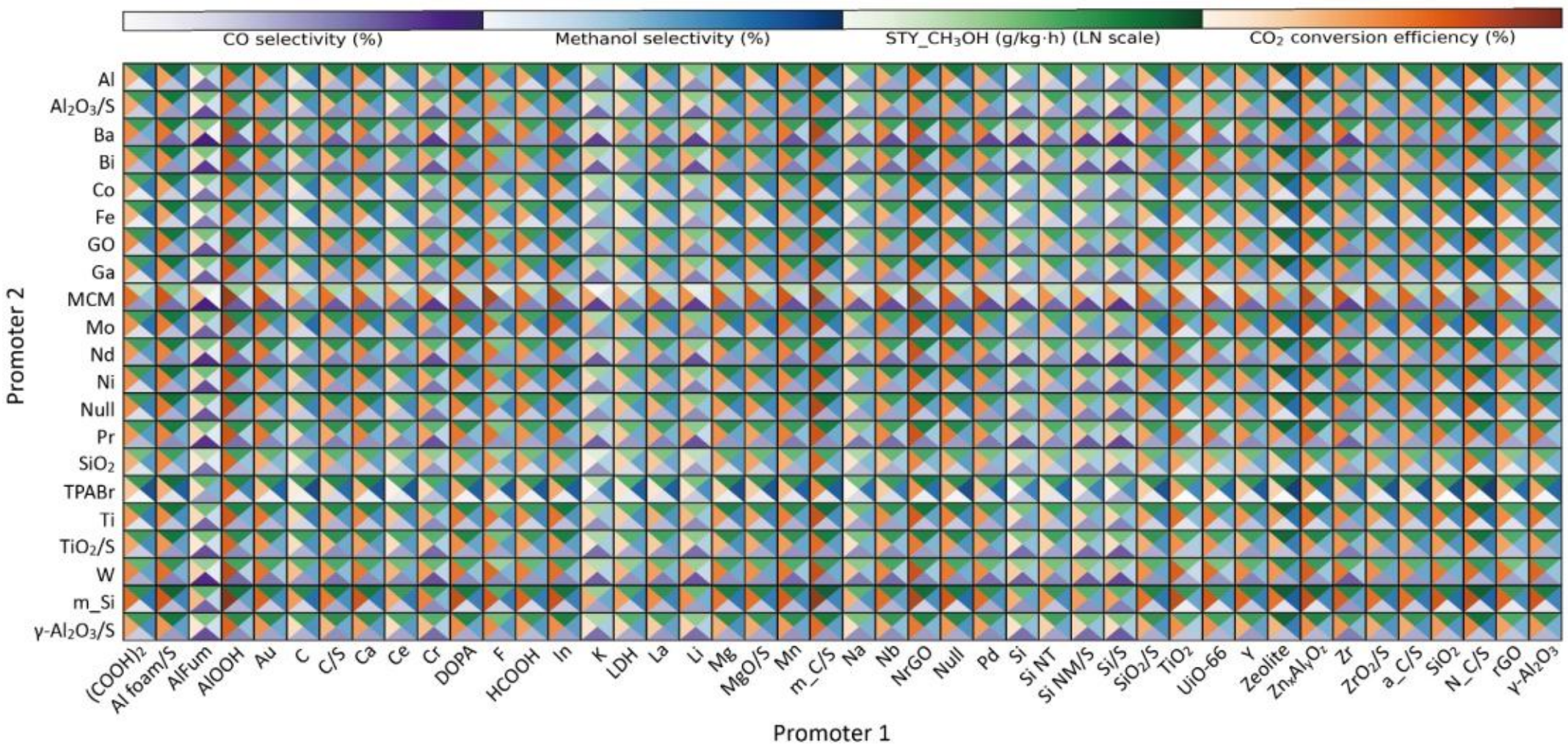

Figure S21. Promoter-pair response matrix predicted by the optimal CatBoost model under the representative reference setting used for the main-text inference analysis. Each square corresponds to one promoter-identity pair in the curated promoter library, with all remaining catalyst, synthesis, and reaction descriptors fixed at representative baseline values. The four triangular sectors report the predicted S_{CO} (bottom), S_{MeOH} (right), STY_{MeOH} (top, ln scale), and X_{CO2} (left), respectively. Promoter 1 and Promoter 2 denote the two slots in the canonical permutation-invariant representation and do not imply intrinsically distinct mechanistic roles.

Abbreviations used in the promoter labels: GO, graphene oxide; MCM, Mobil Composition of Matter (from 10.1016/j.jclepro.2016.07.099); TPABr, tetrapropylammonium bromide; m_Si,

mesoporous Si; a_SiO2, amorphous SiO2; LDH, layered double hydroxide; a_C/S, activated carbon/S; m_C/S, mesocarbon/S; N_C/S, N-doped carbon/S; C/S, carbon/S; C, carbon.

Promoter 1 and Promoter 2 are slot labels inherited from the canonical permutation-invariant representation introduced during preprocessing; they do not imply intrinsically different mechanistic functions, temporal order of addition, or a strict distinction between primary and secondary promoters. Whereas the grouped SHAP analysis of **Fig. S20** identifies which materials-related descriptor groups matter most overall, **Fig. S21** shows how those effects appear when projected onto specific promoter identities.

Because the predicted influence of a promoter pair remains conditional on the fixed background composition, synthesis descriptors, and reaction conditions, **Fig. S21** should be read as a qualitative trend atlas rather than an absolute ranking of optimal catalyst formulations. Consistent with the SHAP and operating-window analyses in the main text, the map further reveals pronounced metric-dependent trade-offs: promoter pairs that favor X_{CO2} or STY_{MeOH} do not necessarily maximize S_{MeOH} or minimize S_{CO}. This observation further underscores the inherently multi-objective nature of promoter selection in Cu/ZnO-based methanol synthesis."

Comment 1-6:

The author should have a demonstration or suggestion of how to maximize/minimize the output for example, with combination of promoter A and promoter B in temperature C can maximize CO2 conversion efficiency, I suggested graphs of a heatmap where x and y are 2 most feature importance and performance metrics as heat points, so the chemists who are not ML/AI based can also understand.

Response:

We thank the reviewer for this insightful suggestion. In the revised manuscript, we now use output-specific pairwise heatmaps based on the 2 most influential descriptors identified by SHAP for each performance metric.

For methanol selectivity, the heatmap is plotted against temperature and GHSV; for methanol space-time yield, against GHSV and pressure; and for CO_2 conversion, against temperature and GHSV. For CO selectivity, the second-ranked SHAP feature is Promoter 1, which is a categorical descriptor rather than a continuous operating variable. Therefore, to preserve the readability of the heatmaps, we used the two highest-ranked continuous descriptors, temperature and pressure, and retained the promoter-pair response analysis in the Supporting Information to provide composition-level guidance.

These revised maps (new **Fig. 5**) now provide direct experimental guidance for maximizing or minimizing each performance metric. Within the literature-derived domain, high temperature and low-pressure favor CO selectivity, low temperature and high GHSV favor

methanol selectivity, high GHSV together with elevated pressure favor methanol productivity, and high temperature with low GHSV favors CO_2 conversion.

Specifically, for example, if CO_2 conversion efficiency is prioritized, the model identifies a high-conversion window at ca. 320–350 °C and ln(GHSV) ca. 7.5–8.5, where the predicted X_{CO2} reaches approximately 22–25%. Within the promoter-pair matrix, combinations such as AlOOH–m_Si, m_C/S–m_Si, AlOOH–MCM, and m_C/S–MCM fall into the most favorable high-X_{CO2} regions. In contrast, if methanol selectivity is prioritized, the preferred operating window shifts to low temperature and high GHSV, namely ca. 150–180 °C and ln(GHSV) ca. 9.5–11.0, where the predicted methanol selectivity approaches 90–100%. In the promoter-pair matrix, the most favorable S_{MeOH} regions are associated with TPABr-containing pairs, especially TPABr–rGO, TPABr–Zeolite, TPABr–N_C/S, and TPABr–C. These examples were added to make the ML-derived guidance more directly actionable for chemists.

We also revised the main text and figure caption accordingly to make the model-derived trends more accessible to chemists without a ML background.

<u>Modified sentences in Section 2.4 (Pages 12 and 14):</u>

"**Figure S20** summarizes the aggregate SHAP importance across the four outputs, whereas the target-specific rankings in **Figs. 5a–d** show that the dominant descriptor pair depends on the selected metric. Temperature remains the leading operational descriptor for S_{CO}, S_{MeOH}, and X_{CO2}, GHSV dominates STY_{MeOH}, pressure is a recurrent secondary operating lever, and promoter-related descriptors emerge as major composition-level contributors."

"To convert the SHAP analysis into experimentally actionable guidance, we generated output-specific pairwise inference maps using the most influential continuously varying operating descriptors for each target while fixing the remaining variables at representative reference values. Accordingly, S_{CO} is mapped against temperature and pressure because its second-ranked contribution arises from promoter-related descriptors, whereas S_{MeOH} and X_{CO2} are mapped against temperature and ln(GHSV), and STY_{MeOH} is mapped against pressure and ln(GHSV). The 2D heatmaps directly indicate how each performance metric can be maximized or suppressed within the literature-covered domain. S_{CO} increases strongly with temperature and is highest at relatively low pressure, consistent with the increasing prevalence of RWGS-favored conditions. In contrast, S_{MeOH} is maximized at low temperature and high GHSV, revealing an operating window that suppresses CO formation while preserving methanol selectivity. STY_{MeOH} follows a different optimization logic, which rises steeply with GHSV and additionally benefits from elevated pressure, although a broad productivity plateau emerges once the flow is sufficiently high. X_{CO2} is maximized at high temperature and low GHSV, reflecting the combined importance of thermal activation and residence time. Taken together, these maps show that there is no universal optimum for all outputs; instead, the preferred operating window shifts according to whether CO suppression, methanol selectivity, methanol productivity, or overall CO_2 conversion is prioritized. Notably, the STY_{MeOH} map indicates that once ln(GHSV) becomes sufficiently high, industrially relevant methanol productivity can already be approached at substantially milder pressures than conventionally assumed. "

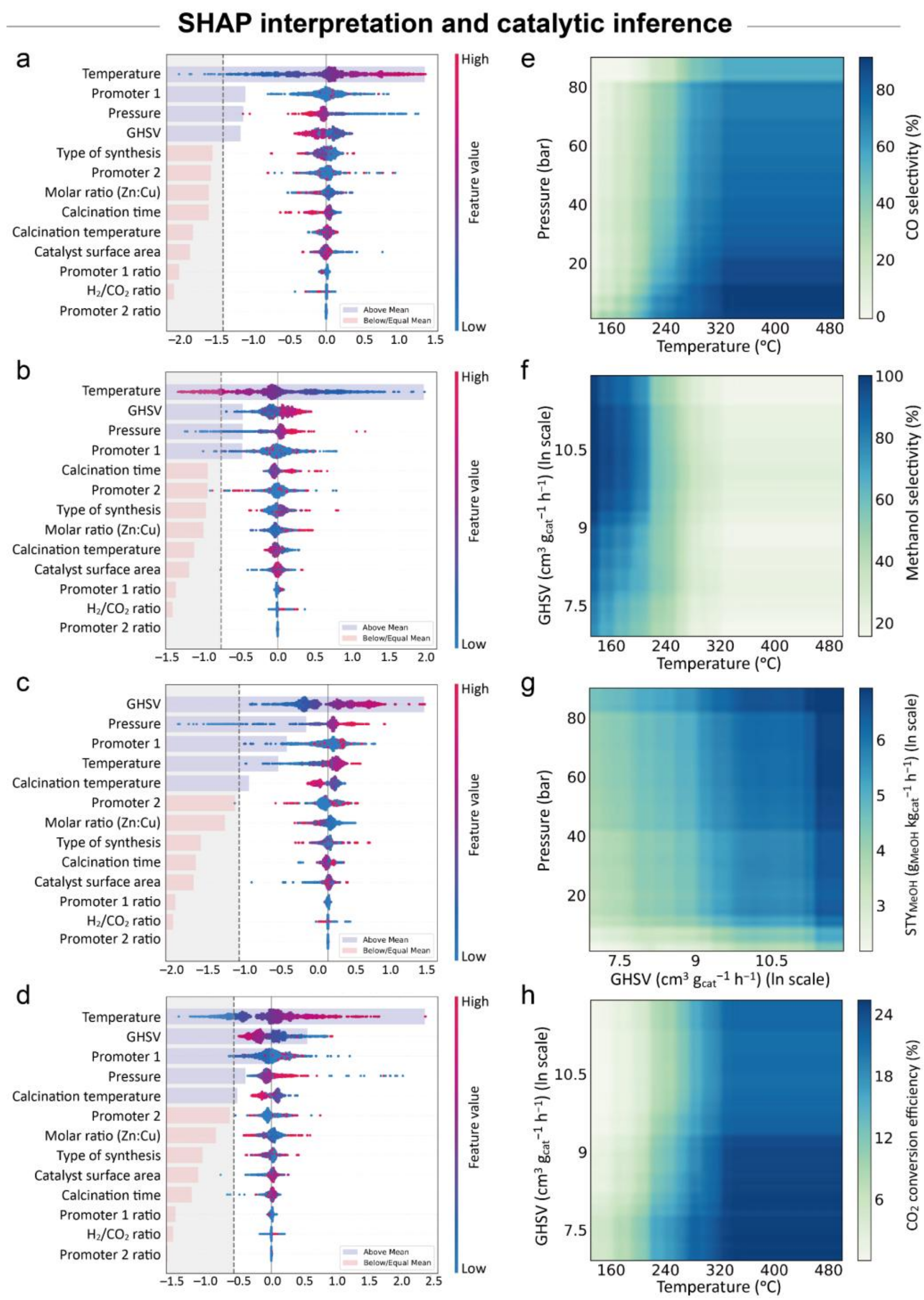

Figure 5. Translating ML insights to catalytic chemical space. (a–d) SHAP summary plots of the optimal CatBoost model, showing feature importance and directional impact for (a) S_{CO}, (b) S_{MeOH}, (c) STY_{MeOH} (ln scale), and (d) X_{CO2}. Each dot represents one literature data point; color denotes the relative feature value, and the horizontal position indicates whether that feature value contributes positively or negatively to the predicted output. (e–h) SHAP-guided pairwise inference heatmaps constructed from the most influential continuously varying operating descriptors for each target. Accordingly, (e) S_{CO} is mapped against temperature and pressure, (f) S_{MeOH} against temperature and ln(GHSV), (g) STY_{MeOH} against pressure and ln(GHSV), and (h) X_{CO2} against temperature and ln(GHSV). Promoter-combination effects are resolved separately in **Figure S21**.

"The maps (**Figs. 5 and S21**) allow concrete optimization suggestions to be formulated. For instance, when CO_2 conversion efficiency is prioritized, the model points to a high-temperature, low-GHSV regime, with a representative high-conversion window at ca. 320–350 °C and ln(GHSV) ca. 7.5–8.5. Within the promoter-pair matrix, combinations such as AlOOH–m_Si, m_C/S–m_Si, AlOOH–MCM, and m_C/S–MCM fall into the most favorable X_{CO2} regions. By contrast, maximizing methanol selectivity requires the opposite operating regime, namely low temperature and high GHSV. A representative high-selectivity window is ca. 150–180 °C and ln(GHSV) ca. 9.5–11.0, while the most favorable promoter-pair regions are associated with TPABr-containing pairs such as TPABr–rGO, TPABr–Zeolite, TPABr–N_C/S, and TPABr–C. These examples highlight that catalyst optimization is strongly target-specific and that the preferred windows for conversion and methanol selectivity are fundamentally distinct."

Comment 1-7:

Fix the typo in Figure 7a, it should be Methanol-based instead of Methonal-based.

Response:

Thank you for pointing out this error. We apologize for the oversight. The typo has been corrected to "Methanol-based" in the revised manuscript.

Comment 1-8:

The author can try using pre-train embedding model (such as Chemberta) instead of one-hot encoding, this can enhance model's performance as the embedding vector already contains chemical properties.

Response:

We thank the reviewer for this insightful suggestion. We revised the encoding of Promoter 1 and Promoter 2 from one-hot encoding to a dense chemistry-informed representation based on Magpie descriptors, and we retrained the full benchmark accordingly. Each promoter was represented by a Magpie-derived descriptor vector and coupled with its corresponding promoter/Cu molar ratio. This way can indeed enhance the physical meaning of the ML models, and in the SHAP analysis, the feature importance of promoter increased. We thus did Promoter-pair response matrix, as shown in Fig. S21.

We selected Magpie rather than ChemBERTa because our promoter field is not a uniform set of canonical molecular SMILES, but a heterogeneous materials space containing inorganic elements, oxides, mixed compositions, and support-like labels reported in the literature. In this setting, a composition-based materials descriptor is more appropriate and also better aligned with the interpretability objective of the present study. The revised model performance and the corresponding updated figures and tables are now reported in the revised manuscript and Supporting Information.

Added sentences in Section 2.1 (Page 5):

"Promoter identities were represented by Magpie-derived (Magpie, a composition-based feature engineering framework, well suited to small-to-medium materials datasets that require interpretable descriptors for conventional ML) chemistry-informed descriptors, whereas the type of synthesis procedure was retained as a one-hot-encoded process category."

New Table 2 (Page 3):

Table 2. Validation and cross-validated R^2 and overfitting indices for six ML models at different regularization strengths (α)

Model	α	R^2 (Valid)				Overfitting		CV (R^2)
		S_{CO}	S_{MeOH}	STY_{MeOH}	X_{CO2}	MSE Ratio†	R^2 difference ‡	
RF	**0**	**0.749**	**0.775**	**0.752**	**0.679**	**3.85**	**0.19**	**0.75**
	0.01	0.749	0.775	0.752	0.679	3.82	0.19	0.75
	0.03	0.738	0.756	0.733	0.662	2.81	0.17	0.73
	0.07	0.706	0.723	0.716	0.641	2.12	0.15	0.69
CatBoost	0	0.867	0.887	0.858	0.755	21.02	0.15	0.85
	**0.01**	**0.843**	**0.864**	**0.832**	**0.737**	**4.14**	**0.13**	**0.83**
	0.03	0.808	0.834	0.818	0.719	2.53	0.12	0.80
	0.07	0.786	0.812	0.806	0.707	2.11	0.11	0.78
DT	0	0.624	0.670	0.631	0.564	4.32	0.29	0.58
	**0.01**	**0.620**	**0.667**	**0.629**	**0.559**	**3.81**	**0.28**	**0.58**
	0.03	0.555	0.603	0.629	0.550	2.21	0.22	0.54
	0.07	0.545	0.601	0.570	0.492	1.54	0.14	0.51
ANN	**0**	**0.655**	**0.647**	**0.675**	**0.632**	**1.60**	**0.12**	**0.67**
	0.01	0.605	0.633	0.681	0.629	1.38	0.08	0.66
	0.03	0.629	0.650	0.639	0.592	1.50	0.11	0.68
	0.07	0.645	0.660	0.693	0.643	1.54	0.10	0.66
XGB	0	0.854	0.876	0.867	0.760	56.75	0.16	0.85
	**0.01**	**0.822**	**0.840**	**0.833**	**0.743**	**4.56**	**0.14**	**0.82**
	0.03	0.782	0.808	0.813	0.725	2.76	0.13	0.79
	0.07	0.751	0.778	0.782	0.696	2.09	0.12	0.77
SVM	**0**	**0.762**	**0.779**	**0.777**	**0.663**	**4.03**	**0.18**	**0.76**
	0.01	0.751	0.779	0.773	0.656	3.28	0.17	0.75
	0.03	0.744	0.769	0.766	0.659	2.98	0.17	0.75
	0.07	0.694	0.717	0.729	0.656	2.00	0.14	0.71

†MSE Ratio = mean(MSE_{valid}/MSE_{train}) across S_{CO}, S_{MeOH}, STY_{MeOH}, and X_{CO2}; ‡R^2 difference = mean($R^2_{train} - R^2_{valid}$) across the same four targets.

Comment 1-9:

The author should publish the code used in this paper.

Response:

Thank you. Our reproducible code and dataset are available at https://github.com/MoYan-101/CommsEng.

Reviewer #2:

Comment 2-0:

This study integrates 2,476 experimental data points from 200 literature sources between 1995 and 2025, establishing a machine learning dataset that covers catalyst composition, synthesis methods, reaction conditions, and performance indicators. By employing Bayesian optimization, regularization strategies, and SHAP interpretive analysis, it reveals the nonlinear relationships in methanol synthesis. However, the following aspects need to be further revised and improved.

Response:

Thank you very much for the constructive comments. In the revised manuscript, we (i) expanded the discussion on the novelty of our work by positioning it against existing ML-assisted CO_2-to-methanol studies; (ii) added discussion about limitations of publication bias, missing/derived variables, and uncertainty introduced by literature mining; (iii) revised SHAP-related statements; and (iv) did experimental verification.

Comment 2-1:

There are already many studies on the application of machine learning to accelerate the optimization design of catalysts for CO2-to-methanol. The uniqueness of this research needs to be described and discussed in detail.

Response:

Thank you for this important comment. We have therefore revised the manuscript to position our contribution more precisely relative to prior work.

Existing studies relevant to CO_2-to-methanol catalysis include: (i) DFT- or adsorption-energy-based ML screening of alloy catalysts; (ii) literature-based prediction of single or limited performance metrics across multiple catalyst families; (iii) process-oriented ML studies coupled with optimization, techno-economic analysis, and life-cycle assessment; and (iv) closed-loop Bayesian optimization based on newly generated high-throughput experimental catalyst libraries. However, no prior study has focused specifically on Cu/ZnO, the catalyst family with the deepest historical foundation and the most established benchmark status in methanol synthesis. This gap also motivates the present work. One of the key contributions of this study is the organization of this body of experimental knowledge into a machine-learning-ready framework.

The present study therefore lies in constructing a Cu/ZnO-focused, hand-curated experimental literature framework spanning 1995–2025, which simultaneously integrates catalyst composition, synthesis descriptors, reaction conditions, and four catalytic outputs (X_{CO2}, S_{MeOH}, S_{CO}, and STY_{MeOH}), and further extends the analysis to a literature-derived apparent activation energy subset for kinetic interpretation. To our knowledge, prior ML studies in this area have not combined these dimensions within a single benchmark Cu/ZnO-centered framework.

We further clarify that expert data curation itself constitutes a major contribution of this work, because the relevant experimental literature is highly fragmented, non-standardized, and often requires output reconstruction, unit harmonization, and internal consistency screening before ML analysis becomes meaningful.

To make this distinction explicit, we have added a new literature-positioning paragraph in the Introduction and a new comparative table in the Supporting Information (**Table S5**), where the present work is positioned against representative prior ML studies relevant to CO_2-to-methanol catalysis. We have also revised the novelty statements throughout the manuscript to emphasize that the principal advance lies not in applying ML per se, but in transforming fragmented Cu/ZnO experimental literature into an interpretable literature-to-knowledge framework.

Table S3 Positioning of the present work relative to representative machine-learning studies relevant to CO_2-to-methanol catalysis

**Representative study**	**Data basis**	**Catalyst scope**	**Dataset size**	**Model inputs**	**Model outputs**	**Difference**
Bhardwaj et al. (2023)[1]	Experimental literature	Cu-based catalysts in direct CO_2 hydrogenation	227	Reaction conditions + selected catalyst textural descriptors	X_{CO2}, S_{MeOH}, S_{CO}, STY_{MeOH}	Closest in chemistry, but substantially smaller dataset and no explicit synthesis-history curation or literature-derived kinetic subset
Suvarna et al. (2022)[2]	Experimental literature	Cu-, Pd-, In2O3-, and ZnO-ZrO2-based catalysts	1425 compiled / 1201 modeled	Composition + synthesis conditions + reaction conditions	STY_{MeOH}	Broad cross-family framework centered on a single target; not Cu/ZnO-benchmark-focused and no kinetic extension
Vanjari et al. (2023)[3]	Experimental literature	Diverse catalysts for direct CO_2 hydrogenation to methanol	698	Catalyst composition + preparation conditions + operating parameters	X_{CO2}, S_{MeOH}	Multi-catalyst framework with two outputs only; no joint S_{CO}/STY_{MeOH} analysis and no kinetic extension
Ghafarian Nia et al. (2025)[4]	Experimental literature	Catalytic hydrogenation to methanol over diverse catalysts	1765	Operating parameters + catalyst loading + surface area + XRD-derived crystal size/crystallinity	X_{CO2}, S_{MeOH}, S_{CO}	Broader process-oriented framework including optimization, techno-economic analysis, and LCA; not centered on the Cu/ZnO benchmark family and no literature-derived

						kinetic subset
Roy et al. (2021)[5]	DFT + ML high-throughput screening	Cu/Co/Ni/Zn/Mg alloy-based periodic (111) surfaces and HEA systems	Computational screening space, not a literature experimental dataset	Surface microstructure + adsorption energetics	Adsorption energies of intermediates	Computational catalyst screening rather than experimental literature integration
**Present work**	Experimental literature	Cu/ZnO-based catalysts	2551 extracted/ 2383 modeled + 940 E_{app} subset	Composition + synthesis history + operating conditions; symmetry-aware curation	X_{CO2}, S_{MeOH}, S_{CO}, STY_{MeOH} + E_{app} extension	Benchmark-family-focused literature-to-knowledge framework integrating composition, synthesis, operation, multi-output performance, and condition-dependent apparent kinetics

Reference:

1. Bhardwaj, A., Ahluwalia, A. S., Pant, K. K. & Upadhyayula, S. A principal component analysis assisted machine learning modeling and validation of methanol formation over Cu-based catalysts in direct CO2 hydrogenation. *Sep. Purif. Technol.* **324**, 124576 (2023).
2. Suvarna, M., Araújo, T. P. & Pérez-Ramírez, J. A generalized machine learning framework to predict the space-time yield of methanol from thermocatalytic CO2 hydrogenation. *Appl. Catal. B* **315**, (2022).
3. Vanjari, P., Kamesh, R. & Rani, K. Y. Machine learning models representing catalytic activity for direct catalytic CO2 hydrogenation to methanol. *Mater. Today Proc.* **72**, 524–532 (2023).
4. Ghafarian Nia, S. A. *et al.* Machine learning-driven optimization for sustainable CO2-to-methanol conversion through catalytic hydrogenation. *Energy Convers. Manag.* **325**, 119373 (2025).
5. Roy, D., Mandal, S. C. & Pathak, B. Machine Learning Assisted Exploration of High Entropy Alloy-Based Catalysts for Selective CO2 Reduction to Methanol. *Journal of Physical Chemistry Letters* **13**, 5991–6002 (2022).

Added sentences (Introduction, Page 2):

"Although ML has already been applied to CO_2-to-methanol catalysis, existing studies address substantially different problem definitions and data regimes. Some works rely on DFT-assisted or adsorption-energy-based screening to identify hypothetical alloy catalysts.[33] Other literature-based studies predict single or limited performance targets across broader catalyst families, such as methanol space-time yield or conversion and selectivity metrics.[34,35] More recent process-oriented studies further extend ML toward

optimization, techno-economic analysis, and life-cycle assessment, while closed-loop Bayesian-optimization studies accelerate catalyst discovery through newly generated high-throughput experimental libraries.[36] These advances are important, yet they do not provide an experimentally grounded, Cu/ZnO-centered (the catalyst family with the deepest historical foundation and the most established benchmark status in methanol synthesis) framework that simultaneously resolves catalyst composition, synthesis history, operating conditions, multiple catalytic outputs, and regime-sensitive apparent kinetics within one benchmark catalyst family. A detailed comparison with representative prior ML studies is provided in **Table S5**."

Added sentences (Section 2.1, Page 3):

"Importantly, this curation was not merely clerical, as key experimental quantities were often scattered across figures, supplementary tables, and source-specific reporting formats, requiring expert judgment for unit harmonization, output reconstruction, and consistency screening before model training."

Comment 2-2 and 2-3:

In the discussion section, add an analysis of the limitations of the data, for instance, explain that the literature data might have omitted failed experiments or extreme conditions. Additionally, it is suggested to supplement the estimation methods for unreported variables (such as particle size) or future work directions, such as combining characterization data.

Response:

We appreciate the reviewer's insightful comment (the comment 2-2 and 2-3 are the same so we combine them). We have now added a dedicated discussion of the limitations of the literature-derived dataset in the discussion section, explicitly noting the likely underrepresentation of failed experiments and extreme operating conditions, and clarifying that the model should be interpreted within its literature-defined domain of applicability. We further clarified in Section 2.1 and Supplementary Note 2 that how directly reconstructable performance variables were recovered. Finally, in the discussion section, we state that structure-sensitive descriptors such as particle size were excluded from the primary feature space because they were reported too sparsely and through non-equivalent characterization protocols and highlight the integration of characterization-rich descriptors and operando data as a key next step.

Added sentences (Discussion, Page 18):

"At the same time, the present framework has clear boundaries that should be stated explicitly. First, the models necessarily inherit the strengths and weaknesses of the literature from which they are built. Uneven coverage of catalyst formulations and operating windows, inconsistent reporting standards, publication bias, and the scarcity of explicitly negative results all shape the learned response surface. Second, although the curated dataset captures a broad range of catalyst, synthesis, and reaction descriptors, several mechanistically decisive variables remain absent or only weakly represented, including oxidation-state distributions, Cu particle morphology, interface density, and specific Cu–$Zn^{\delta+}$ motifs under reaction conditions. Third, the SHAP relationships reported

here are associative rather than causal. Their value lies in converting a high-dimensional experimental record into chemically intelligible hypotheses, but they should not be overinterpreted as direct mechanistic proof. The same caution applies to literature-derived E_{app} values, which reflect operating-window-dependent observed kinetics.
These limitations also point directly to the next stage of development. The predictive and explanatory power of literature-driven ML should improve as standardized reporting, digitized supporting information, and machine-readable kinetic datasets become more common. Incorporating richer descriptors from operando spectroscopy, microscopy, and physics-informed surrogate variables should help narrow the gap between experimentally reported inputs and the true catalyst state under reaction conditions. More broadly, coupling interpretable ML with targeted closed-loop experimentation and emerging reaction-engineering strategies may provide a more efficient route to catalyst discovery than either retrospective data mining or isolated experiments alone. Viewed in this way, the value of the present work lies not only in the specific maps obtained for Cu/ZnO-based methanol synthesis, but in demonstrating how decades of fragmented experimental effort can be reassembled into forward-looking design principles."

Comment 2-4:

Although SHAP analysis reveals the importance of variables, some of its conclusions (such as the relationship between GHSV and Ea) lack experimental verification and may overly rely on correlation.

Response:
We appreciate the reviewer's concern. We fully agree that SHAP is a post-hoc interpretability tool and, by itself, reveals associations present in the literature-derived dataset rather than causality. In the revised manuscript, we have therefore explicitly clarified this point and re-framed SHAP-derived relationships, including the GHSV–E_{app} trend, as testable hypotheses rather than causal proof. We also softened the mechanistic wording in the discussion of Section 2.5 accordingly.

More importantly, to address the reviewer's concern directly, we added a new experimental verification section together with detailed Supporting Information. Specifically, we performed controlled fixed-bed CO_2 hydrogenation experiments at 1 bar over a commercial $Cu/ZnO/Al_2O_3$ catalyst while varying only the space velocity. Under otherwise identical catalyst, feed, and activation conditions, the experimentally determined methanol-based apparent activation energy increased from 57.0 ± 4.5 kJ mol^{-1} at 750 $cm^3\ g_{cat}^{-1}\ h^{-1}$ to 76.6 ± 13.2 and 93.9 ± 6.8 kJ mol^{-1} at 1500 and 3000 $cm^3\ g_{cat}^{-1}\ h^{-1}$, respectively. The details will be explained in the response of the next Comment 2-5. This new result directly corroborates the positive GHSV–E_{app} relationship suggested by the ML analysis, while avoiding over-interpretation of SHAP as a causal demonstration.

<u>**Added sentences for SHAP analysis (Section 2.4, Page 12):**</u>
"Importantly, SHAP is a post-hoc explanation of a data-trained model and thus captures associations present in the compiled literature dataset rather than proving causality.

Accordingly, we used SHAP primarily as a hypothesis-generating tool and corroborate mechanistic interpretations with independent physicochemical evidence and targeted validation experiments, in line with recent guidance that interpretable ML insights in catalysis should be experimentally and mechanistically validated to avoid spurious correlations. Accordingly, SHAP-derived relationships between operating variables and E_{app} (e.g., GHSV) in the section 2.5 are treated as testable hypotheses inferred from the literature dataset."

Comment 2-5:

The application of the developed machine learning model still needs to be enhanced. Additionally, could experimental verification be introduced?

Response:

We appreciate the reviewer's valuable suggestion. To strengthen the practical application of the developed ML framework, we revised the manuscript in two complementary ways.

First, we now emphasize more clearly that the framework is not only a retrospective tool for interpreting literature data, but also a prospective tool for prioritizing informative experiments. In particular, the model identifies the dominant operational descriptors governing both catalytic performance and apparent kinetics, thereby narrowing the experimental search space and enabling targeted follow-up studies instead of exhaustive trial-and-error optimization.

Second, guided by the ML-derived E_{app} analysis, we introduced an experimental validation using a commercial $Cu/ZnO/Al_2O_3$ catalyst in a controlled fixed-bed reactor. In this validation, only the space velocity was varied, while the catalyst identity, feed composition, and activation procedure were kept constant. The resulting methanol-based apparent activation energy increased systematically with GHSV, from 57.0 ± 4.5 to 76.6 ± 13.2 and 93.9 ± 6.8 kJ mol^{-1} as GHSV increased from 750 to 1500 and 3000 cm^3 g_{cat}^{-1} h^{-1}, respectively. This experiment demonstrates that the ML framework can generate experimentally testable kinetic hypotheses and guide compact yet discriminating validation experiments.

We have revised the Abstract, added the new Section 2.6, the Discussion, and Supplementary Note 6 to reflect this strengthened application-oriented workflow. In this way, the revised manuscript now shows that the framework can serve not only as an interpretable meta-analysis of historical data, but also as a practical decision-support tool for catalyst testing and operating-window selection.

Added experimental validation in the main text:

"To directly test the ML-derived hypothesis that GHSV modulates the observed kinetic barrier for methanol formation, we performed controlled fixed-bed CO_2 hydrogenation

experiments over a commercial $Cu/ZnO/Al_2O_3$ catalyst at 1 bar while varying only the flow rate. The catalyst identity, feed composition, and activation protocol were held constant, and methanol productivity was measured between 160 and 240 °C at GHSV values of 750, 1500, and 3000 $cm^3\ g_{cat}^{-1}\ h^{-1}$. Detailed experimental procedures, Arrhenius fitting, and mechanistic discussion are provided in *Supplementary Note 6*.

Apparent activation energies were extracted from the quasi-linear Arrhenius regions by fitting ln(STYMeOH) against 1000/T (**Fig. 7**). The experimentally determined methanol-based E_{app} increased monotonically with GHSV, from 57.0 ± 4.5 kJ mol^{-1} at 750 $cm^3\ g_{cat}^{-1}\ h^{-1}$ to 76.6 ± 13.2 kJ mol^{-1} at 1500 $cm^3\ g_{cat}^{-1}\ h^{-1}$ and 93.9 ± 6.8 kJ mol^{-1} at 3000 $cm^3\ g_{cat}^{-1}\ h^{-1}$ (**Figs. 7 and S22**). Because only the flow rate was changed while the catalyst, pressure, and feed ratio were fixed, this experiment isolates GHSV as an operational variable and directly corroborates the positive GHSV–E_{app} relationship inferred from the literature-trained ML model. Such behavior is consistent with prior heterogeneous catalysis studies showing that apparent activation energies can remain insensitive to, or become strongly modulated by, flow and residence-time changes depending on the prevailing kinetic regime and surface-coverage state.[114–117]

Direct literature on a GHSV-dependent methanol-based apparent activation energy in CO_2-to-methanol synthesis remains limited. Under a higher-pressure operating window, Bukhtiyarova and co-workers reported nearly unchanged methanol E_{app} values over a commercial $Cu/ZnO/Al_2O_3$ catalyst across the investigated space-velocity range, indicating that space velocity did not perturb the controlling regime under those conditions.[118] By contrast, in other heterogeneous catalytic systems, residence time or space velocity has been shown to influence measured apparent activation energies and regime transitions.[114,115] These precedents support interpreting the present result as a flow-induced change in the observed kinetic regime.

For Cu-based CO_2 hydrogenation specifically, recent mechanistic studies indicate that the apparent kinetics of methanol formation are highly sensitive to the local abundance of adsorbed hydrogen and water. [119] Methanol formation has been linked to methoxy hydrolysis, and water can facilitate the CH_3O^* to CH_3OH step. [120] Accordingly, one plausible interpretation of the present data is that lowering the space velocity promotes a more water-rich and coverage-modified surface microenvironment, thereby lowering the observed Arrhenius slope for methanol formation. However, excessive space velocity shortens residence time, limiting hydrogen availability and disrupting the Cu^0–$Zn^{\delta+}$ interface, thus shifting the system toward higher-barrier pathways (e.g., carboxyl and direct CO_2 dissociation).[44] Thus, maintaining GHSV is essential to preserve low-barrier reactivity and avoid kinetic masking under transport-limited regimes. This interpretation is proposed at the level of observed kinetics and does not imply that the intrinsic elementary activation barrier of the catalyst itself is directly altered by flow rate.

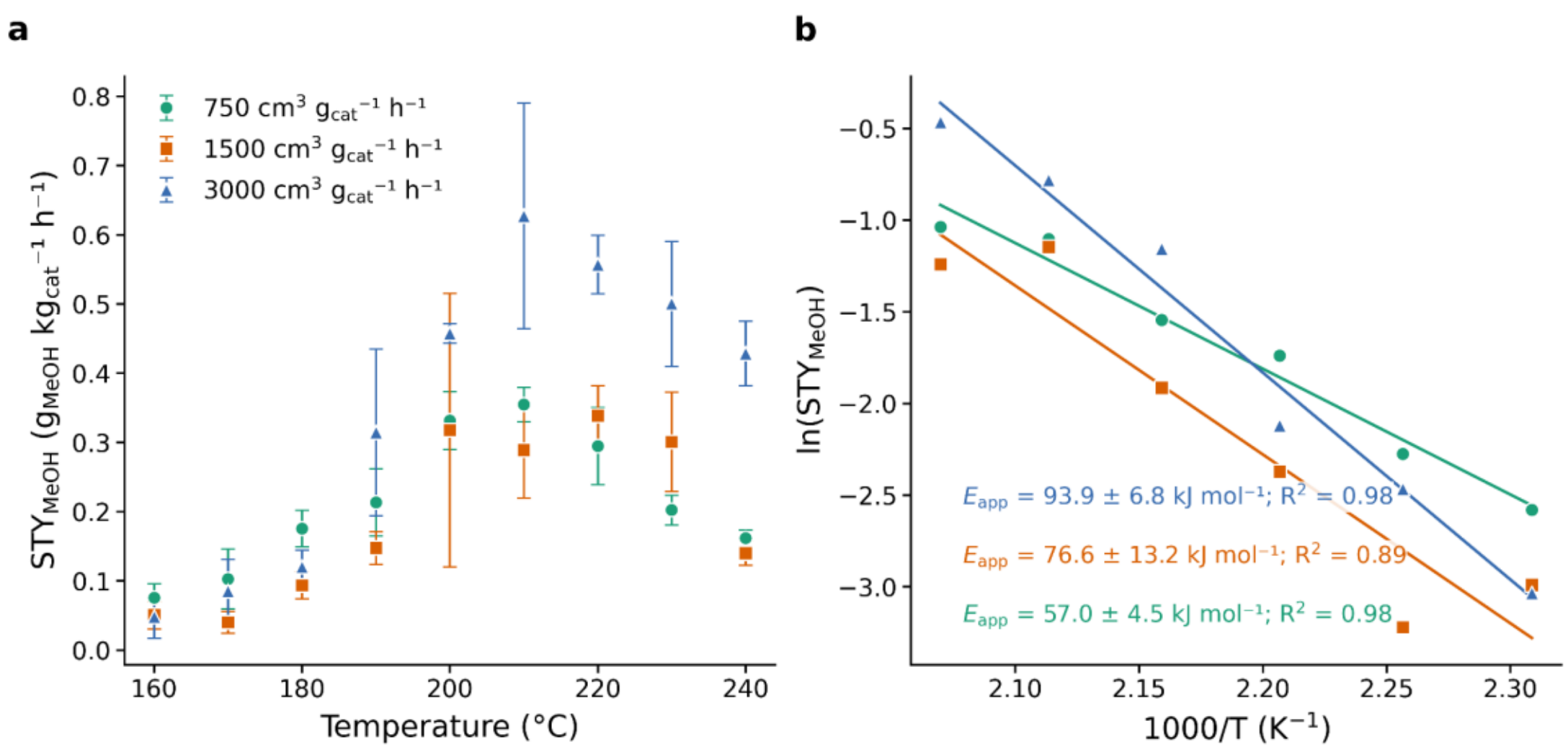

Figure 7. Experimental validation of the ML-derived GHSV–E_{app} relationship over a commercial $Cu/ZnO/Al_2O_3$ catalyst at 1 bar. **a,** Methanol space-time yield, STY_{MeOH}, as a function of reaction temperature at space velocities of 750, 1500, and 3000 cm^3 g_{cat}^{-1} h^{-1}. Error bars represent the standard deviation of repeated measurements. **b,** Arrhenius plots of ln(STY_{MeOH}) versus 1000/T with linear fits applied to the quasi-linear temperature windows used for E_{app} extraction. The fitting windows were 160 to 210 °C. The resulting methanol-based apparent activation energies are 57.0 ± 4.5, 76.6 ± 13.2, and 93.9 ± 6.8 kJ mol^{-1}, respectively."

Supplementary Note 6. Experimental test of the ML-derived GHSV–E_{app} relationship

To directly evaluate the positive GHSV-E_{app} relationship suggested by the ML analysis, we performed a controlled fixed-bed validation experiment over a commercial $Cu/ZnO/Al_2O_3$ catalyst under low-pressure CO_2 hydrogenation conditions. T This experiment was designed to assess the effect of space velocity under otherwise identical catalyst identity, feed composition, and activation conditions. The term GHSV is retained here for consistency with the literature-derived ML dataset, although the present values are reported as mass-normalized volumetric flow rates in cm^3 g_{cat}^{-1} h^{-1}. Notably, a previous study on commercial $Cu/ZnO/Al_2O_3$ at 30 bar reported methanol-based apparent activation energies of 43–44 kJ mol^{-1} and concluded that space velocity did not influence the activation barrier under that higher-pressure operating window.[1] The present low-pressure experiment therefore probes a different kinetic regime.

Materials and fixed-bed reactor setup

A commercial copper-based methanol synthesis catalyst (denoted as CZA) was purchased from Alfa Aesar (product No. 45776; lot No. I06Z036). The catalyst contains 63.5 wt% CuO, 24.7 wt% ZnO, 10.1 wt% Al_2O_3, and 1.3 wt% MgO (nominal composition from the supplier). High-purity gases were used for all experiments: CO_2, H_2, and N_2 (purity ≥ 99.99%).

Catalytic tests were conducted in a continuous-flow fixed-bed reactor operated at 1 bar. About 4.0 g of CZA catalyst was packed in the tubular reactor and fixed between quartz-wool plugs. The reactor temperature was controlled by an electric furnace, and the bed

temperature was monitored by a thermocouple placed close to the catalyst bed center. Gas flow rates were regulated using mass flow controllers.

Catalyst activation and performance tests

Prior to performance measurements, the catalyst was reduced and activated in situ in a H_2 stream at a total flow of 50 cm^3 min^{-1}. The temperature was raised to 350 °C and maintained for 5 h. After activation, the reactor was cooled to 160 °C and purged with N_2 for 30 min to remove residual H_2.

After stabilization, the CO_2/H_2 (1:3) feed was introduced at space velocities of 750, 1500, and 3000 cm^3 g_{cat}^{-1} h^{-1}, respectively. Catalytic performance was evaluated from 160 to 240 °C by increasing the temperature in 10 °C increments. At each temperature setpoint, the reactor was allowed to stabilize for 20 min, after which the outlet stream was analyzed for 30 min. Each condition was measured at least three times. The reactor effluent was then analyzed on-line for 30 min by gas chromatography.

Online GC analysis and calibration

The reactor outlet stream was analyzed using an online gas chromatograph (GC-2014, Shimadzu). Methanol was quantified using a flame ionization detector (FID) equipped with an HP-PLOT Q column. Permanent gases were quantified using a thermal conductivity detector (TCD) equipped with Molecular Sieve and Porapak Q columns.
Methanol formation was quantified from the GC peak area using an external calibration curve. The calibration gave a linear relationship between methanol amount and GC area with a slope of 67.266 ± 1.182, expressed as Area × 10^3 = 67.266 × CH_3OH mol × 10^8. Accordingly, the amount of methanol in each GC injection was obtained from the measured peak area. Using a 1.0 mL GC sampling loop, the methanol space-time yield was calculated as $\mathrm{STY_{MeOH}} = \frac{n_{\mathrm{MeOH,loop}} \times Q \times 60 \times M_{\mathrm{MeOH}}}{V_{\mathrm{loop}} \times m_{\mathrm{cat}}}$, where $n_{\mathrm{MeOH,loop}}$ is the methanol amount per GC injection, Q is the total outlet flow rate, V_{loop} is the loop volume, and m_{cat} is the catalyst mass. The resulting $\mathrm{STY_{MeOH}}$ values are reported in g kg_{cat}^{-1} h^{-1}.

Arrhenius fitting

Apparent activation energies for methanol formation were extracted independently at each GHSV by fitting the temperature-dependent $\mathrm{STY_{MeOH}}$ to an Arrhenius-type expression, $\ln(\mathrm{STY_{MeOH}}) = \ln A - E_{\mathrm{app}}/RT$, using ordinary least-squares regression of $\ln(\mathrm{STY_{MeOH}})$ against $1/T$ (T in K). To retain only the quasi-linear fitting region, data up to 210 °C were used for all flow rates, with the lower fitting boundary set to 160 °C. The apparent activation energy was calculated from the fitted slope according to $E_{\mathrm{app}} = -R \times \mathrm{slope}$, where $R = 8.314$ J mol^{-1} K^{-1}. For visualization, the Arrhenius plots are shown as $\ln(\mathrm{STY_{MeOH}})$ versus $1000/T$.

Experimental outcome and mechanistic interpretation.

The temperature-dependent methanol productivity and the corresponding Arrhenius plots are shown in **Fig. 7**. The three flow conditions exhibit distinct $\mathrm{STY_{MeOH}}$ profiles, and the highest flow rate gives the highest methanol productivity in the intermediate-temperature window. At higher temperature, however, the $\mathrm{STY_{MeOH}}$ values deviate from a single low-temperature Arrhenius trend, which motivated restriction of the fitting

procedure to the quasi-linear region. Within this region, the methanol-based apparent activation energy increased monotonically with flow rate, from 57.0 ± 4.5 kJ mol^{-1} at 750 cm^3 g_{cat}^{-1} h^{-1} to 76.6 ± 13.2 kJ mol^{-1} at 1500 cm^3 g_{cat}^{-1} h^{-1} and 93.9 ± 6.8 kJ mol^{-1} at 3000 cm^3 g_{cat}^{-1} h^{-1}. These measurements are therefore consistent with the positive GHSV–E_{app} trend inferred from the literature-trained ML model under the present low-pressure operating window.

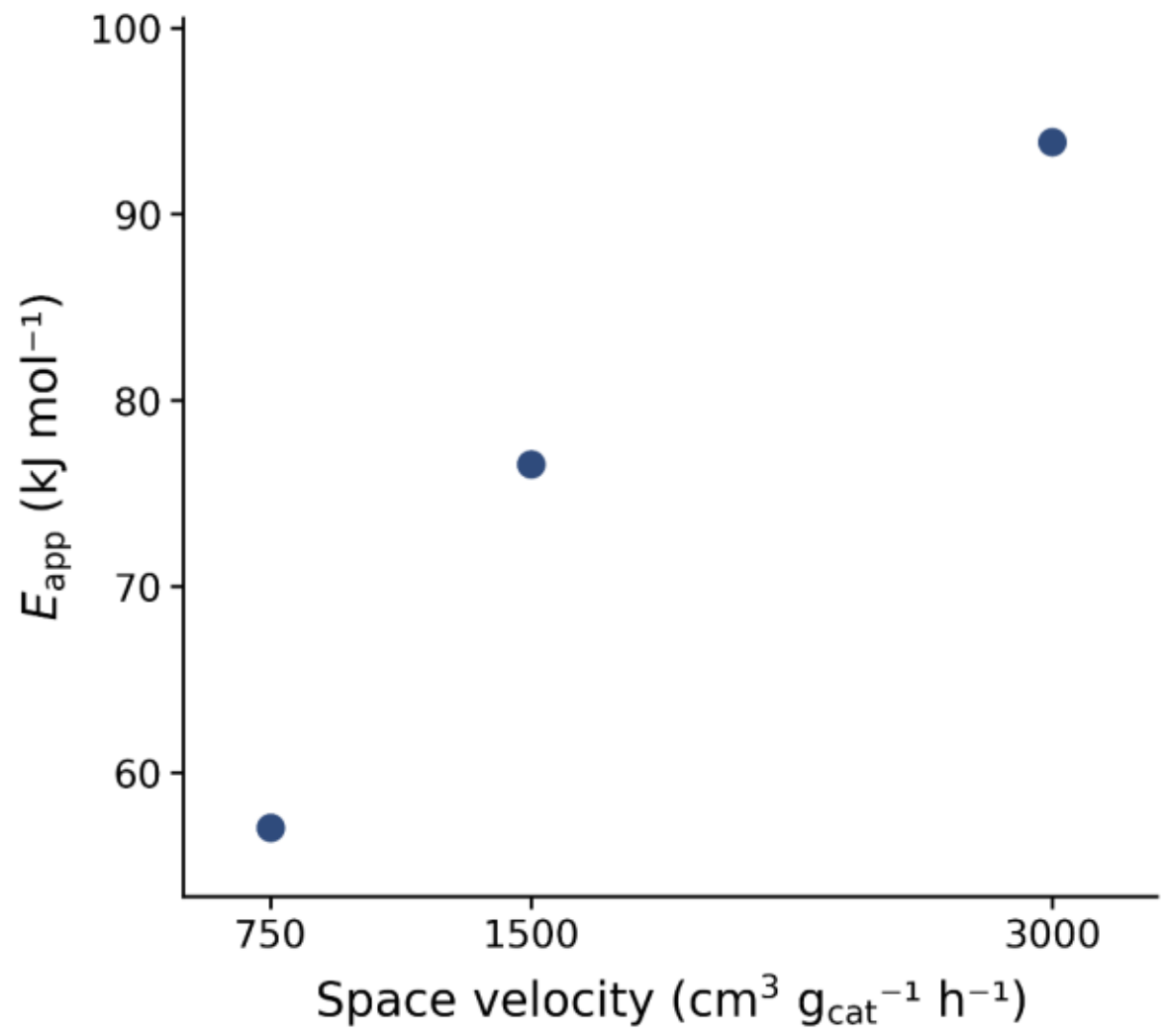

Figure S22. Experimental validation of the ML-derived GHSV–Eapp relationship. Methanol-based apparent activation energies extracted from Arrhenius fits of STY_{MeOH} increase monotonically from 57.0 ± 4.5 to 93.9 ± 6.8 kJ mol^{-1} as GHSV increases from 750 to 3000 cm^3 g_{cat}^{-1} h^{-1} over a commercial $Cu/ZnO/Al_2O_3$ catalyst at 1 bar. The monotonic trend supports the positive GHSV–E_{app} relationship inferred from the literature-trained model and is consistent with a flow-induced shift in the observed kinetic regime.

The mechanistic meaning of this result should be confined to observed kinetics. The E_{app} derived from reactor-level STY_{MeOH} is an operational quantity and should not be interpreted as direct evidence that flow rate changes an intrinsic elementary activation barrier.[2] For Cu-based CO_2 hydrogenation, methanol formation competes with RWGS, and the observed methanol rate can depend strongly on the operating window. Literature on Cu-based systems also suggests that H_2O may influence methanol-forming pathways under some conditions, but the underlying mechanism remains under active debate.[3] Accordingly, varying the flow rate can alter residence time and the local reaction environment along the bed, including the extent of product build-up and the relative weighting of parallel pathways, thereby changing the fitted methanol-based E_{app}. Because no operando characterization or dedicated transport analysis was performed here, the present experiment is best interpreted as demonstrating a GHSV dependence of the observed methanol-based E_{app} under this specific low-pressure condition, rather than identifying a unique mechanistic origin or a universal causal law.

Reviewer #3:

Comment 3-0:

I was pleased to read the study by Yan et al., entitled "A Machine Learning Perspective on Three Decades of Methanol Synthesis: Research Framework and Experimental Operation Insights". The study aims to apply machine learning to identify optimal operating conditions in heterogeneous catalysis. The authors claim that their data-driven approach uncovers previously underexplored operating windows for achieving high methanol productivity in CO_2-to-methanol synthesis. Furthermore, they argue that the apparent activation energy should not be regarded as an intrinsic material property, but rather as a reaction-condition-dependent observable. Based on the fundamental limitations outlined in the following comments regarding generality, scope, and overclaimed novelty, I recommend rejection of the manuscript in its current form, with the possibility of resubmission should the authors substantially broaden the applicability of the framework and revise the central conceptual claims.

Response:

We sincerely thank the reviewer for the careful reading and constructive critique regarding our work. The original submission overstated the breadth of the framework and did not sufficiently distinguish methodological transferability from data availability. In the revised manuscript, we substantially recalibrate the scope of our claims and reposition the main contribution as a reproducible, human-in-the-loop literature-to-ML workflow (data schema, preprocessing, symmetry handling, model selection with regularization, and interpretable mapping), demonstrated on a canonical, data-rich catalytic system. We also revise the activation-energy discussion to avoid any implication that "Ea being condition-dependent" is a newly discovered definition-level concept. Instead, we clarify that our contribution is the quantitative, data-driven mapping of how the apparent activation energy (E_{app}) varies across heterogeneous experimental regimes in the literature, and the resulting implications for fair comparison and reporting practice. An additional CO2RR catalytic system was also added to prove the applicability of the ML framework. We hope that our revisions satisfactorily address the concerns raised and meet the reviewer's expectations.

Comment 3-1:

The machine-learning framework is rigorous. However, the generality of the authors' claims should be reconsidered. The approach relies on an unusually large body of literature data (200 papers spanning three decades), which limits its practical applicability. For most reactions and emerging catalyst systems, such extensive datasets are unlikely to exist, restricting the framework's use as a broadly deployable guidance tool. The main strength of the approach lies in extracting design rules from mature, well-studied catalytic systems.

Response:

Thank you for this thoughtful comment. We also totally agree with you that mature, well-studied catalytic systems (i.e., data availability and curation quality) are central practical

constraints for any data-driven catalysis framework. We have therefore revised the manuscript to explicitly distinguish the transferability of the workflow from the availability of sufficiently curated data. In other words, what we claim to be transferable is the workflow architecture, not an unconditional guarantee of predictive guidance for data-sparse emerging systems. Moreover, we have added one additional catalytic system, to demonstrate that the framework remains viable in substantially smaller-data regimes, as detailed in the response of next Comment 3-2. We additionally compiled a cross-study comparison of experimental dataset sizes used in heterogeneous-catalysis ML studies to place the present dataset in context. The detailed revisions are shown below.

While our literature survey spans 200 publications, the model training uses 2,383 curated, permutation-invariant experimental entries. We believe that this scale is moderate for machine learning on experimental catalysis data, and the key question is not the number of papers, but how many high-quality, consistently defined data points are available after harmonized curation.

To place this scale in context, we have compiled a new comparison table and figure (**Table S3** and **Fig. S23**) summarizing dataset sizes in representative ML studies based on experimental data in heterogeneous catalysis. Across reaction classes, experimental dataset sizes span from single-digit to over ten thousand data points, indicating that datasets on the order of 10^3 are not atypical once multiple sources are harmonized. The surveyed studies cover thermocatalysis (e.g., PDH, DRM, WGS, OCM, etc.), electrocatalysis (e.g., CO_2RR, NRR, OER/HER), photocatalysis, organic catalysis, and process-related datasets. CO_2 thermocatalysis (e.g., CO_2-to-methanol and CO_2-to-olefins) is listed separately. It is worth noting that the dataset of our work is the most extensive in the CO_2-to-methanol.

Table S3 The experimental dataset size found in machine-learning studies of heterogeneous catalysis organized by reaction type

**Reaction type**	**Dataset sizes (number of datapoints)**	**Methods**	**References (DOI)**
Dry reforming of methane (DRM)	5508;	DT; ANN	10.1016/j.ijhydene.2016.10.003;
Dry reforming of methane (DRM)	5521	DT; ANN	10.1016/j.cattod.2017.05.012
Dry reforming of methane (DRM)	27	ANN	10.1016/j.ijhydene.2016.04.034
Dry reforming of methane (DRM)	100	ANN; SVM; RT	10.1016/j.jechem.2024.04.022
CO methanation	63	K-means algorithm;Gaussian process regression (GPR)	10.1039/c7cy01634d
Oxidative coupling of methane (OCM)[a]	1870	DT	10.1002/cctc.201100186

Oxidative coupling of methane (OCM)[a]	1802;	Regression analysis	10.1038/s41467-019-08325-8;
Oxidative coupling of methane (OCM)[a]	1868	RF	10.1002/cctc.201800310
Oxidative coupling of methane (OCM)[a]	1870	Quadratic response surface model; clustered RBF neural network; RF	10.1039/c4cy01443j
Oxidative Coupling of Methane[a]	12708	Gaussian mixture model	10.1021/acs.jpclett.0c01926
CO oxidation	5610	DT; ANN; genetic algorithm-based clustering technique	10.1002/cctc.201200665
CO oxidation	1337	Neural Network	10.1021/ie2013955
CO oxidation	882	ANN	10.1016/j.apcata.2013.08.056
CO oxidation	252	DT; ANN	10.1016/j.cherd.2012.08.017
Water-gas shift reaction	4360	DT; ANN; SVM	10.1016/j.ijhydene.2014.01.160
Water-gas shift reaction	2228	ANN; PCA (Principal Component Analysis)	10.1016/j.apcatb.2019.118257
Water-gas shift reaction	344	ANN	10.1002/qsar.200430900
Supercritical water gasification	718	RF; SVM; ANN	10.1016/j.cej.2021.131285
Electrocatalytic reduction of CO_2	471	DT	10.1016/j.jcou.2018.09.011
Electrocatalytic reduction of CO_2	127	DT; RT; Neural Network	10.1021/acssuschemeng.2c02941
Electrocatalytic reduction of CO_2	5196	neural network	10.1021/acscatal.4c05530
CO_2 hydrogenation to methanol	1201	RF; GBDT; XGB	10.1016/j.apcatb.2022.121530
CO_2 hydrogenation to methanol	212	ANN	10.1016/j.fuel.2023.128045
Photocatalytic water splitting	540	Association rule mining (Apriori); DT; RF	10.1016/j.apcatb.2018.09.104
Photocatalytic valorization/reforming of glycerol to H_2	29	ANN; Genetic algorithm (GA)	10.1016/j.apcatb.2017.03.016
Electrocatalytic hydrogen evolution reaction (HER)	38	Reshef pattern recognition algorithm	10.1021/ja407394q
OER and HER	134	RT	10.1002/adma.202420465
Electrocatalytic oxygen evolution reaction (OER)	70	Multiobjective Bayesian optimization	10.1039/d3ta06651g

Electrocatalytic oxygen evolution reaction (OER)	101	Ordinary least squares; least angle regression	10.1021/acs.jpcc.5b10071
Electrocatalytic oxygen evolution reaction (OER)	2121	Gemini; Bayesian optimizer Phoenics; Bayesian neural network	http://arxiv.org/abs/2103.03391
Electrocatalytic oxygen evolution reaction (OER)	25	ANN	10.1016/j.cattod.2010.06.019
Electrocatalytic oxygen evolution reaction (OER)	2121	Sequential learning	10.1039/C9SC05999G
Selective CO_2 hydrogenation	17	ANN; RSM	10.1016/j.jcou.2017.11.013
Photocatalytic CO_2 Reduction	103	Bayesian optimization	10.1021/jacs.4c01305
Asymmetric hydrogenation of olefins	11932	GNN	10.1038/s41467-025-58854-8
Terminal olefin hydroformylation	1167	ET; NN; XGB; DT; GPR;LGBM; KNR; KRR; CBR	10.1016/j.checat.2024.101079
CO_2 to light olefins	532	DNN; SVR; RF; AdaBoost; HistG-Boost; XGBoost	10.1002/aic.18437
Photocatalytic glycerol valorization to hydrogen	29	Response surface methodology; ANN	10.1016/j.apcatb.2017.03.016
Reverse osmosis desalination	838	DT	10.1016/j.jclepro.2025.146979
lipid production from Yarrowia lipolytica	356	DT	10.1016/j.fuel.2021.122817
tri-reforming of biogas	1183	RF; DT	10.1016/j.ijhydene.2025.04.213
Electrochemical nitrogen reduction reaction (NRR)	520	Linear regression, decision tree, random forest, and multilayer perceptron	10.1039/D3DD00151B
Propane Dehydrogenation	10155	Decision Tree (DTR), Random Forest (RFR), CatBoost, Extra Tree (ETR), XGBoost, Gradient Boosting (GBR), LightGBM, Hist Gradient Boosting (HGB), Gaussian	10.1021/acscatal.5c06285

		Process (GPR), Support Vector (SVR) regression algorithms, Artificial Neural Networks (ANN), Deep Neural Networks (DNN)	

[a] Data collected through high-throughput experiments. Nguyen, T. N.; Nhat, T. T. P.; Takimoto, K.; Thakur, A.; Nishimura, S.; Ohyama, J.; Miyazato, I.; Takahashi, L.; Fujima, J.; Takahashi, K.; Taniike, T. High-Throughput Experimentation and Catalyst Informatics for Oxidative Coupling of Methane. *ACS Catal.* 2020, 10, 921–932.

[b] A significant break-through in terms of the dataset quality was reported by Zavyalova et al. in 2011.

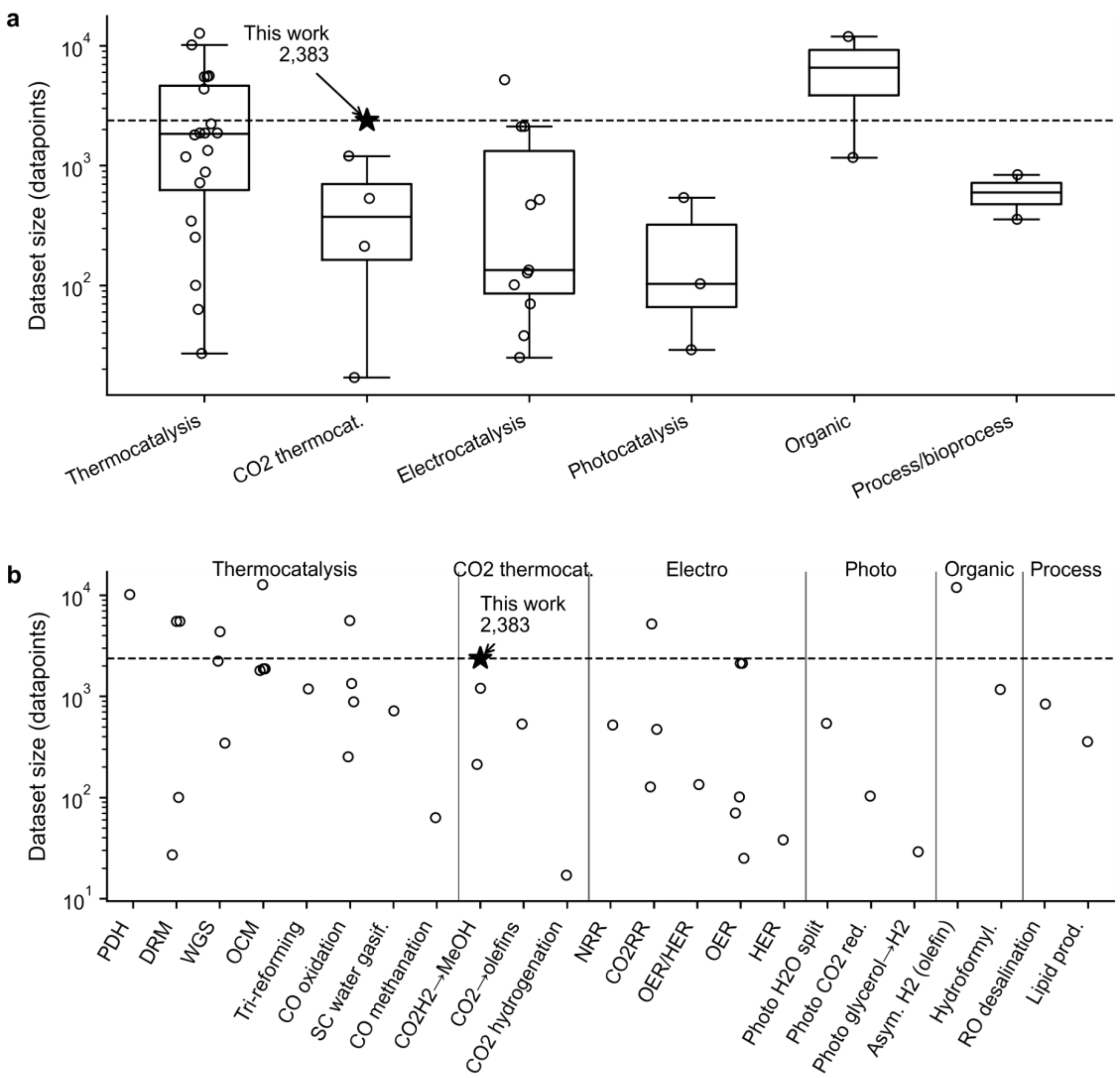

Figure S23. **Dataset sizes in experimental-data machine-learning studies of catalysis.** Boxplots (a, top) and individual datasets (b, bottom) summarize the number of curated data points per study across reaction domains (Table R1); the star and dashed line indicate this work (2,383 entries).

Abbreviations: PDH, propane dehydrogenation; DRM, dry reforming of methane; WGS, water–gas shift; OCM, oxidative coupling of methane; Tri-reforming, tri-reforming of biogas; SC water gasif., supercritical water gasification; CO2H2→MeOH, CO_2 hydrogenation to methanol; CO_2→olefins, CO_2 to light olefins; NRR, nitrogen reduction reaction; CO_2RR, CO_2 reduction reaction; OER, oxygen evolution reaction; HER, hydrogen evolution reaction; Photo H2O split, photocatalytic water splitting; Photo CO2 red., photocatalytic CO2 reduction; Photo glycerol→H2, photocatalytic reforming of glycerol to H_2; Asym. H2 (olefin), asymmetric hydrogenation of olefins; Hydroformyl., terminal olefin hydroformylation; RO desalination, reverse osmosis desalination; Lipid prod., lipid production from *Yarrowia lipolytica*.

At the same time, we recognize that a common limitation in experimental catalysis is the cost of data acquisition. In many heterogeneous catalysis studies, measurements are performed with limited instrumental automation, making each data point expensive in both human and material resources; consequently, datasets in emerging systems often remain at the scale of a few hundred entries. This is precisely why we view the construction and release of a machine-readable, consistently curated database as one of the contributions of this work. We also encourage the community to adopt more standardized reporting and data formats in future catalysis publications, which would meaningfully reduce curation burden and provide a foundation for more reliable automated data curation (further discussed in our response to the next comment).

We have revised the manuscript to avoid overstating universality and to explicitly quantify data requirements. The additional catalytic system with substantially smaller-data regimes had also been examined, and the details are shown in the response of next Comment 3-2.

Comment 3-2:

Closely related to Comment 1, the analysis is restricted to a single catalyst family (Cu/ZnO-based systems) and a single reaction (CO_2-to-methanol synthesis), resulting in a limited exploration of chemical degrees of freedom. While the framework is valuable for rationalizing trends within this well-established system, its practical generality remains unproven. To justify the breadth of the claims and the scope of the journal, the framework should be extended to include automated data curation and demonstrated on at least one additional catalytic system. If such an extension is not practically feasible, this would itself highlight the limited applicability of the framework, and the manuscript cannot be accepted in its current form.

Response:

We appreciate the reviewer's clear request and agree that demonstrating transfer beyond the Cu/ZnO methanol system strengthens the manuscript. In the revised manuscript, we addressed this concern in two complementary ways. First, we moderated the scope of our claims and now explicitly define the framework as a literature-driven interpretable ML workflow for catalytic systems in which sufficiently structured experimental data can be curated, rather than as a universally deployable or fully autonomous literature-mining tool. Second, and more importantly, we extended the workflow to an independent

catalytic system, electrochemical CO_2 reduction (CO_2RR), which is now described in new Section 2.6 and Supplementary Note 7.

Importantly, this is not a separate ad hoc model built on a different table, but the same end-to-end workflow used in the methanol case: descriptor schema definition, data extraction and harmonization, preprocessing, model training and selection, SHAP-based interpretation, and inference. The CO_2RR case study differs substantially from the main thermocatalytic system in operating mode, descriptor space, and product manifold, including electrocatalysis-specific variables such as applied potential, membrane, electrode support, electrolyte composition, and grouped product outputs H2, CO, C1, and C2+. Despite the smaller dataset size, the workflow produced stable predictive performance and recovered physically meaningful dominant drivers, with applied potential, active phase, membrane, particle size, and support emerging as the leading descriptors.

We further note that the revised manuscript now demonstrates transferability at two levels: within the Cu/ZnO methanol system, the same framework was successfully extended from performance outputs to the kinetic observable E_{app} and experimentally corroborated; across systems, the added CO_2RR case study shows that the workflow is transferable from fixed-bed thermocatalysis to electrocatalysis. We believe these additions directly address the reviewer's concern regarding practical generality while also clarifying the realistic current scope of literature-driven catalytic ML.

As for the automated data curation, we also agree that automation is important for scalability. At the same time, we note that high-fidelity catalysis data in the literature are commonly reported in non-standardized formats (tables, figures, and narrative text) with inconsistent units and definitions, often requiring domain interpretation by experts to ensure semantic correctness. For this reason, fully autonomous, end-to-end extraction without expert verification is currently not sufficiently reliable for constructing a benchmark-quality dataset. To address this in a constructive and actionable way, we revised the manuscript to explicitly describe our curation as a human-in-the-loop workflow with automated standardization and conflict-resolution steps. Specifically, our pipeline includes script-based unit harmonization, feature normalization, duplicate handling, and an automated symmetry/conflict-resolution algorithm that yields the final permutation-invariant dataset used for training (**Fig. 2**). We also provide the structured dataset and curation scripts to enable reuse and extension by other researchers. This limitation is of course described in the Discussion.

However, we believe this revision satisfies the goal of deployability in a realistic sense. The ML workflow is general and reproducible, the automation is implemented where it is reliable, and the resulting dataset and code lower the barrier for applying the framework to other reactions and catalyst families.

Supplementary Note 7. Cross-system transferability test on electrochemical CO2 reduction:

Moreover, one additional catalytic system is chosen to demonstrate that the ML framework remains viable in substantially smaller-data regimes. We selected Cu-based electrochemical CO_2 reduction (CO_2RR) as an auxiliary transferability test because it differs fundamentally from the main thermocatalytic methanol-synthesis case in reaction environment, driving force, descriptor space, and product manifold, while remaining a catalytically complex CO_2-conversion chemistry amenable to literature curation.

Building on our previous study,[4] we assembled a curated CO_2RR dataset comprising 379 literature-derived entries. Each entry corresponds to a unique catalyst–electrolyte–operation point. The input descriptors comprised Cu-based catalyst phase, Cu-based catalyst shape, electrode support, particle size, applied potential versus RHE, electrolyte cation identity and concentration, electrolyte anion identity and concentration, membrane type, and, where available, current density. The outputs were grouped product fractions of H2, CO, C1, and C2+. All potentials were converted to V versus RHE, particle sizes to nm, and electrolyte concentrations to M. Categorical variables were encoded analogously to the main thermocatalytic workflow, and numerical variables were transformed and standardized where appropriate. Descriptor distributions after preprocessing are shown in **Fig. S24**.

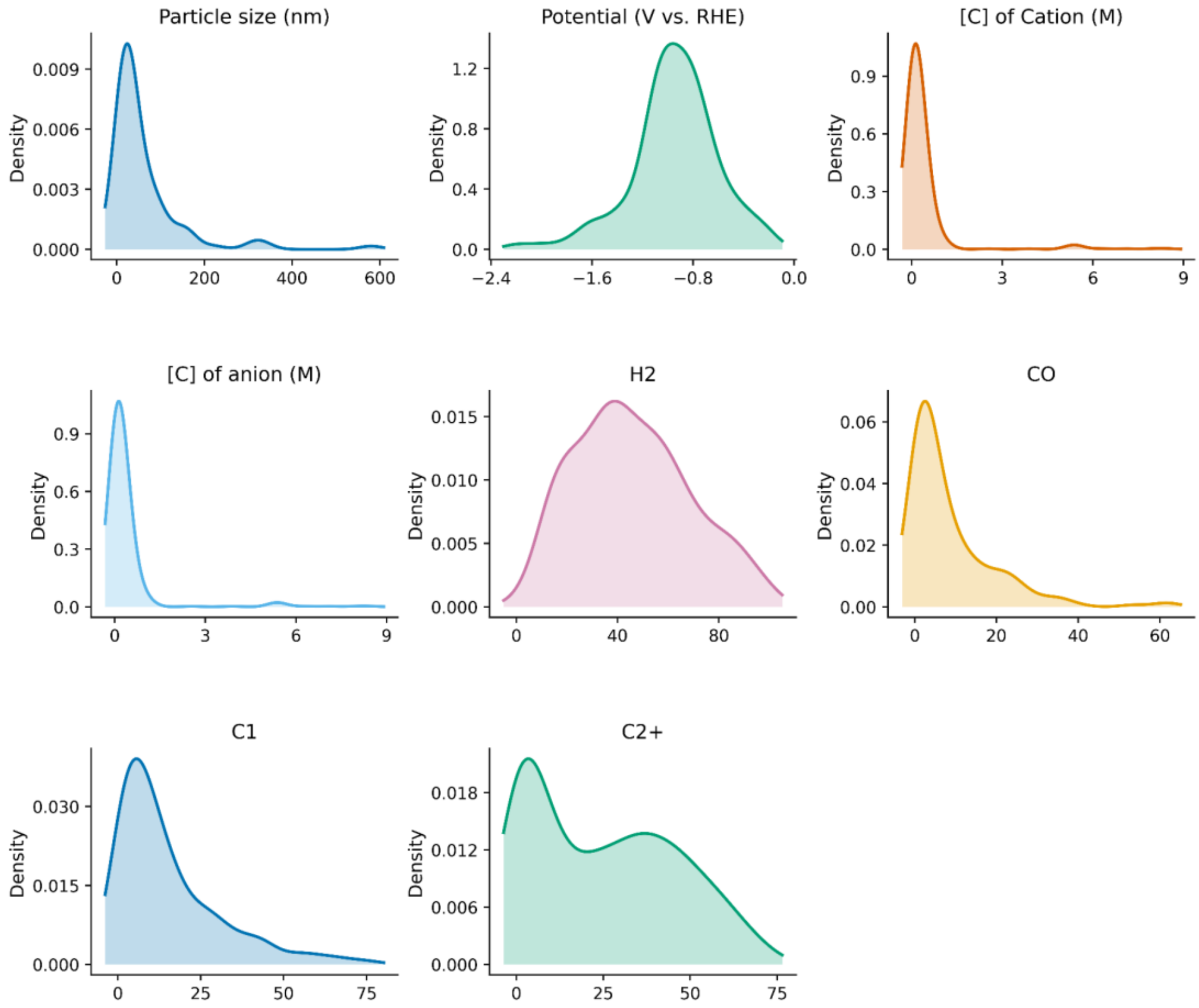

Figure S24. Distributions of selected numerical features after preprocessing.

We then applied the same strategy as in the main thermocatalytic dataset, evaluating six ML models under an identical tuning and validation protocol. CatBoost delivered the strongest overall predictive performance. Although the unregularized CatBoost model gave the highest aggregate CV R^2, it also displayed pronounced overfitting. We therefore retained the CatBoost model with α = 0.3 as the best candidate. This model achieved validation R^2 values of 0.625 for H_2, 0.714 for CO, 0.755 for C1, and 0.708 for C2+, with an aggregate CV R^2 of 0.736 (**Table S4**). These results show that the framework retains predictive usefulness even in this smaller-data electrocatalytic setting.

Table S4. Historical comparison of per-output validation R^2, aggregate CV R^2, and overfitting metrics for six ML models at different regularization strengths (α)

**Model**	**α**	**R^2 (Valid)**				**Overfitting**		**CV (R^2)**
		**H_2**	**CO**	**C1**	**C2+**	**MSE Ratio**	**R^2 difference**	
RF	0.0	0.520	0.637	0.643	0.677	5.57	0.31	0.691
	0.03	0.353	0.484	0.446	0.469	1.53	0.21	0.490
	**0.3**	**0.515**	**0.618**	**0.625**	**0.670**	**4.20**	**0.30**	**0.679**
	0.5	0.507	0.614	0.605	0.658	3.48	0.29	0.670
	0.7	0.515	0.629	0.633	0.674	4.58	0.31	0.680
CatBoost	0.0	0.661	0.747	0.794	0.717	47.55	0.26	0.759
	0.03	0.635	0.735	0.761	0.709	12.41	0.27	0.746
	**0.3**	**0.625**	**0.714**	**0.755**	**0.708**	**7.14**	**0.26**	**0.736**
	0.5	0.532	0.610	0.682	0.653	2.82	0.25	0.678
	0.7	0.483	0.566	0.671	0.616	2.53	0.26	0.657
DT	0.0	0.414	0.244	0.541	0.374	3.02	0.41	0.504
	**0.03**	**0.411**	**0.229**	**0.554**	**0.253**	**6.21**	**0.54**	**0.528**
	0.3	0.405	0.266	0.387	0.371	1.14	0.10	0.312
	0.5	0.405	0.266	0.387	0.371	1.14	0.10	0.312
	0.7	0.342	0.195	0.451	0.405	1.27	0.15	0.304
ANN	**0.0**	**0.379**	**0.276**	**0.539**	**0.434**	**1.61**	**0.24**	**0.591**
	0.03	-0.035	0.058	-0.012	-0.114	1.07	0.10	0.306
	0.3	0.298	0.203	0.517	0.416	1.51	0.23	0.575
	0.5	0.286	0.254	0.545	0.396	1.55	0.24	0.574
	0.7	0.288	0.261	0.482	0.438	1.39	0.19	0.617
XGB	0.0	0.591	0.503	0.723	0.719	55.50	0.36	0.713
	0.03	0.544	0.452	0.696	0.709	22.35	0.38	0.682
	0.3	0.575	0.494	0.740	0.725	52.89	0.36	0.711

	**0.5**	**0.546**	**0.523**	**0.700**	**0.712**	**10.81**	**0.35**	**0.693**
	0.7	0.557	0.489	0.745	0.685	22.20	0.36	0.707
SVM	0.0	0.573	0.316	0.706	0.575	4.23	0.35	0.611
	0.03	0.446	0.255	0.730	0.498	1.80	0.24	0.553
	0.3	0.501	0.416	0.689	0.572	2.90	0.30	0.597
	0.5	0.467	0.433	0.716	0.564	2.51	0.28	0.604
	**0.7**	**0.513**	**0.407**	**0.715**	**0.595**	**2.72**	**0.28**	**0.609**

Interpretation of the selected model further supports its chemical plausibility. SHAP analysis identified applied potential as the dominant descriptor across all four outputs. Particle size emerged as the leading continuously varying structural descriptor, whereas the relative contributions of active phase, electrode support, membrane type, and shape depended on the product group. By contrast, electrolyte identity and concentration exerted secondary influence within the sampled domain (**Fig. S25a–d**). This ranking is physically sensible for CO_2RR, where interfacial driving force, catalyst identity, and local transport environment govern the competition between HER and carbon-containing products.

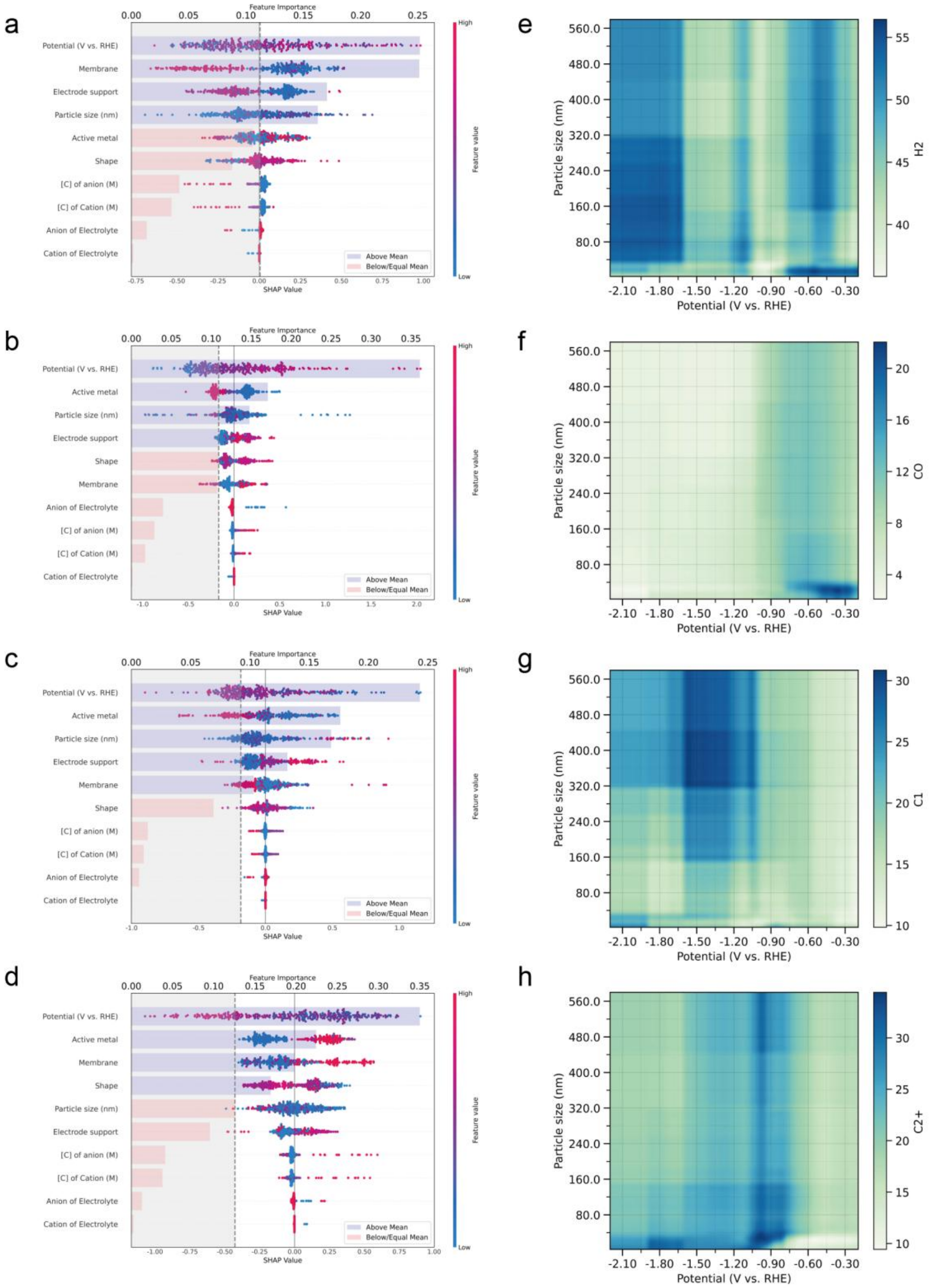

Figure S25. SHAP summaries (a–d) and potential–particle size response maps (e–h) for H_2, CO, C1, and C2+ predicted by the selected CatBoost model (α = 0.3).

To translate descriptor rankings into response patterns, we generated pairwise inference maps using the two leading continuously varying variables, applied potential and particle size. The resulting landscapes were output-specific and clearly non-monotonic (**Fig. S25e–h**). H_2 was favored at more cathodic potentials across broad size windows, whereas CO increased toward less negative potentials and smaller particles. C1 reached higher predicted fractions in an intermediate potential regime and over mid-to-large

particle sizes, while C2+ occupied a narrower region jointly determined by potential and particle size. These patterns indicate that the framework captures descriptor interactions rather than simple one-variable trends.

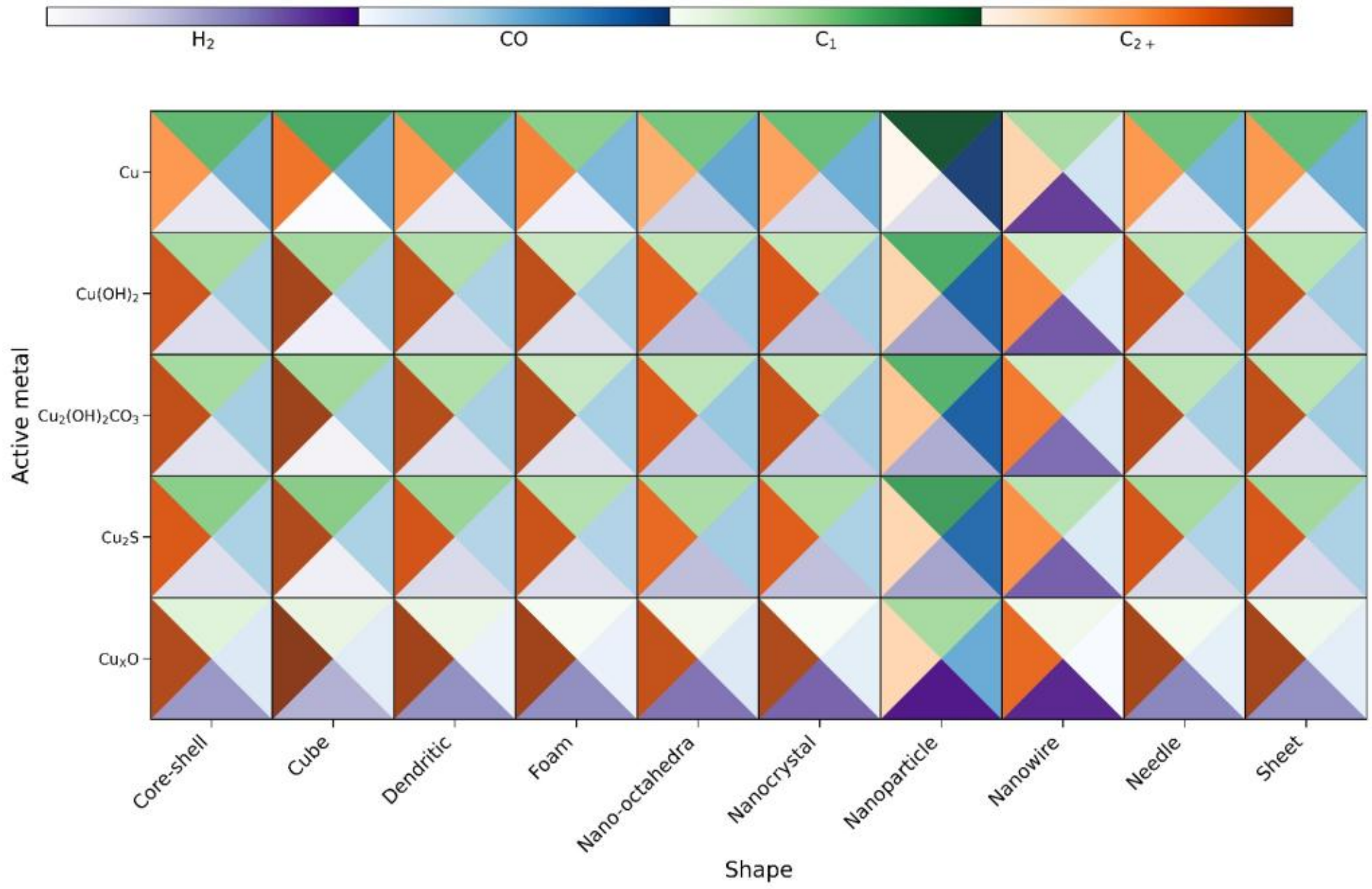

Figure S26. Active phase–shape map of predicted H2, CO, C1, and C2+ fractions from the selected CatBoost model (α = 0.3).

A complementary categorical inference map across active phase and catalyst shape further showed that product distributions are jointly reorganized by phase identity and morphology (**Fig. S26**). Rather than pointing to a single universally optimal phase–shape pair, these predictions emphasize that different combinations redistribute the balance among H_2, CO, C1, and C2+ channels in distinct ways, consistent with the strong morphology- and phase-sensitivity of CO_2RR.

This auxiliary case study is not intended to establish a universal predictive model for all CO_2RR chemistries, nor to claim full automation of literature extraction. Rather, it serves as a transferability test of the workflow itself. The recovery of chemically meaningful electrocatalytic drivers and interpretable multidimensional response surfaces across a reaction regime fundamentally different from thermocatalytic methanol synthesis supports the broader applicability of the present literature-driven interpretable machine-learning framework when sufficiently structured data can be curated.

Comment 3-3:

The authors claim to reveal that the activation energy (Ea) is a reaction-condition-dependent property. This claim is not acceptable. By definition, Ea is an empirical parameter characterizing the temperature dependence of a reaction rate coefficient and is therefore inherently condition dependent. Consequently, this statement does not constitute a novel finding, and the importance of the work cannot be based on this claim.

Response:

We sincerely thank the reviewer for this important clarification. We fully agree that an activation energy extracted from Arrhenius analysis is, by definition, an apparent and condition-dependent quantity. Accordingly, any wording suggesting that this definitional point constitutes a novel discovery has been removed. Instead, the contribution of our analysis is clearly written as the data-driven mapping of where apparent Arrhenius slopes vary most strongly across the historical operating and synthesis space, thereby identifying regime markers that motivate targeted mechanistic tests.

Importantly, we further tested one of the ML-derived hypotheses experimentally by varying only GHSV in a controlled fixed-bed experiment over a commercial $Cu/ZnO/Al_2O_3$ catalyst, which yielded a monotonic increase in methanol-based E_{app} from57.0 ± 4.5 to 93.9 ± 6.8 kJ mol^{-1} across the tested space-velocity range.

Accordingly, we have implemented the following specific revisions.

In the Abstract: We replaced the previous sentence implying that we “reveal” condition dependence as a principal finding. The revised abstract now states that the curated E_{app} subset is used to map regime-sensitive kinetic signatures (GHSV ridge and non-monotonic pressure dependence) that motivate mechanistic tests.

In the Section 2.5 (main text, Deriving kinetic insight from the ML framework): We rewrote the opening and interpretive paragraphs to explicitly define E_{app} as an empirical Arrhenius slope evaluated within a specified operating window, and we added a short derivation showing that E_{app} generally contains contributions from temperature-dependent coverage/thermodynamic terms (Eqs. 2–4). We also revised the discussion of ridge/turning-point features to avoid over-attributing specific microscopic pathways in the absence of dedicated mechanistic experiments.

In the Supplementary Note 3: We expanded the description of the activation-energy subset construction to clarify that the fits are performed on methanol formation rate proxies (typically STY_{MeOH}), and we specified the inclusion criteria and quality-control steps used to obtain the final E_{app} dataset.

We also did additional experiments for Experimental validation of the ML-derived GHSV–E_{app} relationship.

2.5 Deriving kinetic insight from the ML framework

To assess the physical relevance and generalizability of our ML framework beyond empirical performance correlations, we analyzed a curated kinetic subset of apparent activation energies for methanol formation, E_{app}. These values were extracted from the literature when explicitly reported or regenerated by fitting temperature-series methanol formation rate (typically STY_{MeOH} measured at multiple temperatures under otherwise fixed catalyst and feed conditions) to the Arrhenius equation (see *Supplementary Note 3* for details). Here, E_{app} is defined as the empirical Arrhenius slope of the observed methanol formation rate r:

$$E_{\mathrm{app}} \;=\; -R\left(\frac{\partial \ln r}{\partial(1/T)}\right)_{p,y_i,\mathrm{GHSV}} \tag{2}$$

where R is the universal gas constant, T is the absolute temperature, and p, y_i, GHSV denote the total pressure, feed composition (or partial pressures) for all gas-phase species i, and space-velocity window reported for each temperature series. As such, E_{app} is not expected to coincide with a unique elementary-step barrier, because the observed rate generally reflects both intrinsic kinetics and temperature-dependent coverage or inhibition effects. A compact representation of observed rate is

$$r \;=\; k_{\mathrm{int}}(T)\, f\big(\{p_i\}, \{\theta_j(T, \{p_i\})\}\big) \tag{3}$$

where $k_{int}(T)$ denotes the intrinsic temperature-dependent rate constant for the kinetically relevant event, while the function f captures coverage and thermodynamic driving-force effects through the set of fractional surface coverages $\{\theta_j\}$ of adsorbed intermediates j. Differentiation yields

$$E_{\mathrm{app}} \;=\; E_{\mathrm{int}} \;-\; R\left(\frac{\partial \ln f}{\partial(1/\mathrm{T})}\right)_{\{p_i\}} \tag{4}$$

showing that E_{app} generally contains additional contributions whenever f varies with temperature. In heterogeneous catalysis, such variations commonly arise because adsorption equilibria and surface coverages depend on both T and $\{p_i\}$, so that changes in pressure or feed composition can systematically shift E_{app} even for the same catalyst. Moreover, transitions in the rate-determining step within the experimental window, potentially driven by surface coverage evolution, water inhibition, or transport constraints, can lead to curvature in Arrhenius plots. Such non-Arrhenius behavior results in variable or discontinuous apparent activation energies, which likely accounts for the broad range of literature values reported for Cu/ZnO-based catalysts, even among nominally identical formulations.

Therefore, E_{app} should be treated as a regime-sensitive kinetic observable and the ML framework can be used to map where such regime sensitivity is most pronounced across the literature-derived feature space, which may serve as the transition points of the reaction pathway. Training on the same feature space used previously, the prediction accuracy for E_{app} evaluated by R^2 and MSE of each model with different α was summarized in **Table S2**. As a result, XGB model at α = 0.001 achieves high accuracy (R^2 = 0.916; **Fig. 6a**). The SHAP analysis (**Fig. 6b**) shows that promoter chemistry emerges as the leading contributor to the kinetic landscape, followed by calcination temperature, GHSV, and the Zn:Cu molar ratio, whereas pressure, synthesis procedure, and promoter loadings exert secondary effects within the sampled domain. Notably, promoter chemistry contributes more strongly than promoter loading, indicating that elemental identity is a major discriminator of the reported apparent kinetic regimes.

To translate these ranked descriptors into interpretable kinetic maps, we generated pairwise inference heatmaps for the leading continuously varying variables while resolving promoter effects separately. **Figure 6c** identifies a low-E_{app} basin at intermediate calcination temperatures together with moderate Zn enrichment, consistent with an interfacial optimum in Cu/ZnO-based catalysts.[112] This pattern is consistent with the notion that calcination can tune catalyst structure and interfacial characteristics in ways that affect observed kinetics, while also underscoring that the literature spans heterogeneous preparation protocols and reporting practices. By contrast, **Fig. 6d** clearly shows E_{app} rises markedly upon entering the high GHSV regime, whereas pressure modulates this landscape slightly weakly. E_{app} in heterogeneous catalysis depends on surface coverage and the prevailing kinetic regime, we therefore interpret this association as indirect evidence that GHSV changes an intrinsic elementary barrier. The model associates higher GHSV with higher reported E_{app} in the compiled literature, which we interpret as an operational effect that may reflect residence-time-driven changes in local coverages (H* and H_2O)[113] and, consequently, the dominant pathway and the apparent Arrhenius slope. To directly evaluate the GHSV–E_{app} hypothesis suggested by the ML analysis, we additionally performed a controlled fixed-bed experiment at 1 bar using a commercial $Cu/ZnO/Al_2O_3$ catalyst by varying only the space velocity; the experimental details and discussion are provided in the subsequent section and Supplementary Information.

Because promoter identities were encoded through chemistry-informed descriptors under a canonical permutation-invariant representation, promoter effects must be interpreted at the pair level. Accordingly, **Fig. 6e** summarizes the predicted E_{app} landscape across promoter-slot combinations. The matrix indicates that the reported apparent kinetic behavior varies substantially across promoter-pair space, but these contrasts should be interpreted as screening-level guidance because promoter identity, synthesis history, and operating conditions are frequently co-varied in the literature.

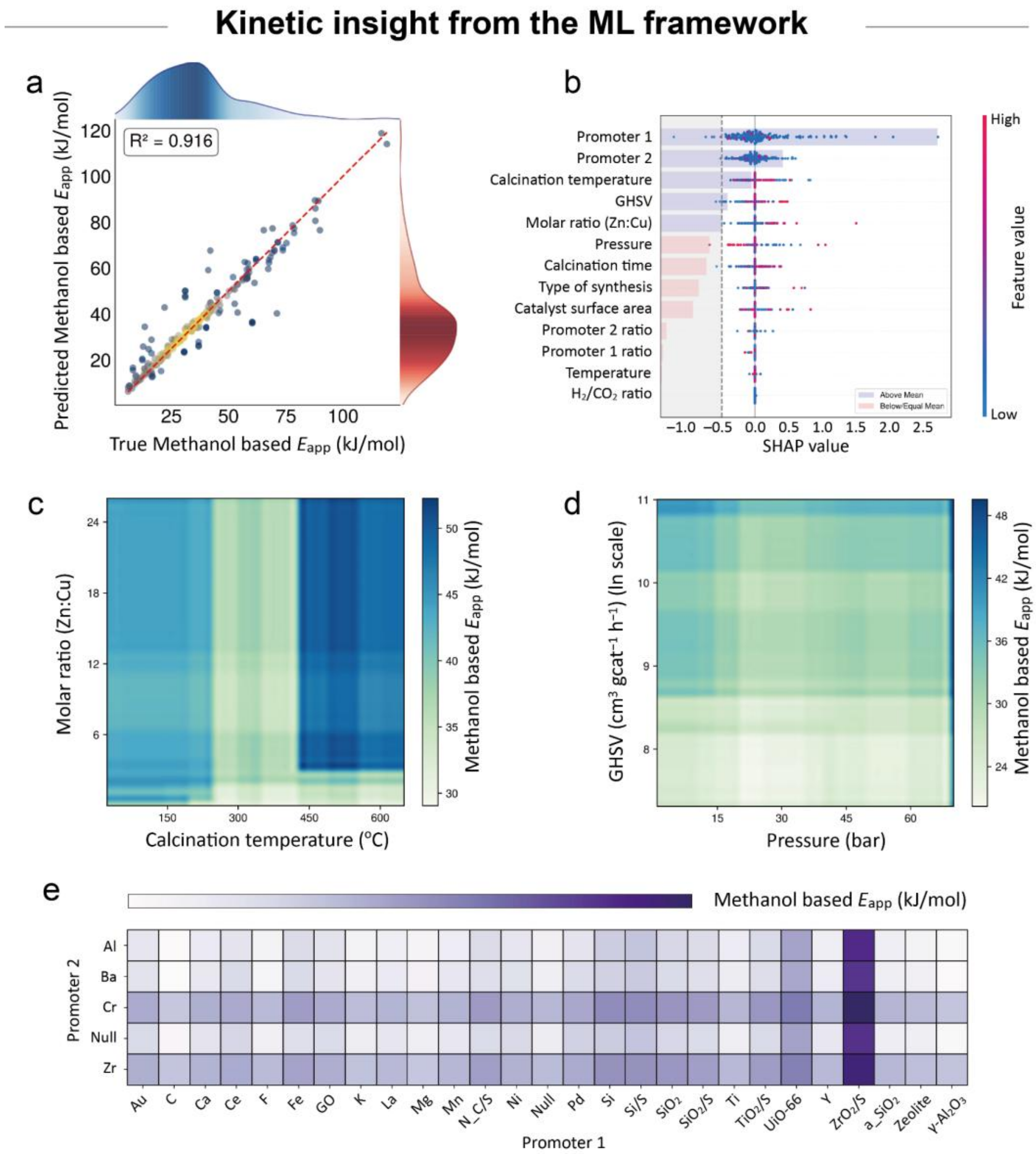

Figure 6. Kinetic insight from the ML model. (a) Predicted versus reported methanol-based apparent activation energy (E_{app}), showing good agreement for the XGB model at α = 0.001. (b) SHAP value for E_{app} prediction. (c,d) SHAP-guided pairwise inference heatmaps constructed from the leading continuously varying descriptors, showing the predicted E_{app} variation as a function of calcination temperature and Zn:Cu ratio (c) and of pressure and ln(GHSV) (d), with the remaining variables fixed at representative reference values. (e) Promoter-pair response matrix for E_{app} across the literature-covered promoter space. Promoter 1 and 2 do not imply mechanistically distinct primary and secondary roles.

2.6 Experimental validation and cross-system transferability of the ML framework

To directly test the ML-derived hypothesis that GHSV modulates the observed kinetic barrier for methanol formation, we performed controlled fixed-bed CO_2 hydrogenation experiments over a commercial $Cu/ZnO/Al_2O_3$ catalyst at 1 bar while varying only the flow rate. The catalyst identity, feed composition, and activation protocol were held constant,

and methanol productivity was measured between 160 and 240 °C at GHSV values of 750, 1500, and 3000 $cm^3\ g_{cat}^{-1}\ h^{-1}$. Detailed experimental procedures, Arrhenius fitting, and mechanistic discussion are provided in *Supplementary Note 6*.

Apparent activation energies were extracted from the quasi-linear Arrhenius regions by fitting ln(STYMeOH) against 1000/T (**Fig. 7**). The experimentally determined methanol-based E_{app} increased monotonically with GHSV, from 57.0 ± 4.5 kJ mol^{-1} at 750 $cm^3\ g_{cat}^{-1}\ h^{-1}$ to 76.6 ± 13.2 kJ mol^{-1} at 1500 $cm^3\ g_{cat}^{-1}\ h^{-1}$ and 93.9 ± 6.8 kJ mol^{-1} at 3000 $cm^3\ g_{cat}^{-1}\ h^{-1}$ (**Figs. 7 and S22**). Because only the flow rate was changed while the catalyst, pressure, and feed ratio were fixed, this experiment isolates GHSV as an operational variable and directly corroborates the positive GHSV–E_{app} relationship inferred from the literature-trained ML model. Such behavior is consistent with prior heterogeneous catalysis studies showing that apparent activation energies can remain insensitive to, or become strongly modulated by, flow and residence-time changes depending on the prevailing kinetic regime and surface-coverage state.[114–117]

Direct literature on a GHSV-dependent methanol-based apparent activation energy in CO_2-to-methanol synthesis remains limited. Under a higher-pressure operating window, Bukhtiyarova and co-workers reported nearly unchanged methanol E_{app} values over a commercial $Cu/ZnO/Al_2O_3$ catalyst across the investigated space-velocity range, indicating that space velocity did not perturb the controlling regime under those conditions.[118] By contrast, in other heterogeneous catalytic systems, residence time or space velocity has been shown to influence measured apparent activation energies and regime transitions.[114,115] These precedents support interpreting the present result as a flow-induced change in the observed kinetic regime.

For Cu-based CO_2 hydrogenation specifically, recent mechanistic studies indicate that the apparent kinetics of methanol formation are highly sensitive to the local abundance of adsorbed hydrogen and water. [119] Methanol formation has been linked to methoxy hydrolysis, and water can facilitate the CH_3O^* to CH_3OH step. [120] Accordingly, one plausible interpretation of the present data is that lowering the space velocity promotes a more water-rich and coverage-modified surface microenvironment, thereby lowering the observed Arrhenius slope for methanol formation. However, excessive space velocity shortens residence time, limiting hydrogen availability and disrupting the Cu^0–$Zn^{\delta+}$ interface, thus shifting the system toward higher-barrier pathways (e.g., carboxyl and direct CO_2 dissociation).[44] Thus, maintaining GHSV is essential to preserve low-barrier reactivity and avoid kinetic masking under transport-limited regimes. This interpretation is proposed at the level of observed kinetics and does not imply that the intrinsic elementary activation barrier of the catalyst itself is directly altered by flow rate.

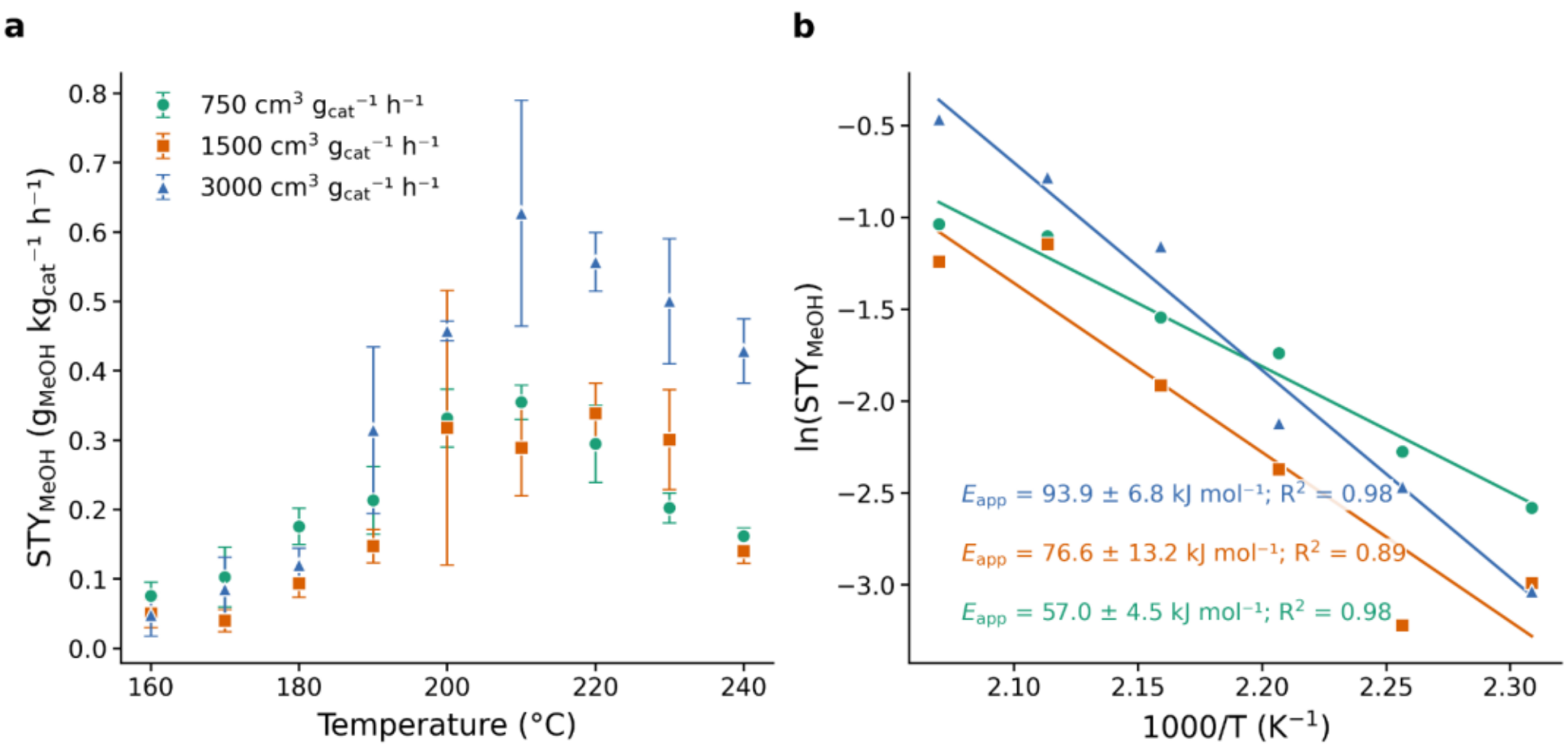

Figure 7. Experimental validation of the ML-derived GHSV–E_{app} relationship over a commercial $Cu/ZnO/Al_2O_3$ catalyst at 1 bar. **a,** Methanol space-time yield, STY_{MeOH}, as a function of reaction temperature at space velocities of 750, 1500, and 3000 $cm^3\ g_{cat}^{-1}\ h^{-1}$. Error bars represent the standard deviation of repeated measurements. **b,** Arrhenius plots of $\ln(STY_{MeOH})$ versus 1000/T with linear fits applied to the quasi-linear temperature windows used for E_{app} extraction. The fitting windows were 160 to 210 °C. The resulting methanol-based apparent activation energies are 57.0 ± 4.5, 76.6 ± 13.2, and 93.9 ± 6.8 $kJ\ mol^{-1}$, respectively.

A related practical question concerns the extent to which literature data availability and curation quality constrain the applicability of data-driven catalysis frameworks. In practice, well-studied catalytic systems remain the most accessible starting point, because the limiting factor is not the number of publications surveyed, but the number of high-quality, consistently defined data points that remain after harmonized curation. Our literature survey spans 200 publications and the model training uses 2,383 curated, permutation-invariant experimental entries. This scale is moderate for machine learning on experimental catalysis data (**Table S3** and **Fig. S23**). Across reaction classes, experimental dataset sizes span from single-digit to over ten thousand data points, indicating that datasets on the order of 10^3 are not atypical once multiple sources are harmonized. The surveyed studies cover thermocatalysis (e.g., PDH, DRM, WGS, OCM, etc.), electrocatalysis (e.g., CO_2RR, NRR, OER/HER), photocatalysis, organic catalysis, and process-related datasets. CO_2 thermocatalysis (e.g., CO_2-to-methanol and CO_2-to-olefins) is listed separately. It is worth noting that the dataset of our work is the most extensive in the CO_2-to-methanol.

To distinguish the transferability of the workflow from the availability of sufficiently curated data, we applied the same end-to-end protocol of data schema definition, curation and standardization, model training, SHAP-based interpretation, and inference to an independent electrocatalytic system, electrochemical CO_2 reduction (CO_2RR), using a curated dataset of 379 literature-derived entries. Full details of the CO_2RR dataset, preprocessing, model validation, SHAP analysis, and representative inference maps are provided in *Supplementary Note 7*. CatBoost was selected as the final model, and the α

= 0.3 setting was retained as the best accuracy–generalization compromise (**Table S4**). Interpretability analysis further showed that the selected model recovered chemically meaningful descriptor–performance relationships for the selectivity of Cu-based CO_2RR. SHAP summaries and pairwise inference maps in **Fig. S25** identify applied potential and particle size as leading descriptors and reveal output-specific, non-monotonic response surfaces for H2, CO, C1, and C2+. The categorical inference map in **Fig. S26** further shows that active phase and catalyst shape jointly redistribute the predicted product fractions.

Taken together, the targeted GHSV–E_{app} experiment and the auxiliary CO_2RR case study validate the framework at experimental corroboration within the benchmark methanol system and cross-system transferability across catalytic regimes.

Reviewers’ comments:

Reviewer #1:

Comment 1-0:

The manuscript can be accepted for publication in its current form, as the authors have clearly addressed and satisfactorily explained all comments and questions.

Response:

We thank the reviewer for their very helpful reviewing process and for recommending publication.

Reviewer #2:

Comment 2-0:

I am fully satisfied with the current version of the manuscript and recommend it for acceptance.

Response:

We thank the reviewer for their positive assessment and for recommending acceptance.

Reviewer #3:

Comment 3-0:

Thank you very much for the detailed rebuttal and the substantial revisions to the manuscript. The authors have addressed most of the previously raised concerns satisfactorily. A few points remain, which are outlined below. The comment numbering follows the original numbering to avoid confusion.

Response:

We thank the reviewer for their endorsement of this manuscript. Below we address each of the reviewer’s comments interleaved with our responses to the referee.

Comment 3-4 (related to Comment 3-1):

Thank you for the detailed response and the additional case study. While the inclusion of a smaller dataset is appreciated, both examples still rely on well established catalytic systems supported by decades of accumulated literature data. Importantly, this should not be seen as a weakness, but rather as a key strength that is currently underemphasized in the manuscript. In its present form, the storyline aligns well with a catalysis focused journal, whereas the connection to engineering practice, particularly expected for Communications Engineering, remains less clearly articulated. For example, the motivation in the abstract ([Presently, the optimization of

heterogeneous catalysis relies on navigating a complex parameter space through intuition-guided, resource-intensive experimentation]) would benefit from a clearer engineering context. The authors are therefore encouraged to more explicitly position their work within an engineering framework, highlighting its relevance for industrial applications such as identifying improved operating conditions, enhancing process efficiency, or enabling cost reductions in established catalytic systems. In this context, it would be particularly valuable to identify and discuss existing gaps between current industrial practice and data-driven optimization approaches, supported by appropriate references. This perspective should be consistently reflected in the Abstract, Introduction, and Conclusions. While no additional quantitative analysis is required, a clearer articulation of the practical and industrial relevance would significantly strengthen the manuscript and its suitability for the target journal.

Response:

We thank the reviewer for this constructive and helpful suggestion that the use of well established, literature-rich catalytic systems should be presented as a strength of the study rather than as a limitation. Such systems provide the accumulated experimental basis needed to evaluate how catalyst descriptors and operating variables jointly affect performance, and therefore represent a practical domain in which literature-derived machine learning can support reaction-engineering decisions.

In response, we have revised the manuscript to more explicitly position the work within an engineering framework. First, we revised the Abstract to clarify that the central problem is not only catalyst discovery, but operating-window selection in mature heterogeneous catalytic processes, where catalyst composition, preparation history, reaction conditions, productivity, selectivity, and pressure-related constraints must be considered together.

Second, we revised the Introduction so that the engineering motivation is integrated into the main storyline. Specifically, the end of the first paragraph now emphasizes that industrial-scale catalytic processes require balancing conversion, selectivity, productivity, catalyst durability, hydrogen utilization, and pressure-related process penalties. We also added a sentence in the machine learning motivation paragraph stating that mature, data-rich catalyst families should be viewed as an engineering resource, because decades of accumulated studies can provide a basis for process-relevant data-driven optimization.

Third, we clarified the gap between current practice and data-driven optimization. The revised text now states that although literature-derived ML has potential as a decision-support layer, its practical use remains limited when models rely heavily on computational data or open materials databases that do not retain experimentally reported operating conditions and operational details. This addition makes explicit why harmonized experimental datasets are needed for practical, experimentally grounded reaction optimization.

Finally, we revised the concluding part of the manuscript to better reflect the practical relevance of the framework for established catalytic technologies. We now emphasize that the framework is intended to support operating-condition prioritization, targeted validation, and process-relevant optimization.

Changes made in the manuscript:

Abstract: We revised the opening motivation to emphasize engineering optimization and operating-window selection in mature heterogeneous catalytic processes. We also revised the closing sentence to highlight operating-condition prioritization, targeted kinetic validation, and practical optimization of established catalytic technologies.

Introduction, first paragraph: We replaced the previous general statement on industrial catalytic processes with the following more engineering-focused sentence: "In this regard, combining literature-extracted data with ML bears great potential as a decision-support layer that prioritizes promising operating windows and catalyst formulations before time-intensive validation experiments. This potential is particularly relevant for mature, data-rich catalyst families, where decades of accumulated studies can serve as an engineering resource rather than merely historical background."

Introduction, machine learning motivation paragraph: We added the following sentence to clarify why mature catalytic systems are a strength for the proposed framework:
"This potential is particularly relevant for mature, data-rich catalyst families, where decades of accumulated studies can serve as an engineering resource rather than merely historical background."

Introduction, data-use gap: We revised the final sentence of the paragraph to more clearly identify the gap between current ML approaches and experimentally grounded reaction optimization:
"This incompatibility represents a fundamental impasse to ML for practical — and therefore the understanding of underpinning data-driven reaction optimization — for engineering researchers."

Comment 3-5 (related to Comment 3-2):

Thank you for the detailed response. Regarding the discussion on data curation, it would be helpful if the authors more explicitly highlight the current limitations of automated data extraction within the manuscript. In particular, a concise discussion of future perspectives, such as the potential role of large language models (LLMs) and automated summarization tools in facilitating literature data extraction, would strengthen the outlook of the study. Recent work in this direction may be briefly cited, for example https://arxiv.org/abs/2403.02901 and https://doi.org/10.1080/00219592.2024.2360928. This addition can remain concise but would provide useful context regarding scalability and future development of the framework.

Response:
We thank the reviewer for this helpful suggestion. We have added a concise statement in the Discussion to more explicitly describe the current limitations of fully automated extraction from catalysis literature.

Specifically, we now clarify that automated extraction remains challenging because catalysis papers often report key variables using heterogeneous formats, nonstandard catalyst nomenclature, graphical data, and context-dependent definitions of performance metrics. We also added a short future perspective on LLM-assisted and automated-summarization tools. The revised text notes that such tools may accelerate literature triage, table and figure identification, and preliminary record extraction, but that quantitative catalysis datasets still require human-in-the-loop verification of units, reconstructed outputs, and chemical consistency.

We have also added the reviewer-suggested references on process-oriented automatic text summarization and machine-assisted literature reviewing in heterogeneous catalysis.

Changes made in the manuscript:

We revised the Discussion as follows:
"At the data-curation stage, however, fully automated extraction remains limited by heterogeneous reporting formats, nonstandard catalyst nomenclature, graphical data, and context-dependent definitions of performance metrics. Recent progress in automatic text summarization and machine-assisted literature reviewing suggests that LLM-assisted tools could accelerate literature triage, table and figure identification, and preliminary record extraction, but quantitative catalysis datasets will still require human-in-the-loop verification of units, reconstructed outputs, and chemical consistency.[117]"
