# Supplementary material for: A machine learning perspective on three decades of methanol synthesis: research framework and experimental operation insights
Source: Commun Eng. 2026 Jun 10;5:162. doi: 10.1038/s44172-026-00706-4 (PMC13594224; doi:10.1038/s44172-026-00706-4)
Supplement: Supplementary file 2 [file 44172_2026_706_MOESM2_ESM.pdf]

Supporting Information for

A Machine Learning Perspective on Three Decades of Methanol Synthesis: Research Framework and Experimental Operation Insights

Mo Yan,[a]+ Chengxi Yao,[b]+ Shihao Wu,[c] Muhammad Asif,[d] Nuning Anugrah Putri Namari,[a] Joohwan Ha,[e] Ziyang Wang,[b] Jaewon Lee,[b] Dayoung Yu,[f] Seokchan Lee,[f] Seungjae Lee,[b] Hao Yu,[g] Xiaoming Duan,*[c] Taesung Kim*[b,e,f]

Email: duanxiaoming@hit.edu.cn; tkim@skku.edu

The Supporting Information includes:

Supplementary Notes 1-8
Figs. S1 to S28
Tables S1 to S5

Supplementary Note 1. Positioning of the present work

Table S1. Positioning of the present work relative to representative machine-learning studies relevant to CO_2-to-methanol catalysis

**Representative study**	**Data basis**	**Catalyst scope**	**Dataset size**	**Model inputs**	**Model outputs**	**Difference**
Bhardwaj et al. (2023)[1]	Experimental literature	Cu-based catalysts in direct CO_2 hydrogenation	227	Reaction conditions + selected catalyst textural descriptors	X_{CO2}, S_{MeOH}, S_{CO}, STY_{MeOH}	Closest in chemistry, but substantially smaller dataset and no explicit synthesis-history curation or literature-derived kinetic subset
Suvarna et al. (2022)[2]	Experimental literature	Cu-, Pd-, In2O3-, and ZnO-ZrO2-based catalysts	1425 compiled / 1201 modeled	Composition + synthesis conditions + reaction conditions	STY_{MeOH}	Broad cross-family framework centered on a single target; not Cu/ZnO-benchmark-focused and no kinetic extension
Vanjari et al. (2023)[3]	Experimental literature	Diverse catalysts for direct CO_2 hydrogenation to methanol	698	Catalyst composition + preparation conditions + operating parameters	X_{CO2}, S_{MeOH}	Multi-catalyst framework with two outputs only; no joint S_{CO}/STY_{MeOH} analysis and no kinetic extension
Ghafarian Nia et al. (2025)[4]	Experimental literature	Catalytic hydrogenation to methanol over diverse catalysts	1765	Operating parameters + catalyst loading + surface area + XRD-derived crystal size/crystallinity	X_{CO2}, S_{MeOH}, S_{CO}	Broader process-oriented framework including optimization, techno-economic analysis, and LCA; not centered on the Cu/ZnO benchmark family and no literature-derived kinetic subset
Roy et al. (2021)[5]	DFT + ML high-throughput screening	Cu/Co/Ni/Zn/Mg alloy-based periodic (111) surfaces and HEA systems	Computational screening space, not a literature experimental dataset	Surface microstructure + adsorption energetics	Adsorption energies of intermediates	Computational catalyst screening rather than experimental literature integration
**Present work**	Experimental literature	Cu/ZnO-based catalysts	2551 extracted/ 2383 modeled + 940 E_{app} subset	Composition + synthesis history + operating conditions; symmetry-aware curation	X_{CO2}, S_{MeOH}, S_{CO}, STY_{MeOH} + E_{app} extension	Benchmark-family-focused literature-to-knowledge framework integrating composition, synthesis, operation, multi-output performance, and condition-dependent apparent kinetics

Supplementary Note 2. Unit conversion

Table S2. Unit conversion in dataset preprocessing.

**Parameter**	**Commonly reported units**	**Standardized unit**	**Conversion / Notes**
Ratio	molar ratio<br>weight content<br>atomic ratios	molar ratio	All retained as reported or converted accordingly
Temperature	K, °C	°C	*T*(°C) = *T* (K) − 273
Pressure	atm, MPa	bar	1 atm = 1.01325 bar; 1 MPa = 10 bar
GHSV	h^{-1},<br>$cm^3\ g_{cat}^{-1}\ h^{-1}$,<br>$NmL\ g_{cat}^{-1}\ min^{-1}$,<br>$m^3\ kg_{cat}^{-1}\ h^{-1}$,<br>$kg_{cat}\ h\ m^{-3}$,<br>$g\ \ g_{cat}^{-1}\ h^{-1}$	$cm^3\ g_{cat}^{-1}\ h^{-1}$	All values converted to volumetric hourly basis
STY_{MeOH}	$mol_{MeOH}\ g_{cat}^{-1}\ h^{-1}$,<br>$mmol_{MeOH}\ g_{cat}^{-1}\ s^{-1}$,<br>$mL_{MeOH}\ g_{cat}^{-1}\ h^{-1}$,<br>$g_{MeOH}\ g_{cat}^{-1}\ h^{-1}$,	$g_{MeOH}\ kg_{cat}^{-1}\ h^{-1}$	Standardized using methanol molar mass (32.04 g mol^{-1}) and gas molar volume (22.4 L mol^{-1})

For studies that did not explicitly report the methanol space-time yield ($\mathrm{STY_{MeOH}}$), we calculated it using the reported CO_2 conversion ($\mathrm{X_{CO_2}}$), methanol selectivity ($\mathrm{S_{MeOH}}$), and CO_2 gas hourly space velocity ($\mathrm{GHSV_{CO_2}}$):

$$\mathrm{STY}(\mathrm{g_{MeOH}\ kg_{cat}^{-1}\ h^{-1}}) = \frac{\mathrm{GHSV_{CO_2}} \times \mathrm{X_{CO_2}} \times \mathrm{S_{MeOH}} \times M_{\mathrm{MeOH}}}{22.4} \qquad \text{(S1)}$$

where $M_{\mathrm{MeOH}} = 32.04\ \mathrm{g\ mol^{-1}}$ is the molar mass of methanol. When $\mathrm{GHSV_{CO_2}}$ was not directly available, it was estimated by multiplying the total GHSV by the molar fraction of CO_2 in the feed gas mixture. This approach enabled consistent derivation of STY values across the dataset, even when not explicitly reported in the original sources.

Conversely, in cases where CO_2 conversion was not reported but $\mathrm{STY_{MeOH}}$ and $\mathrm{S_{MeOH}}$ were available, we rearranged Equation (S1) to calculate CO_2 conversion:

$$\mathrm{X_{CO_2}} = \frac{\mathrm{STY\ (g_{MeOH}\ kg_{cat}^{-1}\ h^{-1})} \times 22.4}{\mathrm{GHSV_{CO_2}} \times \mathrm{S_{MeOH}} \times \mathrm{M_{MeOH}}} \qquad \text{(S2)}$$

This reciprocal approach allowed us to consistently reconstruct missing conversion or productivity values and maximize the usable data points in the final dataset.

Supplementary Note 3. Missing values and outliers

The compiled raw dataset inherently contained missing values for certain features in some entries (for instance, if a particular study did not report catalyst surface area or the exact H_2:CO_2 ratio used). Rather than discard all such entries, which would severely shrink the dataset, we employed strategies to handle missing data. After excluding records with non-reconstructable key performance outputs, the remaining descriptor-level missingness was sparse and was handled without removing additional entries. Because these missing values originated from different literature reports rather than ordered experimental sequences, interpolation was not applied. Instead, continuous descriptors were imputed using a kernel-density-estimation-based stochastic sampling procedure fitted to the observed values of each descriptor, whereas categorical descriptors were imputed by sampling from their empirical frequency distributions. Promoter-ratio descriptors were treated separately: entries explicitly reported as “Null” were assigned a ratio of zero, while missing ratios associated with non-null promoters were imputed from promoter-specific observed distributions when available, with fallback to the global observed ratio pool. Where necessary, sampled values were constrained to physically meaningful ranges. Full implementation details are provided in the accompanying preprocessing code.

We also checked for outliers or inconsistent data points. Outliers, such as unusually high reported STY_{MeOH} or CO_2 conversion values that were inconsistent with the given reaction conditions, were cross-validated against original sources. In rare cases where data appeared erroneous or not directly comparable (e.g., different definitions or steady-state not reached), such entries were excluded.

As for the ratio of promoter to Cu, values exceeding a critical threshold (set at 20) were interpreted as indicative of support materials and labeled with “/S.” Similarly, when the third component in a catalyst system was explicitly designated as a support (e.g., SiO_2), we denoted it accordingly (e.g., “SiO_2/S”).

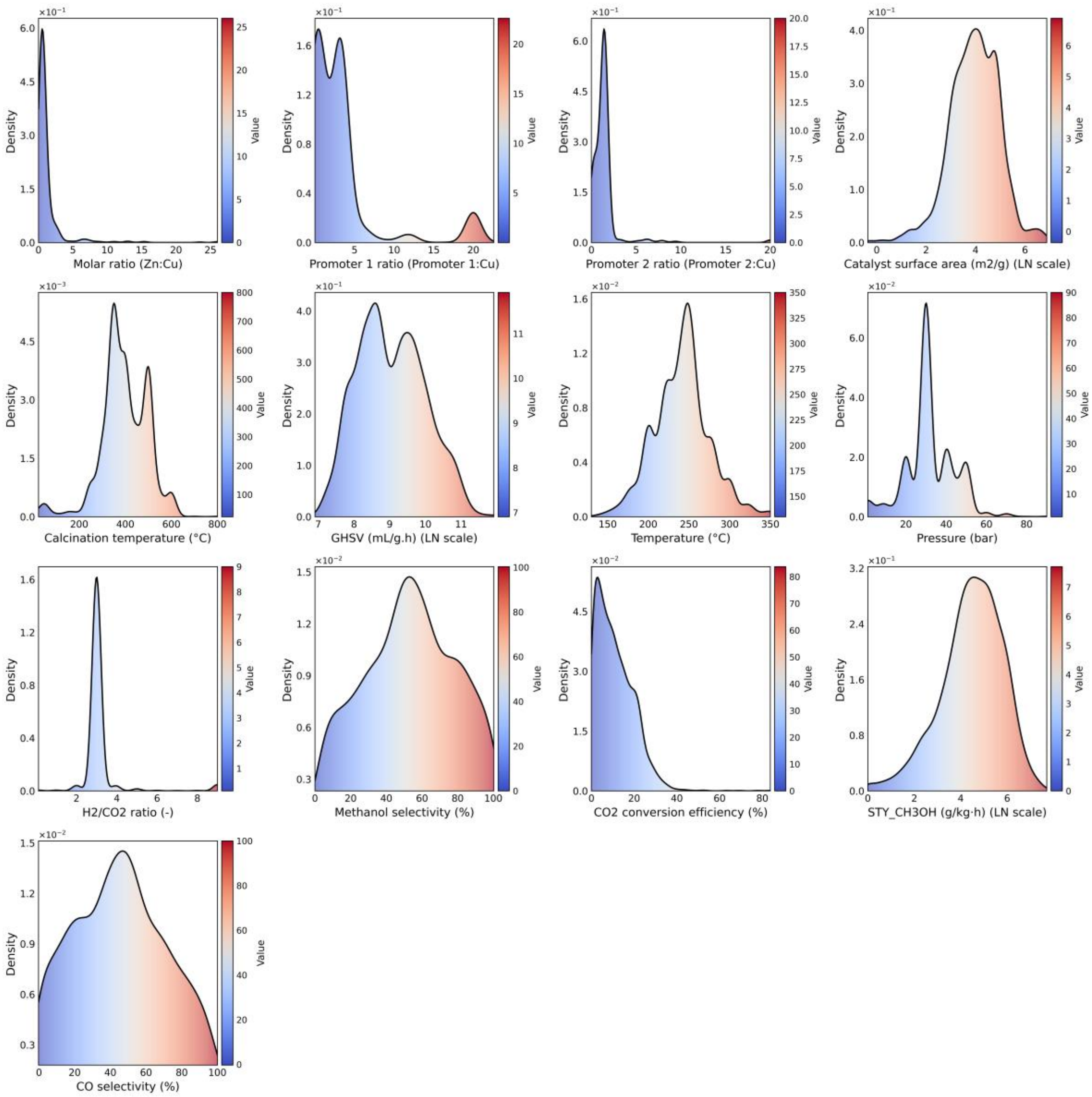

Figure S1. Distributions of selected numerical features after preprocessing. Several descriptors, (catalyst surface area, GHSV, and STY_{MeOH}) were transformed using a natural logarithm scaling followed by standardization, resulting in more symmetric distributions better suited for machine learning and improved numerical stability.

Supplementary Note 4. Activation energy subset construction

To complement the main dataset with kinetic information, we constructed a dedicated subset of apparent activation energies for methanol formation, E_{app}, derived from temperature-dependent methanol formation rates reported in the literature. Two routes were used:

(i) Directly reported values. When a publication explicitly reported an apparent activation energy (or an Arrhenius slope) for methanol formation under specified reaction conditions, the value was extracted together with the corresponding catalyst descriptors (composition, promoters, synthesis descriptors) and nominal operating parameters (total pressure, feed composition or nominal partial pressures, H_2/CO_2 ratio, and GHSV).

(ii) Regenerated values from temperature-series rate data. When E_{app} was not reported, we regenerated it by fitting a temperature series of methanol formation rate proxies (most commonly $\mathrm{STY}_{\mathrm{MeOH}}$) obtained under otherwise identical catalyst identity and nominal reaction conditions. Numerical values were taken from tables/text when available, or digitized from plots using WebPlotDigitizer as described in Supplementary Note 2. For consistency, $\mathrm{STY}_{\mathrm{MeOH}}$ values were converted to a unified rate proxy r ($\mathrm{g_{MeOH}\ kg_{cat}^{-1}\ h^{-1}}$).

Each temperature series was then fitted to the Arrhenius form:

$$\ln r \;=\; \ln A_{\mathrm{app}} \;-\; \frac{E_{\mathrm{app}}}{RT} \qquad \text{(S3)}$$

where R is the universal gas constant (8.314 J mol^{-1} K^{-1}) and T is the absolute temperature (K). The apparent activation energy was obtained as $E_{\mathrm{app}} = -R \times$ slope from an ordinary least-squares regression of $\ln r$ versus $1/T$.

To ensure that each fitted E_{app} corresponds to a well-defined experimental window, only temperature series meeting all of the following criteria were retained: (1) At least three distinct temperatures within the series; (2) Identical catalyst identity and pretreatment as described in the source; (3) Identical nominal operating conditions within the series (total pressure, feed composition or nominal partial pressures, and GHSV/residence-time setting as reported); (4) Arrhenius linearity threshold of $R^2 > 0.85$; (5) A rationality test was conducted by manual inspection to eliminate obvious digital errors, unit inconsistencies, and non-physical fits (e.g., highly abnormal slopes/intercepts that are inconsistent with the underlying data trends).

This fitting and filtering procedure yielded 964 E_{app} entries. Finally, to enforce permutation invariance for unordered promoter pairs, we applied the same odd-cycle conflict resolution

and canonicalization procedure used for the main dataset. After removing odd-cycle conflicts and redundant symmetric entries, 940 valid E_{app} data points remained and were used for model training.

Supplementary Note 5. Metrics of the ML models

We evaluated six models, and the hyperparameters of each model were tuned to optimize predictive performance. The performance of each model was evaluated by residual (r_i), coefficient of determination (R^2), mean squared error (MSE), and mean absolute error (MAE), which are defined as

$$r_i = y_i - \hat{y}_i \tag{S4}$$

$$R^2 = 1 - \frac{\sum_{i=1}^{n}(y_i - \hat{y}_i)^2}{\sum_{i=1}^{n}(y_i - \bar{y})^2} \tag{S5}$$

$$\mathrm{MSE} = \frac{1}{n}\sum_{i=1}^{n}(y_i - \hat{y}_i)^2 \tag{S6}$$

$$\mathrm{MAE} = \frac{1}{n}\sum_{i=1}^{n}|y_i - \hat{y}_i| \tag{S7}$$

where y_i indicates the values of the data collected from the literature, $\hat{y}_i$ indicates the result predicted by the ML models, and $\bar{y}$ indicates the average value of the literature data. Generally, a better model performance should have R^2 value close to 1, and a small MSE and MAE close to 0.

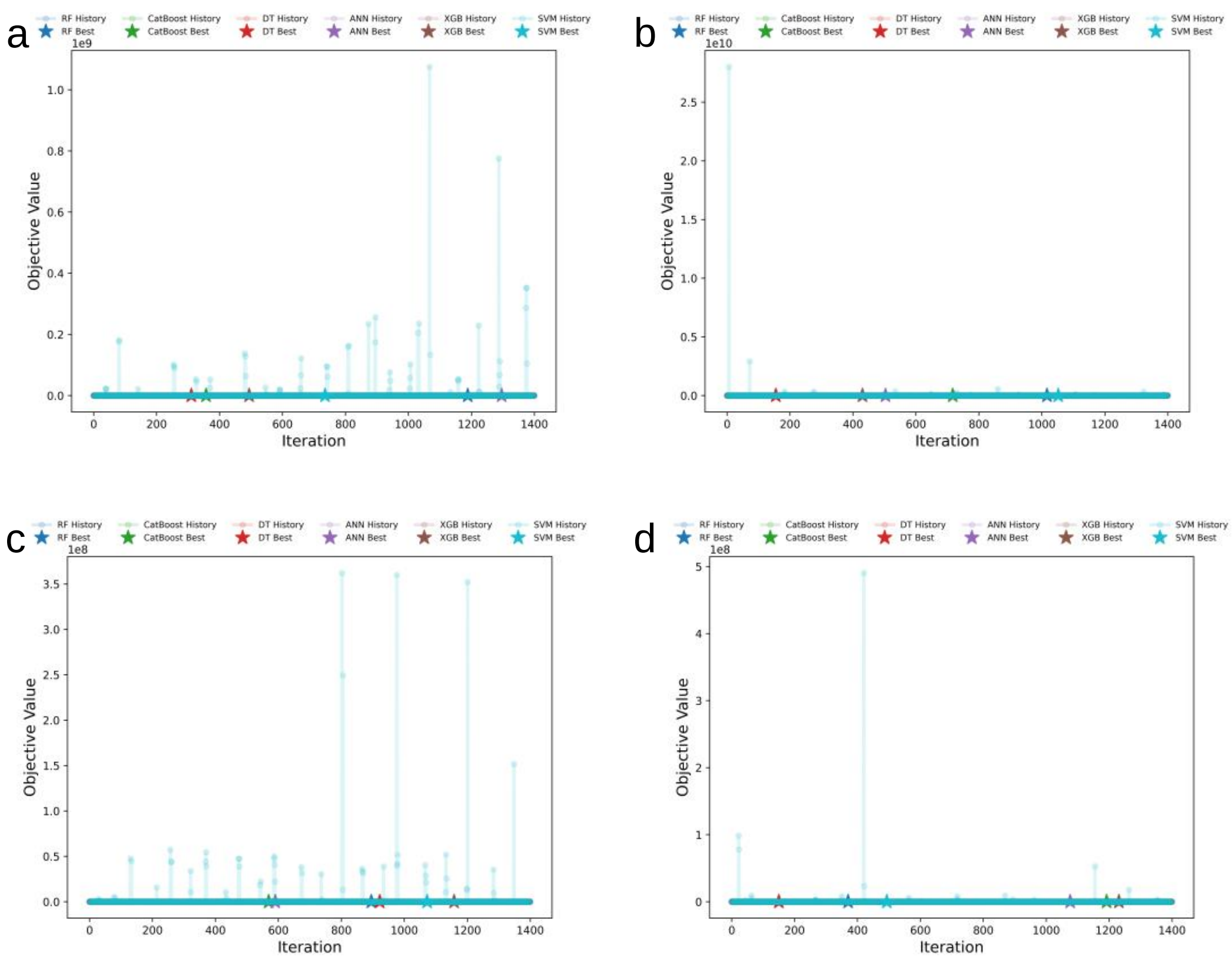

Figure S2. Bayesian hyperparameter optimization trajectories for six models across different α values, (a) α = 0, (b) α = 0.01, (c) α = 0.03, (d) α = 0.07. Each point represents the value of the objective function of Equation (1) in the main text from a sampled hyperparameter configuration during Bayesian optimization. The trajectories illustrate the search history (dots) and best-performing configurations (stars) for RF, XGB, DT, ANN, CatBoost, and SVM.

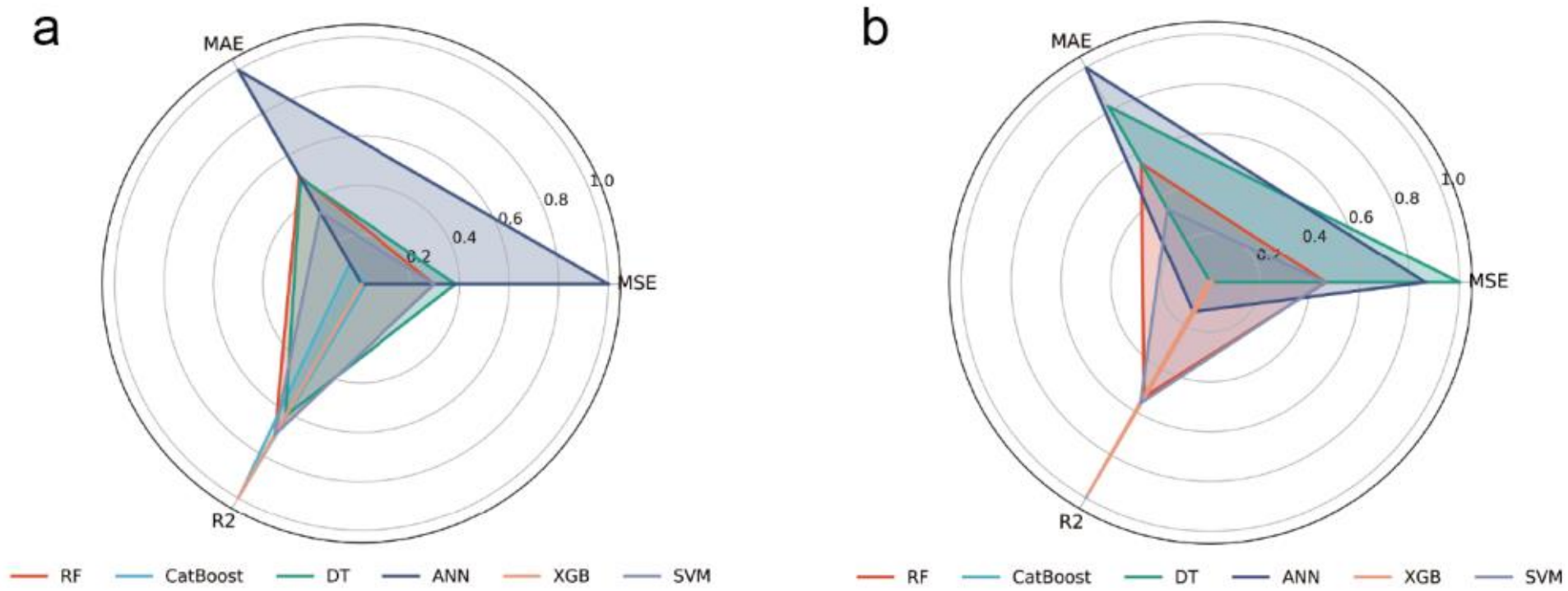

Figure S3. Radar plots comparing model performance across MAE, MSE, and R^2 metrics for the six ML algorithms (RF, CatBoost, DT, ANN, XGB, SVM) at α = 0. (a) Training set results; (b) Validation set results.

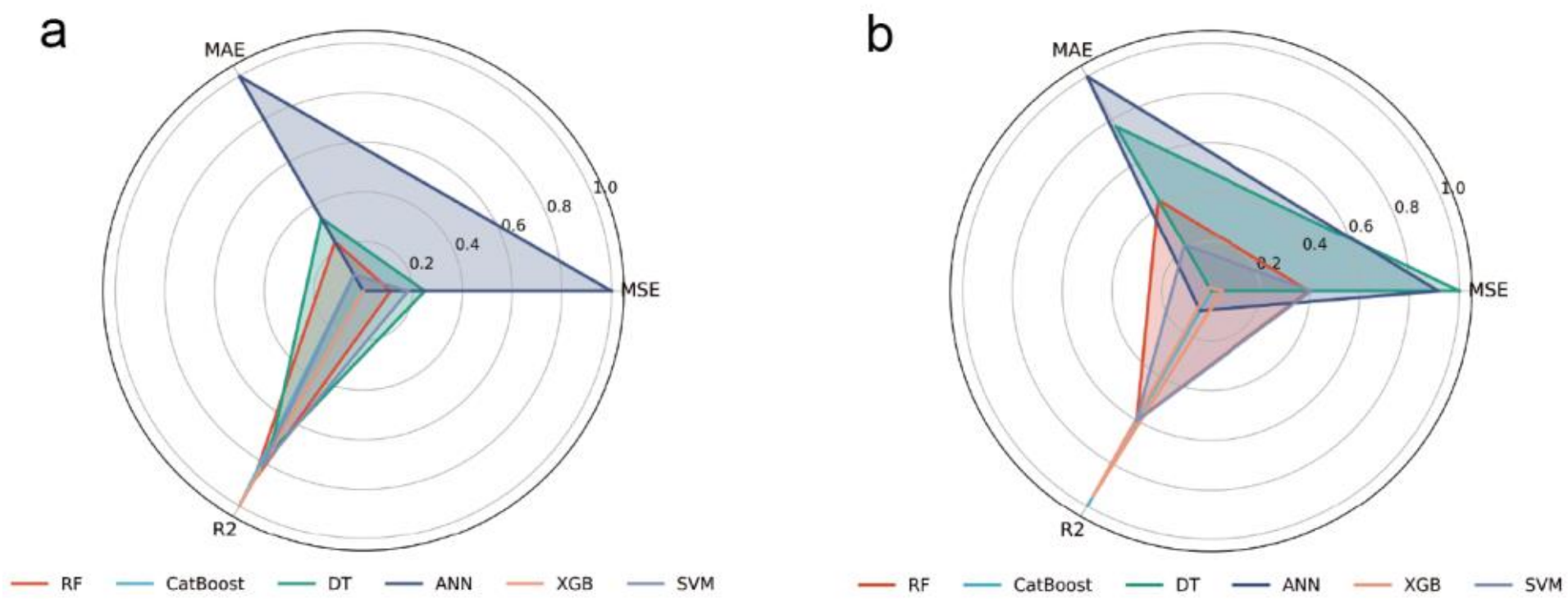

Figure S4. Radar plots comparing model performance across MAE, MSE, and R^2 metrics for the six ML algorithms (RF, CatBoost, DT, ANN, XGB, SVM) at α = 0.01. (a) Training set results; (b) Validation set results.

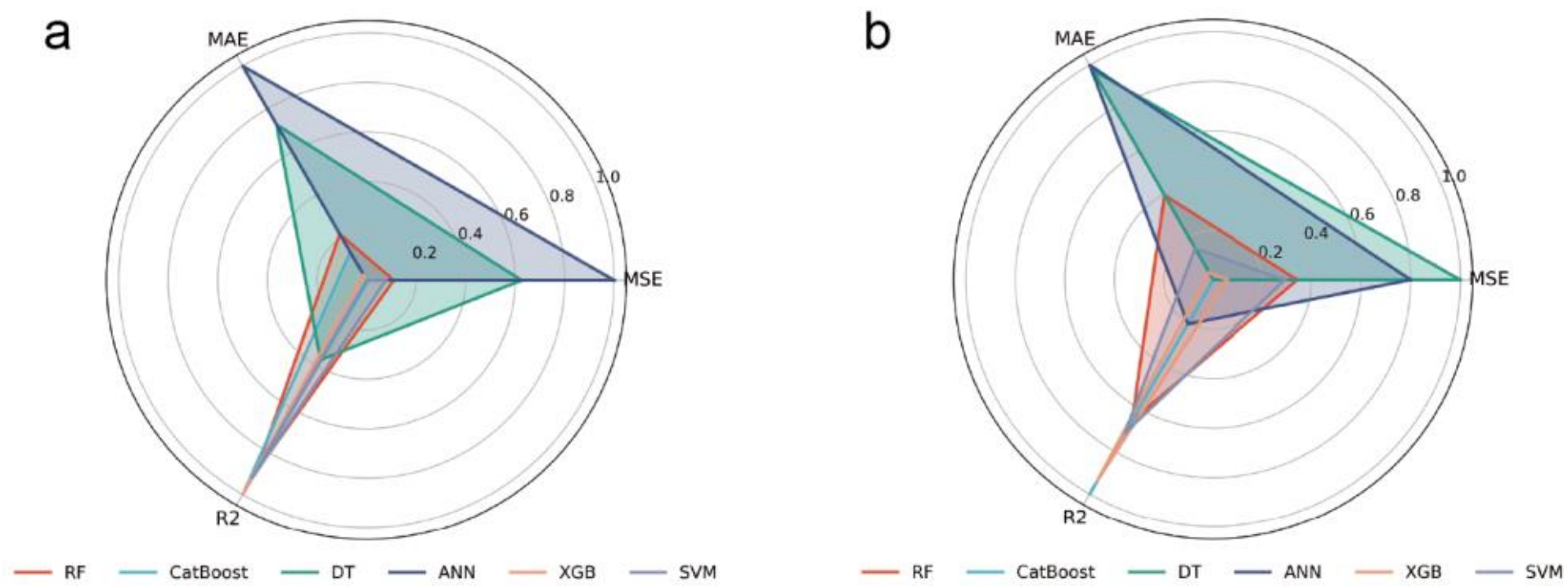

Figure S5. Radar plots comparing model performance across MAE, MSE, and R^2 metrics for the six ML algorithms (RF, CatBoost, DT, ANN, XGB, SVM) at α = 0.03. (a) Training set results; (b) Validation set results.

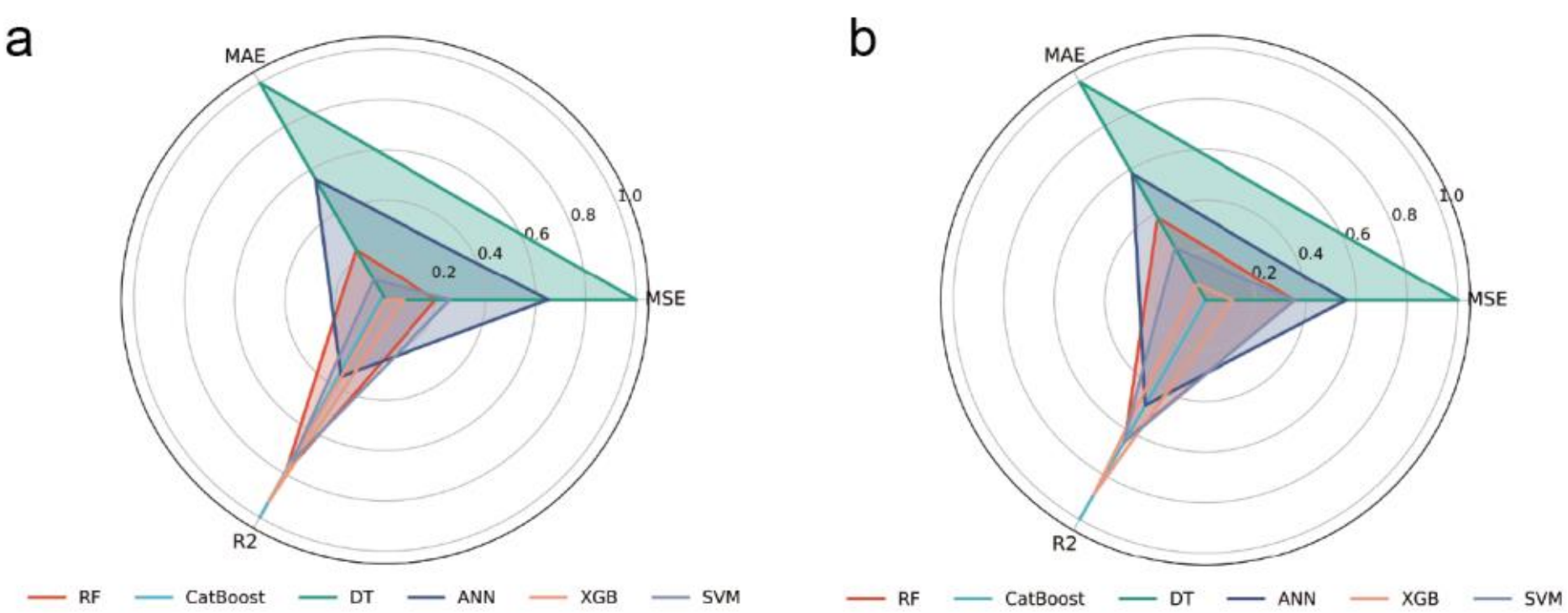

Figure S6. Radar plots comparing model performance across MAE, MSE, and R^2 metrics for the six ML algorithms (RF, CatBoost, DT, ANN, XGB, SVM) at α = 0.07. (a) Training set results; (b) Validation set results.

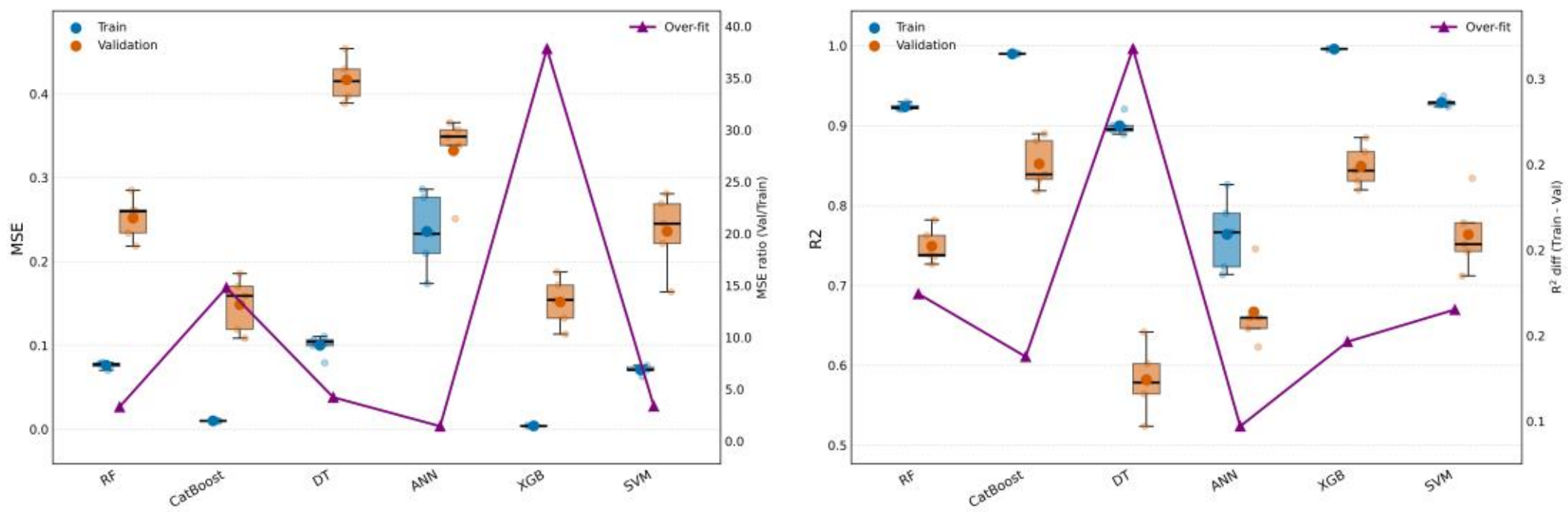

Figure S7. Fold-averaged metrics of MSE, R^2 and overfitting index of the six models at α = 0.

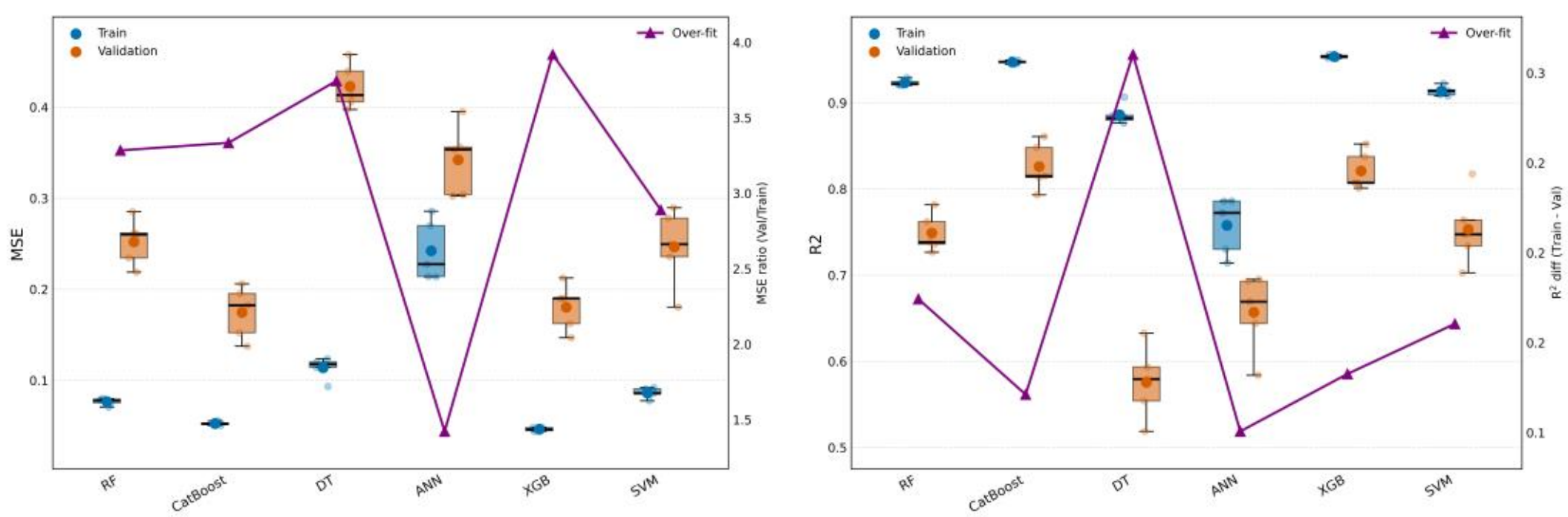

Figure S8. Fold-averaged metrics of MSE, R^2 and overfitting index of the six models at α = 0.01.

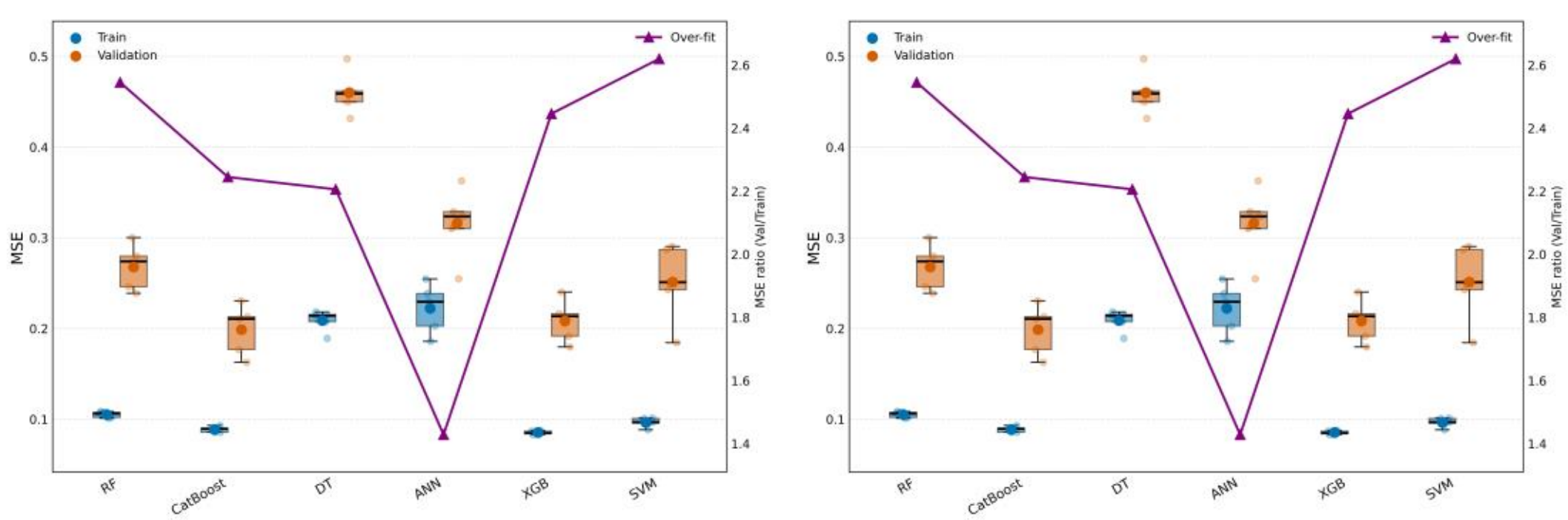

Figure S9. Fold-averaged metrics of MSE, R^2 and overfitting index of the six models at α = 0.03.

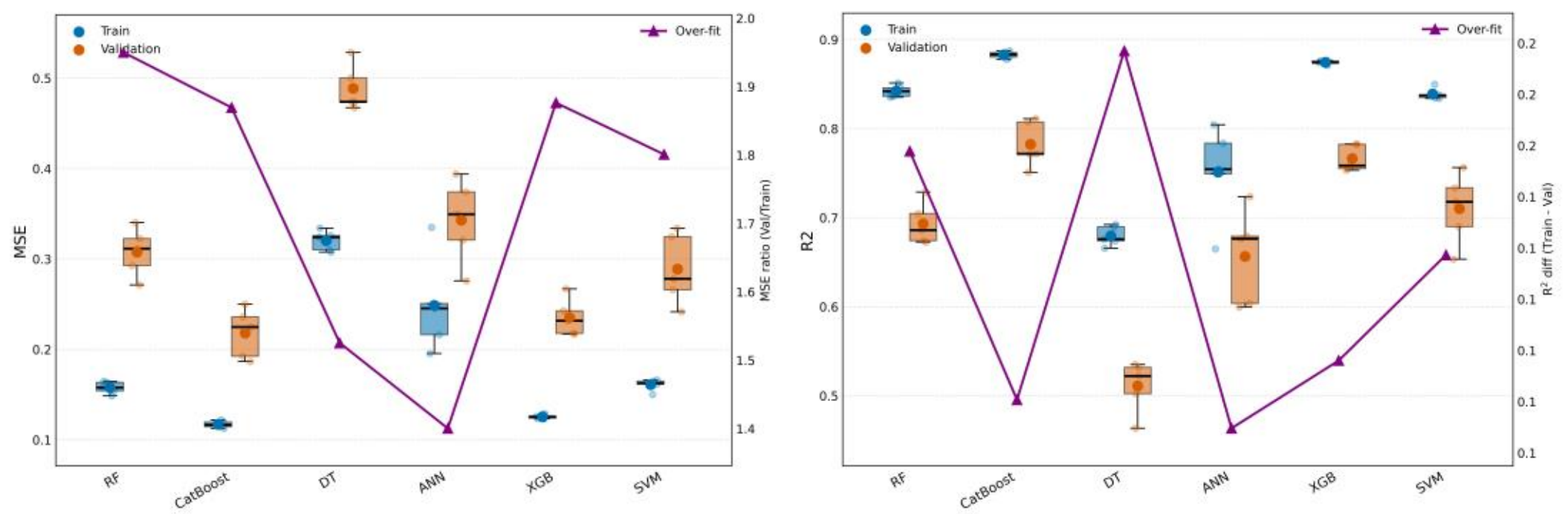

Figure S10. Fold-averaged metrics of MSE, R^2 and overfitting index of the six models at $\alpha = 0.07$.

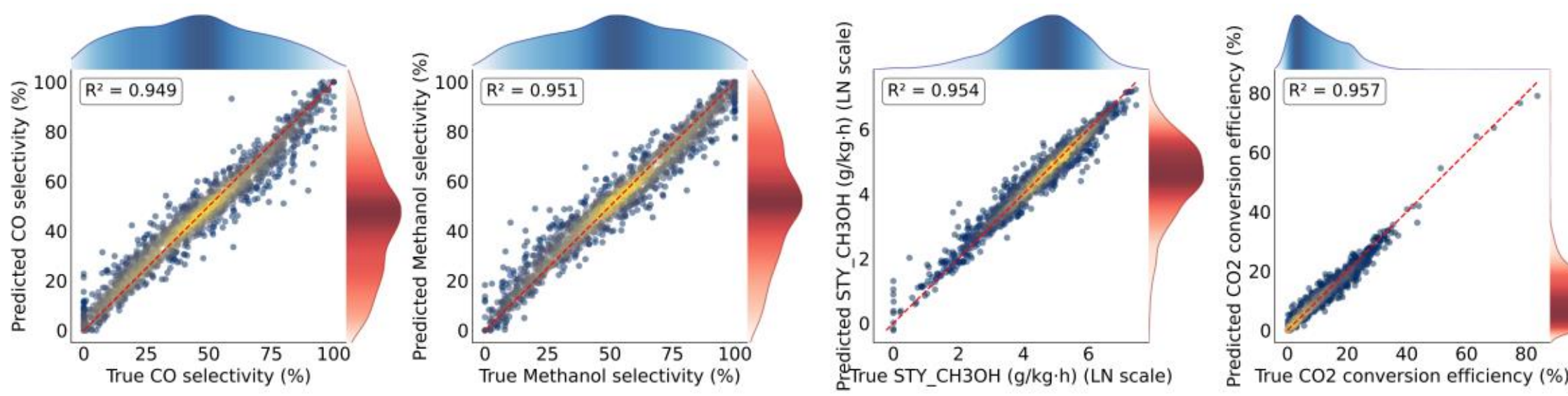

Figure S11. Parity plots for the CatBoost model (with optimal regularization, $\alpha = 0.01$) evaluated on the training set. The plots display the predicted versus experimental values for (from left to right): S_{CO}, S_{MeOH}, STY_{MeOH}, and X_{CO2}.

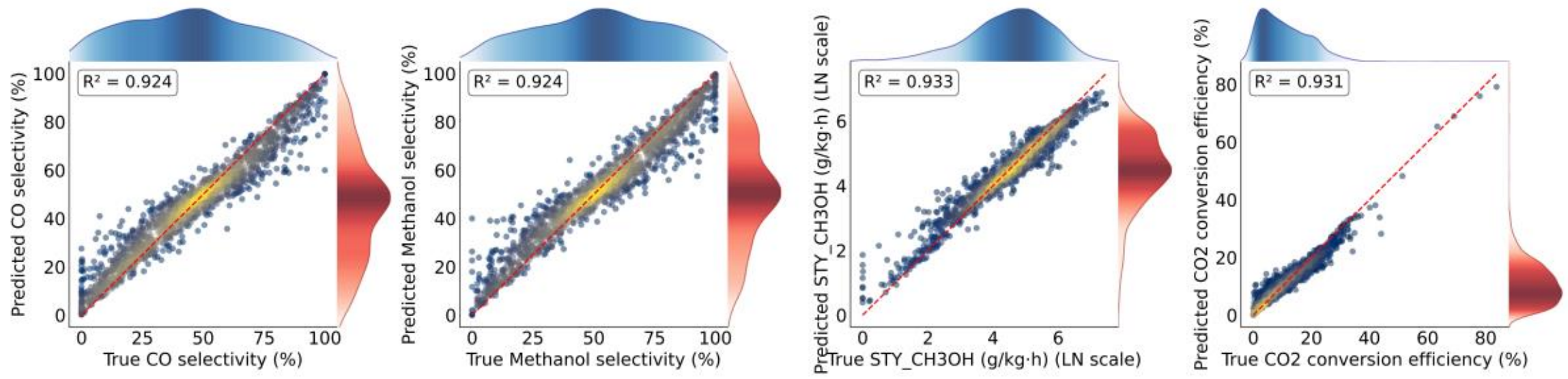

Figure S12. Parity plots for the RF model (with optimal regularization, α = 0.00) evaluated on the training set. The plots display the predicted versus experimental values for (from left to right): S_{CO}, S_{MeOH}, STY_{MeOH}, and X_{CO2}.

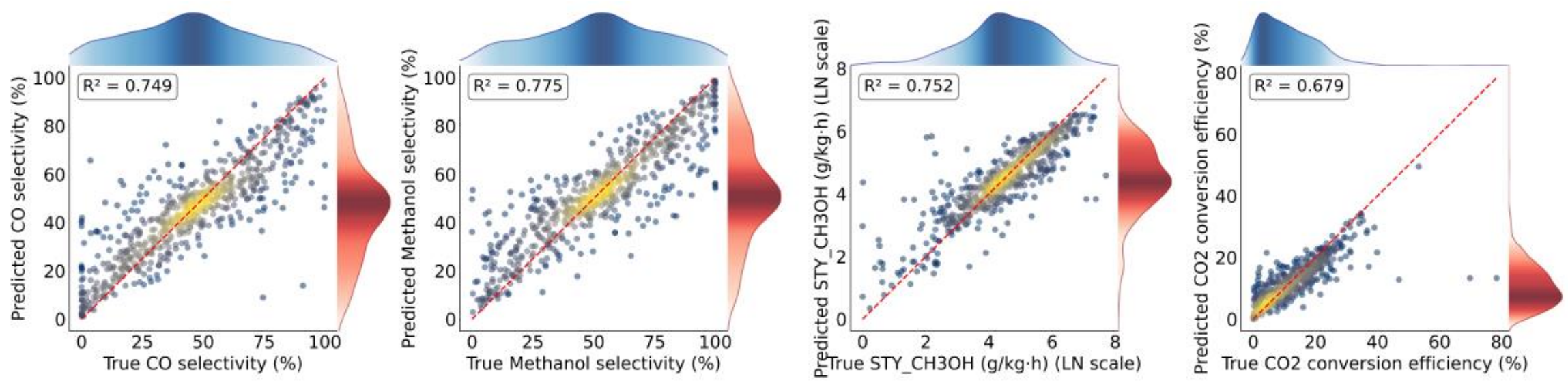

Figure S13. Parity plots for the RF model (with optimal regularization, α = 0.00) evaluated on the validation set. The plots display the predicted versus experimental values for (from left to right): S_{CO}, S_{MeOH}, STY_{MeOH}, and X_{CO2}.

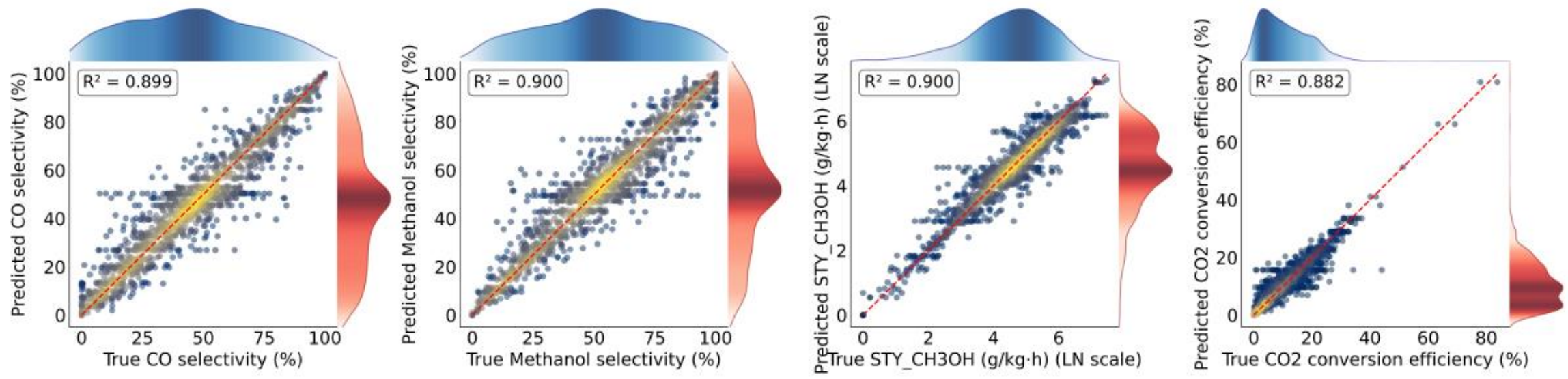

Figure S14. Parity plots for the DT model (with optimal regularization, α = 0.01) evaluated on the training set. The plots display the predicted versus experimental values for (from left to right): S_{CO}, S_{MeOH}, STY_{MeOH}, and X_{CO2}.

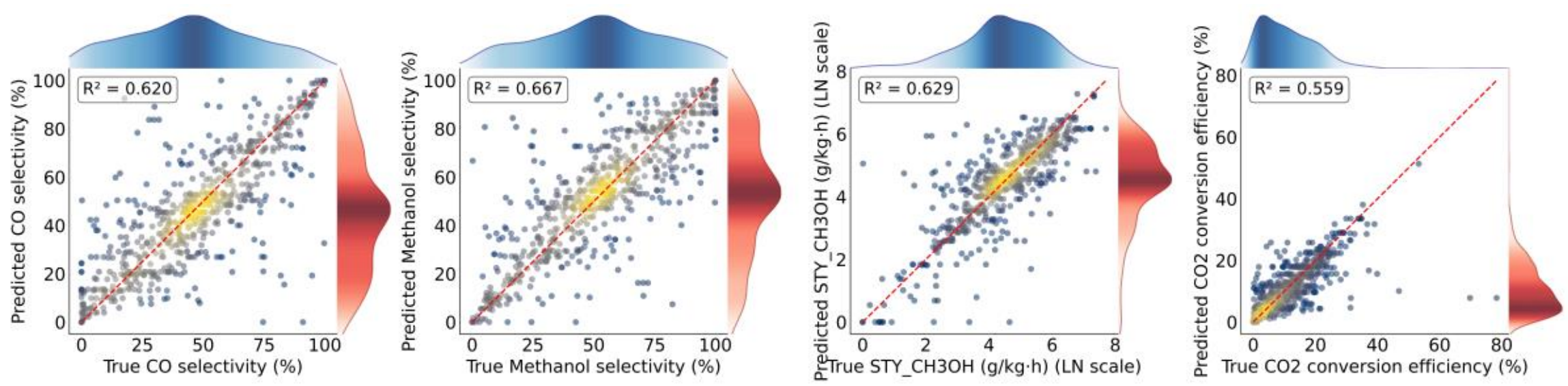

Figure S15. Parity plots for the DT model (with optimal regularization, α = 0.01) evaluated on the validation set. The plots display the predicted versus experimental values for (from left to right): S_{CO}, S_{MeOH}, STY_{MeOH}, and X_{CO2}.

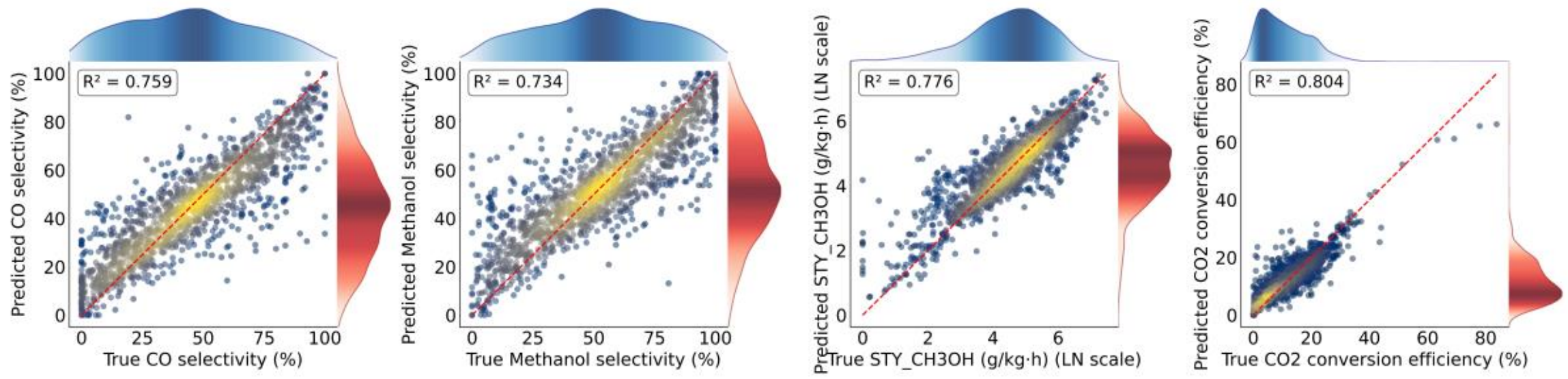

Figure S16. Parity plots for the ANN model (with optimal regularization, α = 0.00) evaluated on the training set. The plots display the predicted versus experimental values for (from left to right): S_{CO}, S_{MeOH}, STY_{MeOH}, and X_{CO2}.

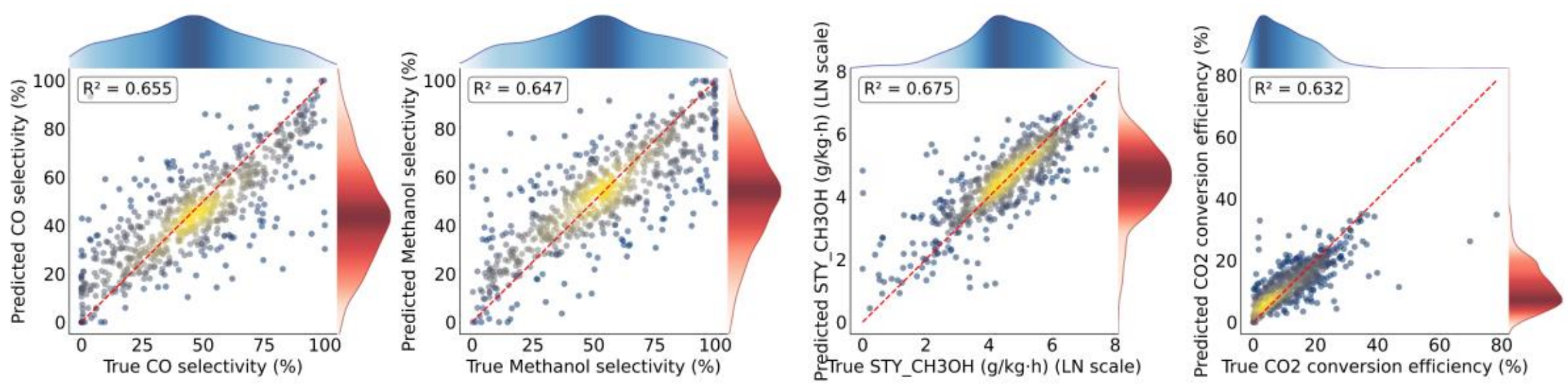

Figure S17. Parity plots for the ANN model (with optimal regularization, α = 0.00) evaluated on the validation set. The plots display the predicted versus experimental values for (from left to right): S_{CO}, S_{MeOH}, STY_{MeOH}, and X_{CO2}.

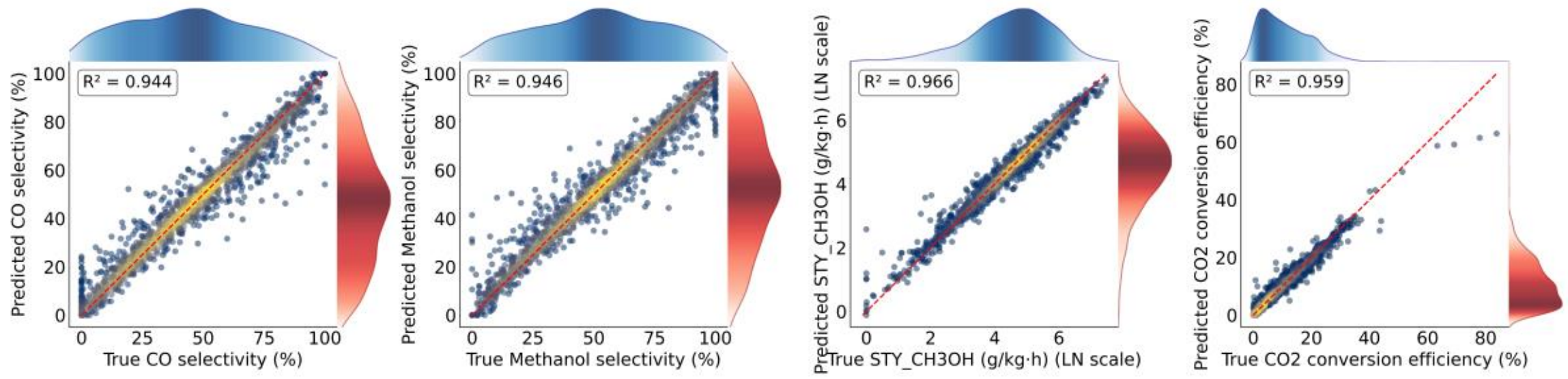

Figure S18. Parity plots for the XGB model (with optimal regularization, α = 0.01) evaluated on the training set. The plots display the predicted versus experimental values for (from left to right): S_{CO}, S_{MeOH}, STY_{MeOH}, and X_{CO2}.

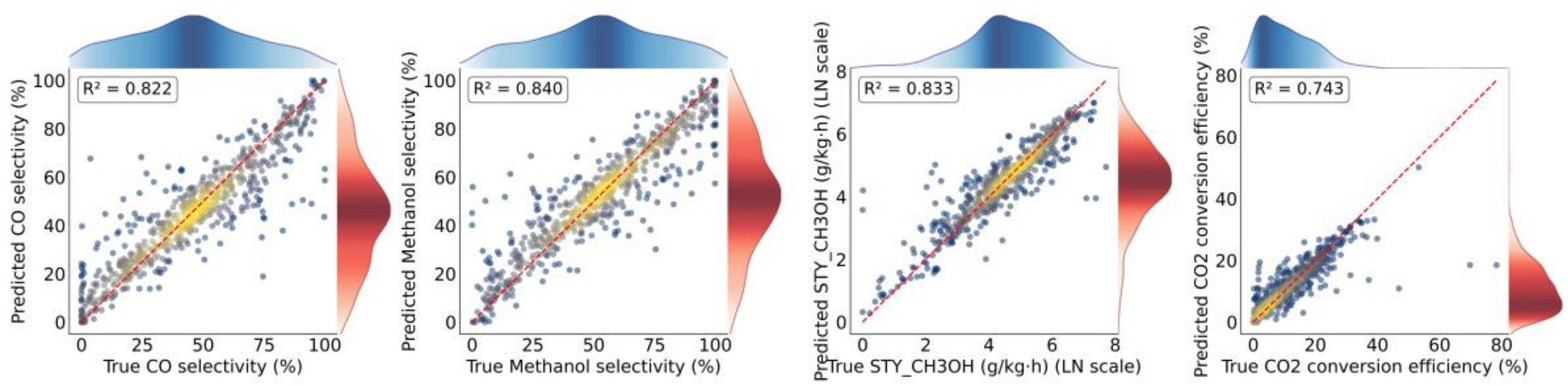

Figure S19. Parity plots for the XGB model (with optimal regularization, α = 0.01) evaluated on the validation set. The plots display the predicted versus experimental values for (from left to right): S_{CO}, S_{MeOH}, STY_{MeOH}, and X_{CO2}.

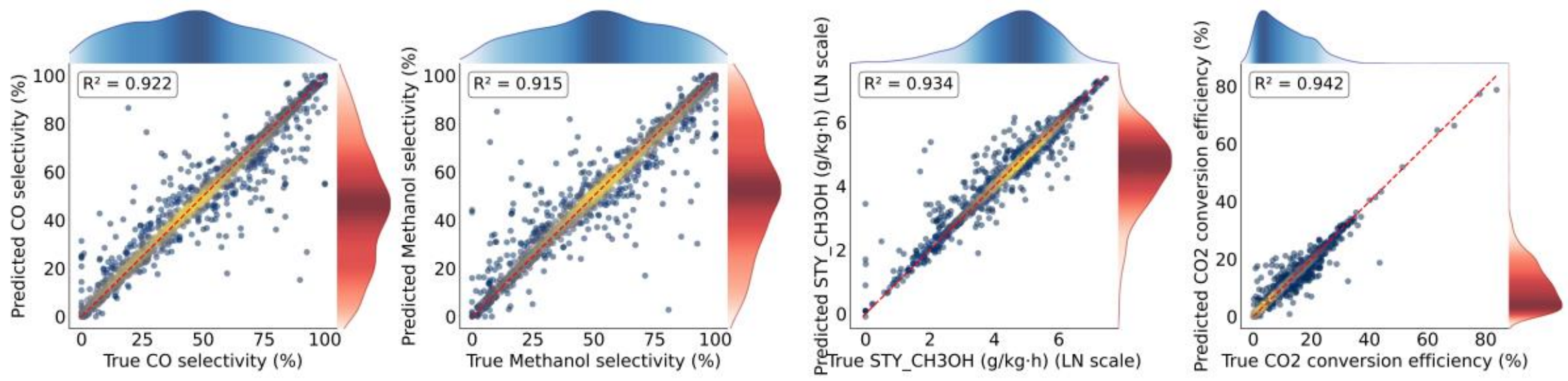

Figure S20. Parity plots for the SVM model (with optimal regularization, α = 0.00) evaluated on the training set. The plots display the predicted versus experimental values for (from left to right): S_{CO}, S_{MeOH}, STY_{MeOH}, and X_{CO2}.

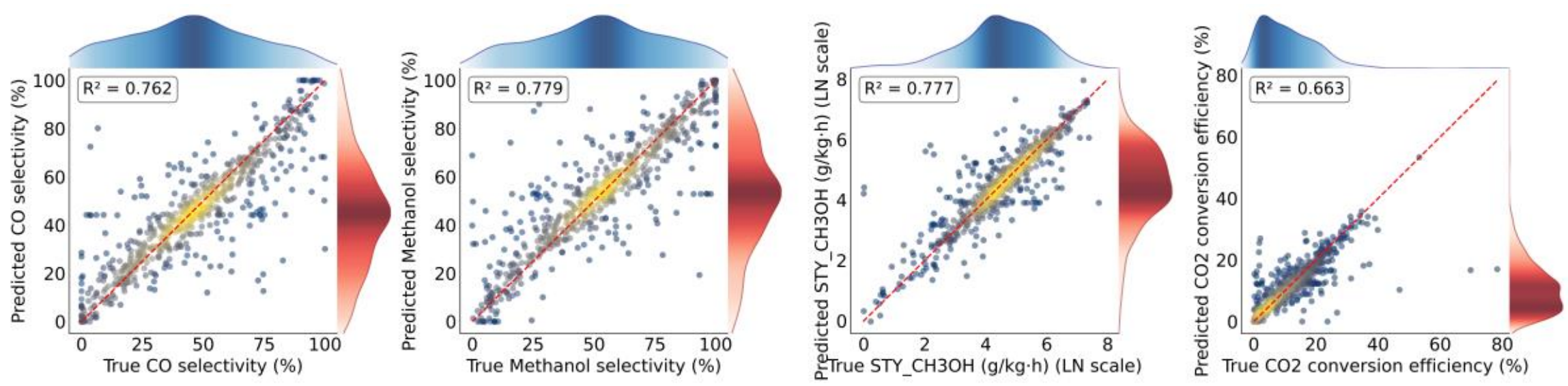

Figure S21. Parity plots for the SVM model (with optimal regularization, α = 0.00) evaluated on the validation set. The plots display the predicted versus experimental values for (from left to right): S_{CO}, S_{MeOH}, STY_{MeOH}, and X_{CO2}.

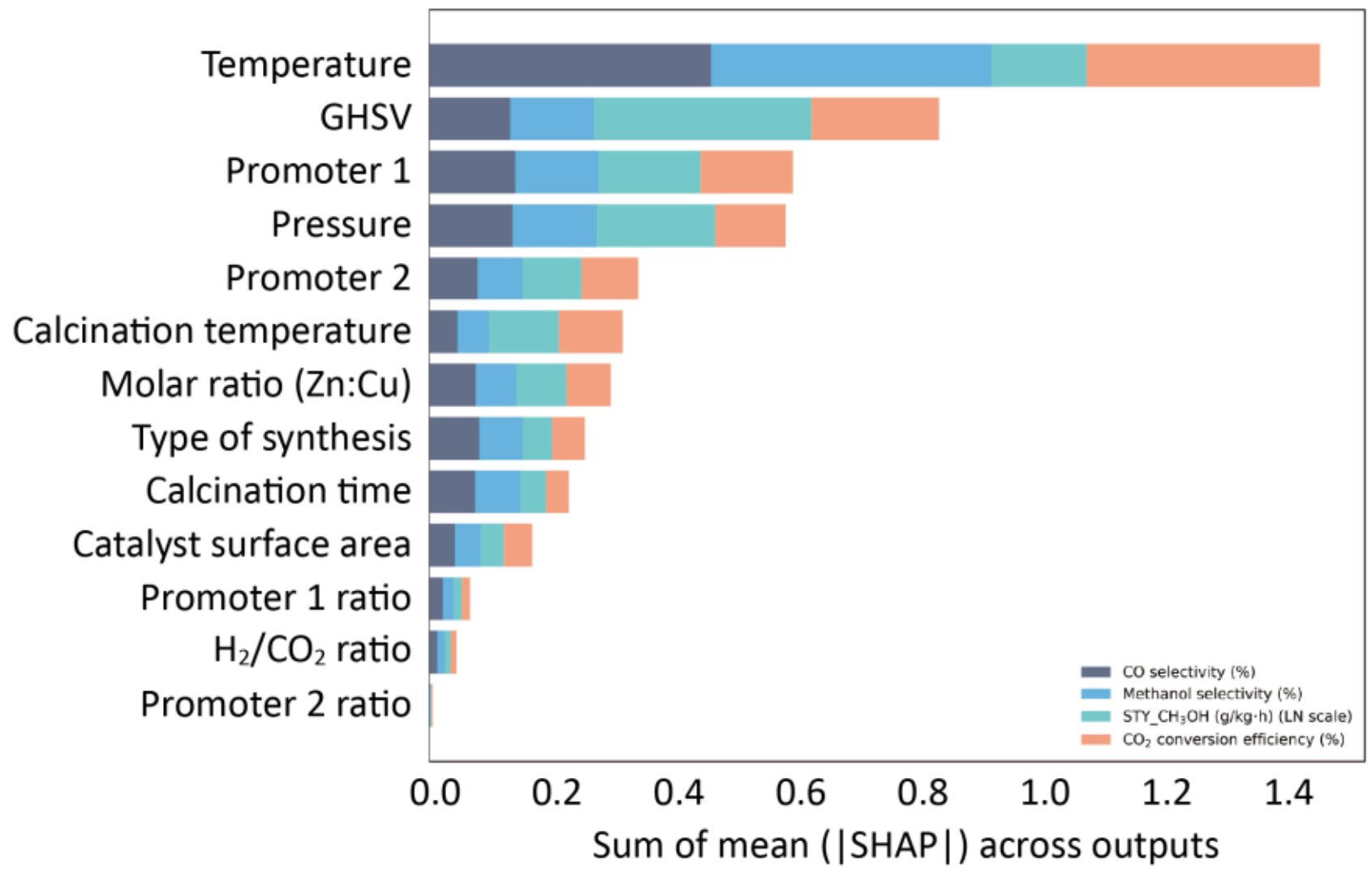

Figure S22. Global feature importance analysis for normalized SHAP values of the trained optimal CatBoost model for the summation of the four output targets.

Table S3. Model performance and overfitting metrics for the ML models trained at varying regularization strengths (α) for E_{app} dataset.

Model	α	Train		Valid		Overfitting		CV (R^2)
		R^2	MSE	R^2	MSE	MSE Ratio	R^2 difference	
RF	0	0.939	0.0613	0.826	0.1735	2.83	0.11	0.86
	0.001	0.939	0.0608	0.827	0.1725	2.84	0.11	0.86
	0.003	0.939	0.0609	0.827	0.1729	2.84	0.11	0.86
	0.005	0.939	0.0608	0.827	0.1725	2.84	0.11	0.86
	0.007	0.939	0.0608	0.826	0.1730	2.84	0.11	0.86
CatBoost	0	0.980	0.0202	0.918	0.0813	4.02	0.06	0.92
	**0.001**	**0.980**	**0.0201**	**0.922**	**0.0777**	**3.87**	**0.06**	**0.91**
	0.003	0.975	0.0249	0.913	0.0868	3.49	0.06	0.91
	0.005	0.979	0.0210	0.917	0.0827	3.93	0.06	0.91
	0.007	0.976	0.0243	0.914	0.0862	3.54	0.06	0.92
DT	0	0.962	0.0382	0.793	0.2065	5.40	0.17	0.72
	0.001	0.964	0.0361	0.793	0.2061	5.71	0.17	0.72
	0.003	0.961	0.0394	0.792	0.2076	5.27	0.17	0.71
	0.005	0.961	0.0394	0.792	0.2076	5.27	0.17	0.72
	**0.007**	**0.938**	**0.0618**	**0.783**	**0.2164**	**3.50**	**0.16**	**0.70**
ANN	0	0.797	0.2027	0.623	0.3759	1.85	0.17	0.66
	0.001	0.769	0.2312	0.572	0.4269	1.85	0.20	0.67
	**0.003**	**0.856**	**0.1442**	**0.730**	**0.2693**	**1.87**	**0.13**	**0.78**
	0.005	0.839	0.1608	0.719	0.2803	1.74	0.12	0.77
	0.007	0.853	0.1473	0.702	0.2974	2.02	0.15	0.78
XGB	0	0.973	0.0268	0.915	0.0843	3.14	0.06	0.92
	**0.001**	**0.979**	**0.0207**	**0.916**	**0.0839**	**4.04**	**0.06**	**0.92**
	0.003	0.980	0.0200	0.918	0.0821	4.10	0.06	0.92
	0.005	0.980	0.0200	0.919	0.0803	4.01	0.06	0.92
	0.007	0.980	0.0201	0.913	0.0864	4.30	0.07	0.92
SVM	0	0.950	0.0504	0.847	0.1529	3.03	0.10	0.81
	0.001	0.938	0.0624	0.841	0.1580	2.53	0.10	0.83
	0.003	0.945	0.0546	0.845	0.1546	2.83	0.10	0.82
	**0.005**	**0.919**	**0.0814**	**0.840**	**0.1591**	**1.96**	**0.08**	**0.84**
	0.007	0.944	0.0556	0.845	0.1548	2.78	0.10	0.81

Supplementary Note 6. Promoter-pair response map

To retain an experimentally recognizable view of promoter effects, we back-projected the trained CatBoost model from the Magpie-based promoter-descriptor space to promoter-identity space. For each promoter identity in the curated library, the corresponding Magpie descriptor vector was assigned to the Promoter 1 or Promoter 2 slot of the canonical catalyst representation, while all remaining catalyst, synthesis, and reaction descriptors were fixed at representative baseline values. The two promoter slots were systematically enumerated across the promoter library after canonical permutation-invariant assignment, while all non-promoter variables were fixed at the same representative reference setting used for the main-text inference maps. The resulting predictions are summarized in the promoter-pair response map shown in **Fig. S23**.

Each square in **Fig. S23** corresponds to one promoter-identity pair and is partitioned into four triangular sectors reporting S_{CO} (bottom), S_{MeOH} (right), STY_{MeOH} (top, ln scale), and X_{CO2} (left), respectively. The color in each sector denotes the model-predicted response under the same background setting. The figure should therefore be interpreted as a compact multi-output screening atlas. Entries involving “Null” represent single-promoter formulations, whereas the Null–Null entry corresponds to the unpromoted baseline under the same descriptor setting, i.e., Cu/ZnO catalyst.

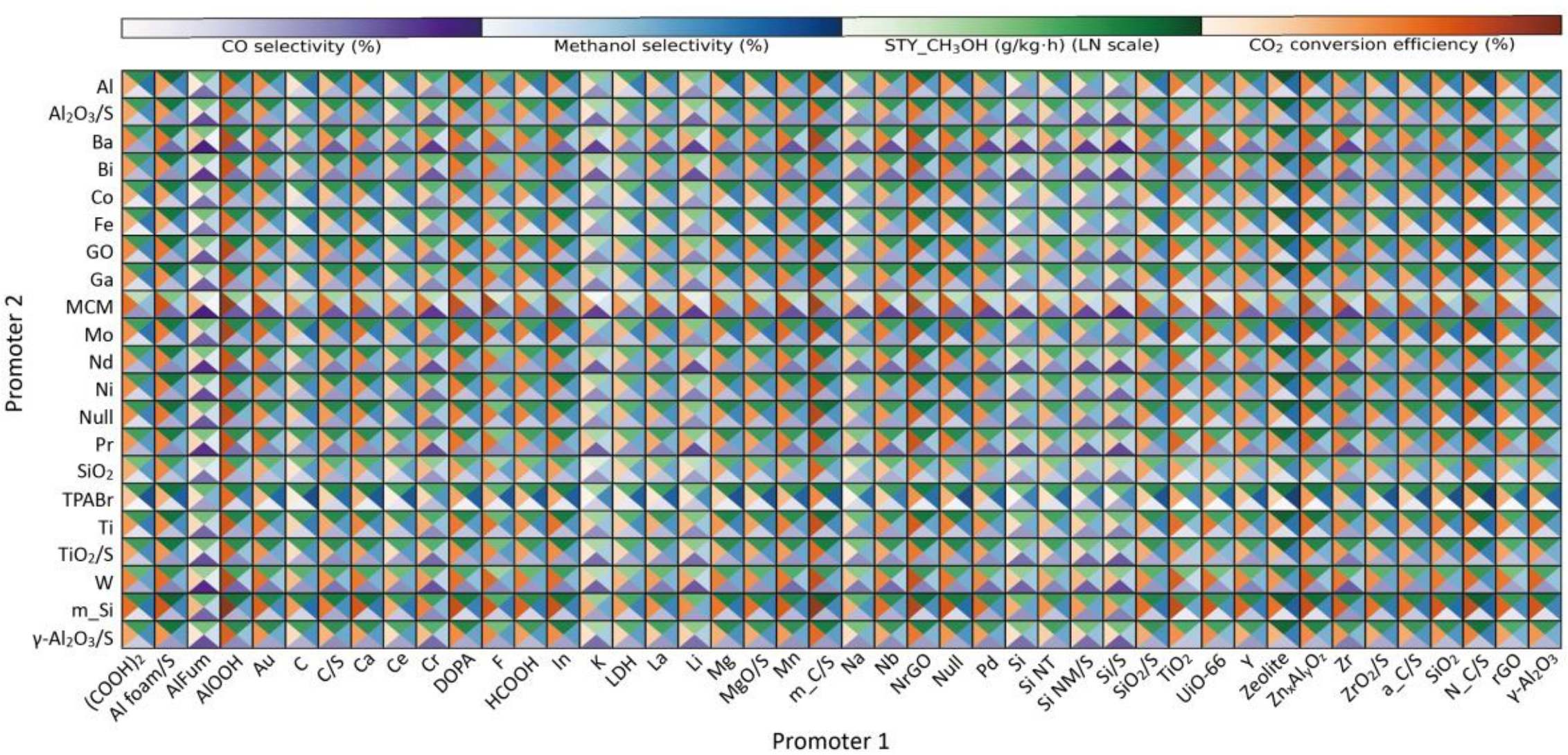

Figure S23. Promoter-pair response matrix predicted by the optimal CatBoost model under the representative reference setting used for the main-text inference analysis. Each square corresponds to one promoter-identity pair in the curated promoter library, with all remaining catalyst, synthesis, and reaction descriptors fixed at representative baseline values. The four triangular sectors report the predicted S_{CO} (bottom), S_{MeOH} (right), STY_{MeOH} (top, ln scale), and X_{CO2} (left), respectively. Promoter 1 and Promoter 2 denote the two slots in the canonical permutation-invariant representation and do not imply intrinsically distinct mechanistic roles.

Abbreviations used in the promoter labels: GO, graphene oxide; MCM, Mobil Composition of Matter (from 10.1016/j.jclepro.2016.07.099); TPABr, tetrapropylammonium bromide; m_Si, mesoporous Si; a_SiO2, amorphous SiO2; LDH, layered double hydroxide; a_C/S, activated carbon/S; m_C/S, mesocarbon/S; N_C/S, N-doped carbon/S; C/S, carbon/S; C, carbon.

Promoter 1 and Promoter 2 are slot labels inherited from the canonical permutation-invariant representation introduced during preprocessing; they do not imply intrinsically different mechanistic functions, temporal order of addition, or a strict distinction between primary and secondary promoters. Whereas the grouped SHAP analysis of **Fig. S22** identifies which materials-related descriptor groups matter most overall, **Fig. S23** shows how those effects appear when projected onto specific promoter identities.

Because the predicted influence of a promoter pair remains conditional on the fixed background composition, synthesis descriptors, and reaction conditions, **Fig. S23** should be read as a qualitative trend atlas rather than an absolute ranking of optimal catalyst formulations. Consistent with the SHAP and operating-window analyses in the main text, the map further reveals pronounced metric-dependent trade-offs: promoter pairs that favor X_{CO2} or STY_{MeOH} do not necessarily maximize S_{MeOH} or minimize S_{CO}. This observation further underscores the inherently multi-objective nature of promoter selection in Cu/ZnO-based methanol synthesis.

Supplementary Note 7. Experimental test of the ML-derived GHSV–E_{app} relationship

To directly evaluate the positive GHSV-E_{app} relationship suggested by the ML analysis, we performed a controlled fixed-bed validation experiment over a commercial $Cu/ZnO/Al_2O_3$ catalyst under low-pressure CO_2 hydrogenation conditions. This experiment was designed to assess the effect of space velocity under otherwise identical catalyst identity, feed composition, and activation conditions. The term GHSV is retained here for consistency with the literature-derived ML dataset, although the present values are reported as mass-normalized volumetric flow rates in $cm^3\ g_{cat}^{-1}\ h^{-1}$. Notably, a previous study on commercial $Cu/ZnO/Al_2O_3$ at 30 bar reported methanol-based apparent activation energies of 43–44 kJ mol^{-1} and concluded that space velocity did not influence the activation barrier under that higher-pressure operating window.[6] The present low-pressure experiment therefore probes a different kinetic regime.

Materials and fixed-bed reactor setup

A commercial copper-based methanol synthesis catalyst (denoted as CZA) was purchased from Alfa Aesar. The catalyst contains 63.5 wt% CuO, 24.7 wt% ZnO, 10.5 wt% Al_2O_3, and 1.3 wt% MgO (nominal composition from the supplier). High-purity gases were used for all experiments: CO_2, H_2, and N_2 (purity ≥ 99.99%).

Catalytic tests were conducted in a continuous-flow fixed-bed reactor operated at 1 bar. About 4.0 g of CZA catalyst was packed in the tubular reactor and fixed between quartz-wool plugs. The reactor temperature was controlled by an electric furnace, and the bed temperature was monitored by a thermocouple placed close to the catalyst bed center. Gas flow rates were regulated using mass flow controllers.

Catalyst activation and performance tests

Prior to performance measurements, the catalyst was reduced and activated in situ in a H_2 stream at a total flow of 50 $cm^3\ min^{-1}$. The temperature was raised to 350 °C and maintained for 5 h. After activation, the reactor was cooled to 160 °C and purged with N_2 for 30 min to remove residual H_2.

After stabilization, the CO_2/H_2 (1:3) feed was introduced at space velocities of 750, 1500, and 3000 $cm^3\ g_{cat}^{-1}\ h^{-1}$, respectively. Catalytic performance was evaluated from 160 to 240 °C by increasing the temperature in 10 °C increments. At each temperature setpoint, the reactor was allowed to stabilize for 20 min, after which the outlet stream was analyzed for 30 min. Each condition was measured at least three times. The reactor effluent was then analyzed on-line by gas chromatography.

Online GC analysis and calibration

The reactor outlet stream was analyzed using an online gas chromatograph (GC-2014, Shimadzu). Methanol was quantified using a flame ionization detector (FID) equipped with an HP-PLOT Q column. Permanent gases were quantified using a thermal conductivity detector (TCD) equipped with Molecular Sieve and Porapak Q columns.

Methanol formation was quantified from the GC peak area using an external calibration curve. The calibration gave a linear relationship between methanol amount and GC area with a slope of 67.266 ± 1.182, expressed as Area × 10^3 = 67.266 × CH_3OH mol × 10^8. Accordingly, the amount of methanol in each GC injection was obtained from the measured peak area. Using a 1.0 mL GC sampling loop, the methanol space-time yield was calculated as $\mathrm{STY}_{\mathrm{MeOH}} = \frac{n_{\mathrm{MeOH,loop}} \times Q \times 60 \times M_{\mathrm{MeOH}}}{V_{\mathrm{loop}} \times m_{\mathrm{cat}}}$, where $n_{\mathrm{MeOH,loop}}$ is the methanol amount per GC injection, Q is the total outlet flow rate, V_{loop} is the loop volume, and m_{cat} is the catalyst mass. The resulting $\mathrm{STY}_{\mathrm{MeOH}}$ values are reported in g $\mathrm{kg_{cat}}^{-1}$ h^{-1}.

Arrhenius fitting

Apparent activation energies for methanol formation were extracted independently at each GHSV by fitting the temperature-dependent $\mathrm{STY}_{\mathrm{MeOH}}$ to an Arrhenius-type expression, $\ln(\mathrm{STY}_{\mathrm{MeOH}}) = \ln A - E_{\mathrm{app}}/RT$, using ordinary least-squares regression of $\ln(STY_{\mathrm{MeOH}})$ against $1/T$ (T in K). To retain only the quasi-linear fitting region, data up to 210 °C were used for all flow rates, with the lower fitting boundary set to 160 °C. The apparent activation energy was calculated from the fitted slope according to $E_{\mathrm{app}} = -R \times$ slope, where $R =$ 8.314 J mol^{-1} K^{-1}. For visualization, the Arrhenius plots are shown as $\ln(STY_{\mathrm{MeOH}})$ versus $1000/T$.

Experimental outcome and mechanistic interpretation

The temperature-dependent methanol productivity and the corresponding Arrhenius plots are shown in **Fig. 7**. The three flow conditions exhibit distinct $\mathrm{STY}_{\mathrm{MeOH}}$ profiles, and the highest flow rate gives the highest methanol productivity in the intermediate-temperature window. At higher temperature, however, the $\mathrm{STY}_{\mathrm{MeOH}}$ values deviate from a single low-temperature Arrhenius trend, which motivated restriction of the fitting procedure to the quasi-linear region. Within this region, the methanol-based apparent activation energy increased monotonically with flow rate, from 57.0 ± 4.5 kJ mol^{-1} at 750 cm^3 $\mathrm{g_{cat}}^{-1}$ h^{-1} to 76.6 ± 13.2 kJ mol^{-1} at 1500 cm^3 $\mathrm{g_{cat}}^{-1}$ h^{-1} and 93.9 ± 6.8 kJ mol^{-1} at 3000 cm^3 $\mathrm{g_{cat}}^{-1}$ h^{-1}. These measurements are therefore consistent with the positive GHSV–E_{app} trend inferred from the literature-trained ML model under the present low-pressure operating window.

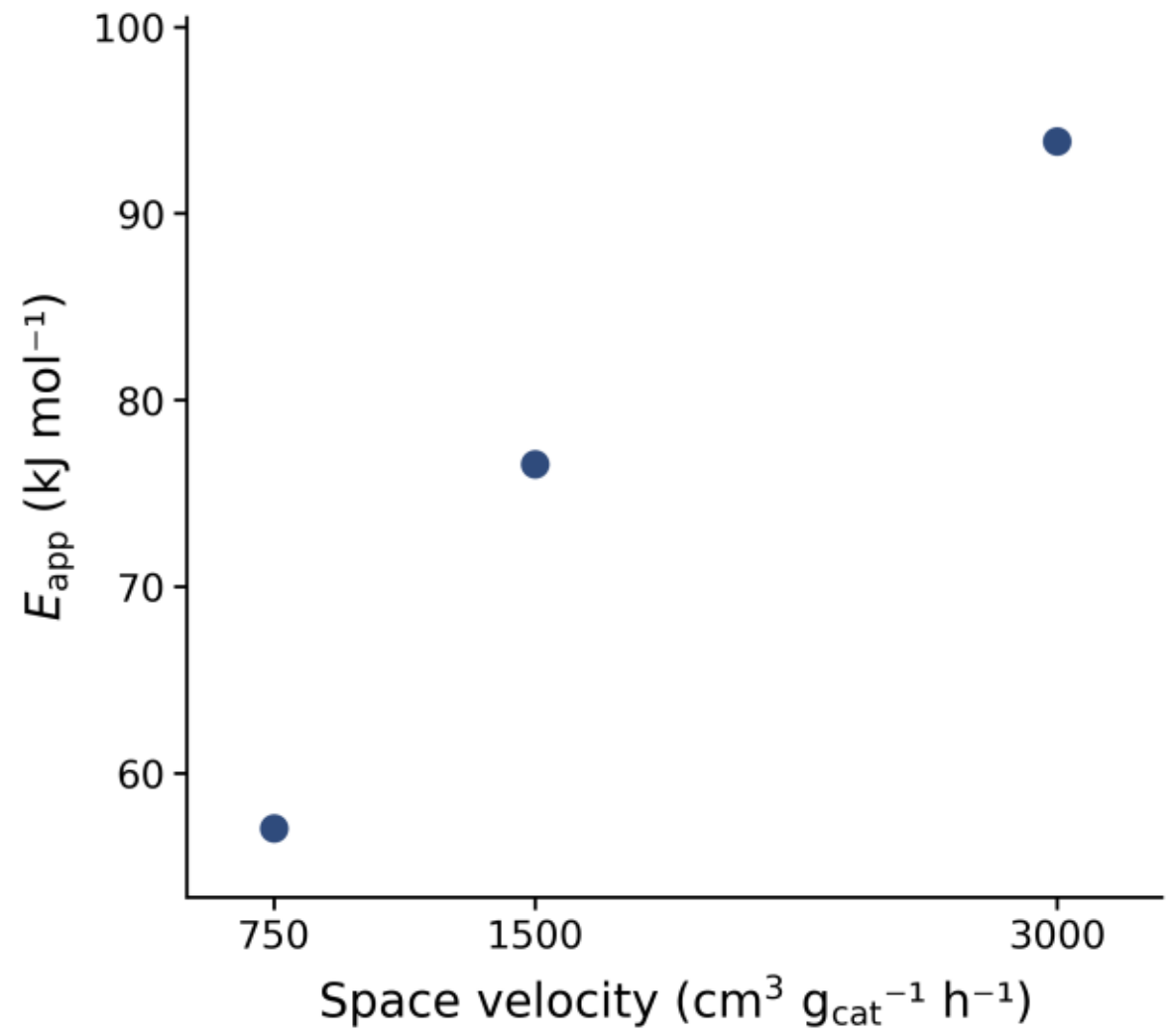

Figure S24. Experimental validation of the ML-derived GHSV–Eapp relationship. Methanol-based apparent activation energies extracted from Arrhenius fits of STY_{MeOH} increase monotonically from 57.0 ± 4.5 to 93.9 ± 6.8 kJ mol^{-1} as GHSV increases from 750 to 3000 cm^3 g_{cat}^{-1} h^{-1} over a commercial Cu/ZnO/Al_2O_3 catalyst at 1 bar. The monotonic trend supports the positive GHSV–E_{app} relationship inferred from the literature-trained model and is consistent with a flow-induced shift in the observed kinetic regime.

The mechanistic meaning of this result should be confined to observed kinetics. The E_{app} derived from reactor-level STY_{MeOH} is an operational quantity and should not be interpreted as direct evidence that flow rate changes an intrinsic elementary activation barrier. [7] For Cu-based CO_2 hydrogenation, methanol formation competes with RWGS, and the observed methanol rate can depend strongly on the operating window. Literature on Cu-based systems also suggests that H_2O may influence methanol-forming pathways under some conditions, but the underlying mechanism remains under active debate.[8] Accordingly, varying the flow rate can alter residence time and the local reaction environment along the bed, including the extent of product build-up and the relative weighting of parallel pathways, thereby changing the fitted methanol-based E_{app}. Because no operando characterization or dedicated transport analysis was performed here, the present experiment is best interpreted as demonstrating a GHSV dependence of the observed methanol-based E_{app} under this specific low-pressure condition, rather than identifying a unique mechanistic origin or a universal causal law.

Supplementary Note 8. Cross-system transferability test on electrochemical CO_2 reduction

Our literature survey spans 200 publications and the model training uses 2,383 curated, permutation-invariant experimental entries. For ML on experimental catalysis data, the key issue is not the raw number of source papers, but the number of high-quality and consistently defined data points that remain after harmonized curation. To contextualize this scale, we compiled **Table S4** and **Fig. S25** summarizing representative experimental-data ML studies across catalysis. Across reaction classes, experimental dataset sizes span from single-digit to over ten thousand data points, indicating that datasets on the order of 10^3 are not atypical once multiple sources are harmonized. The surveyed studies cover thermocatalysis (e.g., PDH, DRM, WGS, OCM, etc.), electrocatalysis (e.g., CO_2RR, NRR, OER/HER), photocatalysis, organic catalysis, and process-related datasets. CO_2 thermocatalysis (e.g., CO_2-to-methanol and CO_2-to-olefins) is listed separately. Our methanol-synthesis dataset therefore lies in the upper range of literature-curated experimental catalysis datasets and, to our knowledge, is the most extensive one in the specific CO_2-to-methanol category.

Table S4. Representative machine-learning studies based on experimental catalysis data, organized by reaction type.

**Reaction type**	**Dataset sizes (number of data points)**	**Methods**	**References (DOI)**
Dry reforming of methane (DRM)	5508;	DT; ANN	10.1016/j.ijhydene.2016.10.003;
Dry reforming of methane (DRM)	5521	DT; ANN	10.1016/j.cattod.2017.05.012
Dry reforming of methane (DRM)	27	ANN	10.1016/j.ijhydene.2016.04.034
Dry reforming of methane (DRM)	100	ANN; SVM; RT	10.1016/j.jechem.2024.04.022
CO methanation	63	K-means algorithm; Gaussian process regression (GPR)	10.1039/c7cy01634d
Oxidative coupling of methane (OCM)[a]	1870	DT	10.1002/cctc.201100186
Oxidative coupling of methane (OCM)[a]	1802;	Regression analysis	10.1038/s41467-019-08325-8;
Oxidative coupling of methane (OCM)[a]	1868	RF	10.1002/cctc.201800310
Oxidative coupling of methane (OCM)[a]	1870	Quadratic response surface model; clustered RBF neural network; RF	10.1039/c4cy01443j
Oxidative Coupling of Methane[a]	12708	Gaussian mixture model	10.1021/acs.jpclett.0c01926

CO oxidation	5610	DT; ANN; genetic algorithm-based clustering technique	10.1002/cctc.201200665
CO oxidation	1337	Neural Network	10.1021/ie2013955
CO oxidation	882	ANN	10.1016/j.apcata.2013.08.056
CO oxidation	252	DT; ANN	10.1016/j.cherd.2012.08.017
Water-gas shift reaction	4360	DT; ANN; SVM	10.1016/j.ijhydene.2014.01.160
Water-gas shift reaction	2228	ANN; PCA (Principal Component Analysis)	10.1016/j.apcatb.2019.118257
Water-gas shift reaction	344	ANN	10.1002/qsar.200430900
Supercritical water gasification	718	RF; SVM; ANN	10.1016/j.cej.2021.131285
Electrocatalytic reduction of CO_2	471	DT	10.1016/j.jcou.2018.09.011
Electrocatalytic reduction of CO_2	127	DT; RT; Neural Network	10.1021/acssuschemeng.2c02941
Electrocatalytic reduction of CO_2	5196	neural network	10.1021/acscatal.4c05530
CO_2 hydrogenation to methanol	1201	RF; GBDT; XGB	10.1016/j.apcatb.2022.121530
CO_2 hydrogenation to methanol	212	ANN	10.1016/j.fuel.2023.128045
Photocatalytic water splitting	540	Association rule mining (Apriori); DT; RF	10.1016/j.apcatb.2018.09.104
Photocatalytic valorization/reforming of glycerol to H_2	29	ANN; Genetic algorithm (GA)	10.1016/j.apcatb.2017.03.016
Electrocatalytic hydrogen evolution reaction (HER)	38	Reshef pattern recognition algorithm	10.1021/ja407394q
OER and HER	134	RT	10.1002/adma.202420465
Electrocatalytic oxygen evolution reaction (OER)	70	Multiobjective Bayesian optimization	10.1039/d3ta06651g
Electrocatalytic oxygen evolution reaction (OER)	101	Ordinary least squares; least angle regression	10.1021/acs.jpcc.5b10071
Electrocatalytic oxygen evolution reaction (OER)	2121	Gemini; Bayesian optimizer Phoenics; Bayesian neural network	http://arxiv.org/abs/2103.03391
Electrocatalytic oxygen evolution reaction (OER)	25	ANN	10.1016/j.cattod.2010.06.019
Electrocatalytic oxygen evolution reaction (OER)	2121	Sequential learning	10.1039/C9SC05999G
Selective CO_2 hydrogenation	17	ANN; RSM	10.1016/j.jcou.2017.11.013

Photocatalytic CO_2 Reduction	103	Bayesian optimization	10.1021/jacs.4c01305
Asymmetric hydrogenation of olefins	11932	GNN	10.1038/s41467-025-58854-8
Terminal olefin hydroformylation	1167	ET; NN; XGB; DT; GPR; LGBM; KNR; KRR; CBR	10.1016/j.checat.2024.101079
CO_2 to light olefins	532	DNN; SVR; RF; AdaBoost; HistG-Boost; XGBoost	10.1002/aic.18437
Photocatalytic glycerol valorization to hydrogen	29	Response surface methodology; ANN	10.1016/j.apcatb.2017.03.016
Reverse osmosis desalination	838	DT	10.1016/j.jclepro.2025.146979
lipid production from Yarrowia lipolytica	356	DT	10.1016/j.fuel.2021.122817
tri-reforming of biogas	1183	RF; DT	10.1016/j.ijhydene.2025.04.213
Electrochemical nitrogen reduction reaction (NRR)	520	Linear regression, decision tree, random forest, and multilayer perceptron	10.1039/D3DD00151B
Propane Dehydrogenation	10155	Decision Tree (DTR), Random Forest (RFR), CatBoost, Extra Tree (ETR), XGBoost, Gradient Boosting (GBR), LightGBM, Hist Gradient Boosting (HGB), Gaussian Process (GPR), Support Vector (SVR) regression algorithms, Artificial Neural Networks (ANN), Deep Neural Networks (DNN)	10.1021/acscatal.5c06285

[a] Data collected through high-throughput experiments. Nguyen, T. N.; Nhat, T. T. P.; Takimoto, K.; Thakur, A.; Nishimura, S.; Ohyama, J.; Miyazato, I.; Takahashi, L.; Fujima, J.; Takahashi, K.; Taniike, T. High-Throughput Experimentation and Catalyst Informatics for Oxidative Coupling of Methane. *ACS Catal.* 2020, 10, 921−932.

[b] A significant break-through in terms of the dataset quality was reported by Zavyalova et al. in 2011.

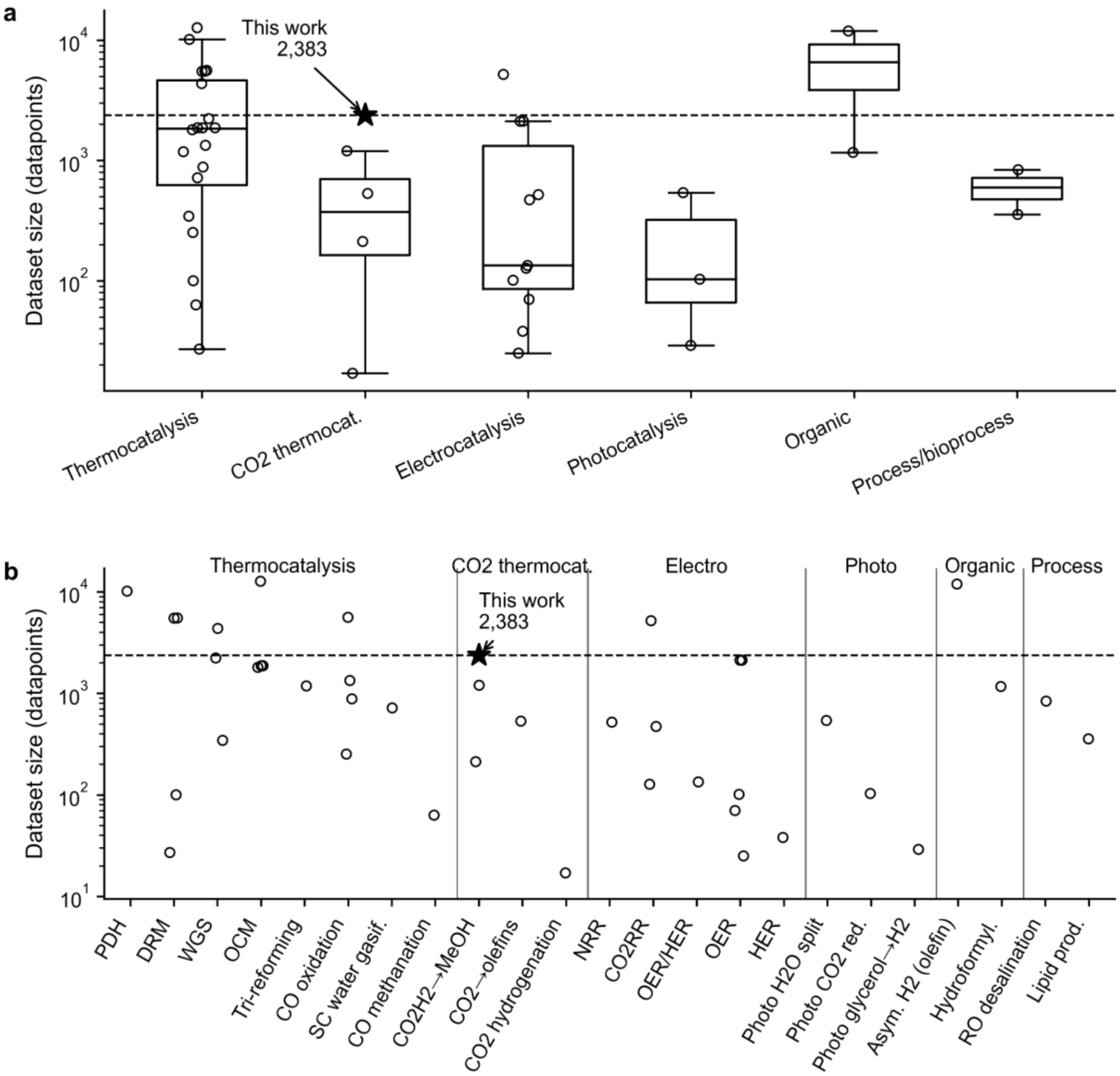

Figure S25. **Dataset sizes reported in representative experimental-data machine-learning studies of catalysis.** Boxplots in panel (a) summarize the distribution of curated dataset sizes across reaction domains, and individual study values are shown in panel (b). The star and dashed line denote the present work (2,383 permutation-invariant entries).

Abbreviations: PDH, propane dehydrogenation; DRM, dry reforming of methane; WGS, water–gas shift; OCM, oxidative coupling of methane; Tri-reforming, tri-reforming of biogas; SC water gasif., supercritical water gasification; CO_2H_2→MeOH, CO_2 hydrogenation to methanol; CO_2→olefins, CO_2 to light olefins; NRR, nitrogen reduction reaction; CO_2RR, CO_2 reduction reaction; OER, oxygen evolution reaction; HER, hydrogen evolution reaction; Photo H_2O split, photocatalytic water splitting; Photo CO_2 red., photocatalytic CO_2 reduction; Photo glycerol→H_2, photocatalytic reforming of glycerol to H_2; Asym. H_2 (olefin), asymmetric hydrogenation of olefins; Hydroformyl., terminal olefin hydroformylation; RO desalination, reverse osmosis desalination; Lipid prod., lipid production from *Yarrowia lipolytica*.

Moreover, one additional catalytic system is chosen to demonstrate that the ML framework remains viable in substantially smaller-data regimes. We selected Cu-based electrochemical CO_2 reduction (CO_2RR) as an auxiliary transferability test because it differs fundamentally from the main thermocatalytic methanol-synthesis case in reaction environment, driving force, descriptor space, and product manifold, while remaining a catalytically complex CO_2-conversion chemistry amenable to literature curation.

Building on our previous study,[9] we assembled a curated CO_2RR dataset comprising 379 literature-derived entries. Each entry corresponds to a unique catalyst–electrolyte–operation point. The input descriptors comprised Cu-based catalyst phase, Cu-based catalyst shape, electrode support, particle size, applied potential versus RHE, electrolyte cation identity and concentration, electrolyte anion identity and concentration, membrane type, and, where available, current density. The outputs were grouped product fractions of H2, CO, C1, and C2+. All potentials were converted to V versus RHE, particle sizes to nm, and electrolyte concentrations to M. Categorical variables were encoded analogously to the main thermocatalytic workflow, and numerical variables were transformed and standardized where appropriate. Descriptor distributions after preprocessing are shown in **Fig. S26**.

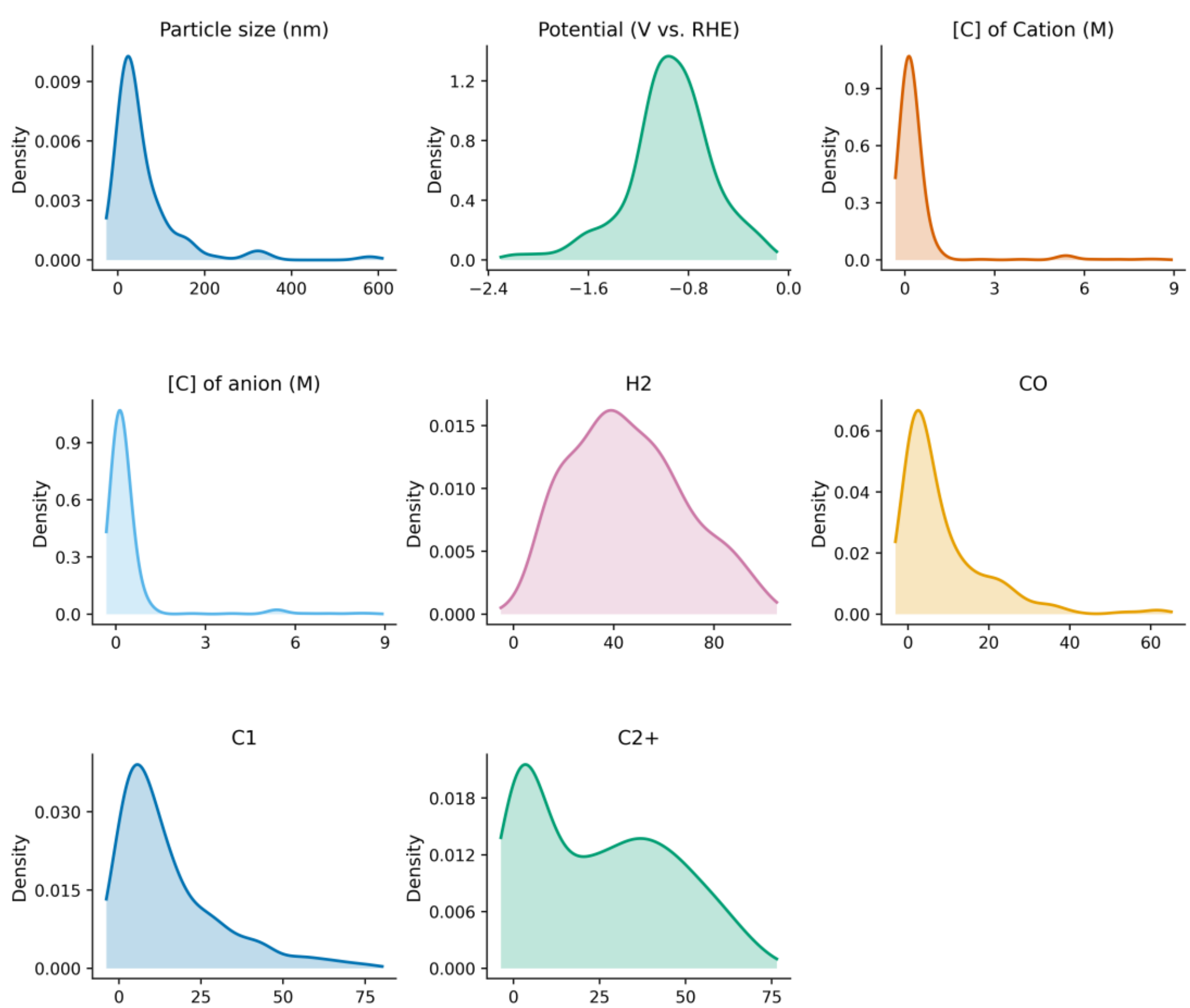

Figure S26. Distributions of selected numerical features after preprocessing.

We then applied the same strategy as in the main thermocatalytic dataset, evaluating six ML models under an identical tuning and validation protocol. CatBoost delivered the strongest overall predictive performance. Although the unregularized CatBoost model gave the highest aggregate CV R^2, it also displayed pronounced overfitting. We therefore retained the CatBoost model with α = 0.3 as the best candidate. This model achieved validation R^2 values of 0.625 for H_2, 0.714 for CO, 0.755 for C1, and 0.708 for C2+, with an aggregate CV R^2 of 0.736 (**Table S5**). These results show that the framework retains predictive usefulness even in this smaller-data electrocatalytic setting.

Table S5. Historical comparison of per-output validation R^2, aggregate CV R^2, and overfitting metrics for six ML models at different regularization strengths (α)

Model	α	R^2 (Valid)				Overfitting		CV (R^2)
		H_2	CO	C1	C2+	MSE Ratio	R^2 difference	
RF	0.0	0.520	0.637	0.643	0.677	5.57	0.31	0.691
	0.03	0.353	0.484	0.446	0.469	1.53	0.21	0.490
	**0.3**	**0.515**	**0.618**	**0.625**	**0.670**	**4.20**	**0.30**	**0.679**
	0.5	0.507	0.614	0.605	0.658	3.48	0.29	0.670
	0.7	0.515	0.629	0.633	0.674	4.58	0.31	0.680
CatBoost	0.0	0.661	0.747	0.794	0.717	47.55	0.26	0.759
	0.03	0.635	0.735	0.761	0.709	12.41	0.27	0.746
	**0.3**	**0.625**	**0.714**	**0.755**	**0.708**	**7.14**	**0.26**	**0.736**
	0.5	0.532	0.610	0.682	0.653	2.82	0.25	0.678
	0.7	0.483	0.566	0.671	0.616	2.53	0.26	0.657
DT	0.0	0.414	0.244	0.541	0.374	3.02	0.41	0.504
	**0.03**	**0.411**	**0.229**	**0.554**	**0.253**	**6.21**	**0.54**	**0.528**
	0.3	0.405	0.266	0.387	0.371	1.14	0.10	0.312
	0.5	0.405	0.266	0.387	0.371	1.14	0.10	0.312
	0.7	0.342	0.195	0.451	0.405	1.27	0.15	0.304
ANN	**0.0**	**0.379**	**0.276**	**0.539**	**0.434**	**1.61**	**0.24**	**0.591**
	0.03	-0.035	0.058	-0.012	-0.114	1.07	0.10	0.306
	0.3	0.298	0.203	0.517	0.416	1.51	0.23	0.575
	0.5	0.286	0.254	0.545	0.396	1.55	0.24	0.574
	0.7	0.288	0.261	0.482	0.438	1.39	0.19	0.617
XGB	0.0	0.591	0.503	0.723	0.719	55.50	0.36	0.713

	0.03	0.544	0.452	0.696	0.709	22.35	0.38	0.682
	0.3	0.575	0.494	0.740	0.725	52.89	0.36	0.711
	**0.5**	**0.546**	**0.523**	**0.700**	**0.712**	**10.81**	**0.35**	**0.693**
	0.7	0.557	0.489	0.745	0.685	22.20	0.36	0.707
SVM	0.0	0.573	0.316	0.706	0.575	4.23	0.35	0.611
	0.03	0.446	0.255	0.730	0.498	1.80	0.24	0.553
	0.3	0.501	0.416	0.689	0.572	2.90	0.30	0.597
	0.5	0.467	0.433	0.716	0.564	2.51	0.28	0.604
	**0.7**	**0.513**	**0.407**	**0.715**	**0.595**	**2.72**	**0.28**	**0.609**

Interpretation of the selected model further supports its chemical plausibility. SHAP analysis identified applied potential as the dominant descriptor across all four outputs. Particle size emerged as the leading continuously varying structural descriptor, whereas the relative contributions of active phase, electrode support, membrane type, and shape depended on the product group. By contrast, electrolyte identity and concentration exerted secondary influence within the sampled domain (**Fig. S27a–d**). This ranking is physically sensible for CO_2RR, where interfacial driving force, catalyst identity, and local transport environment govern the competition between HER and carbon-containing products.

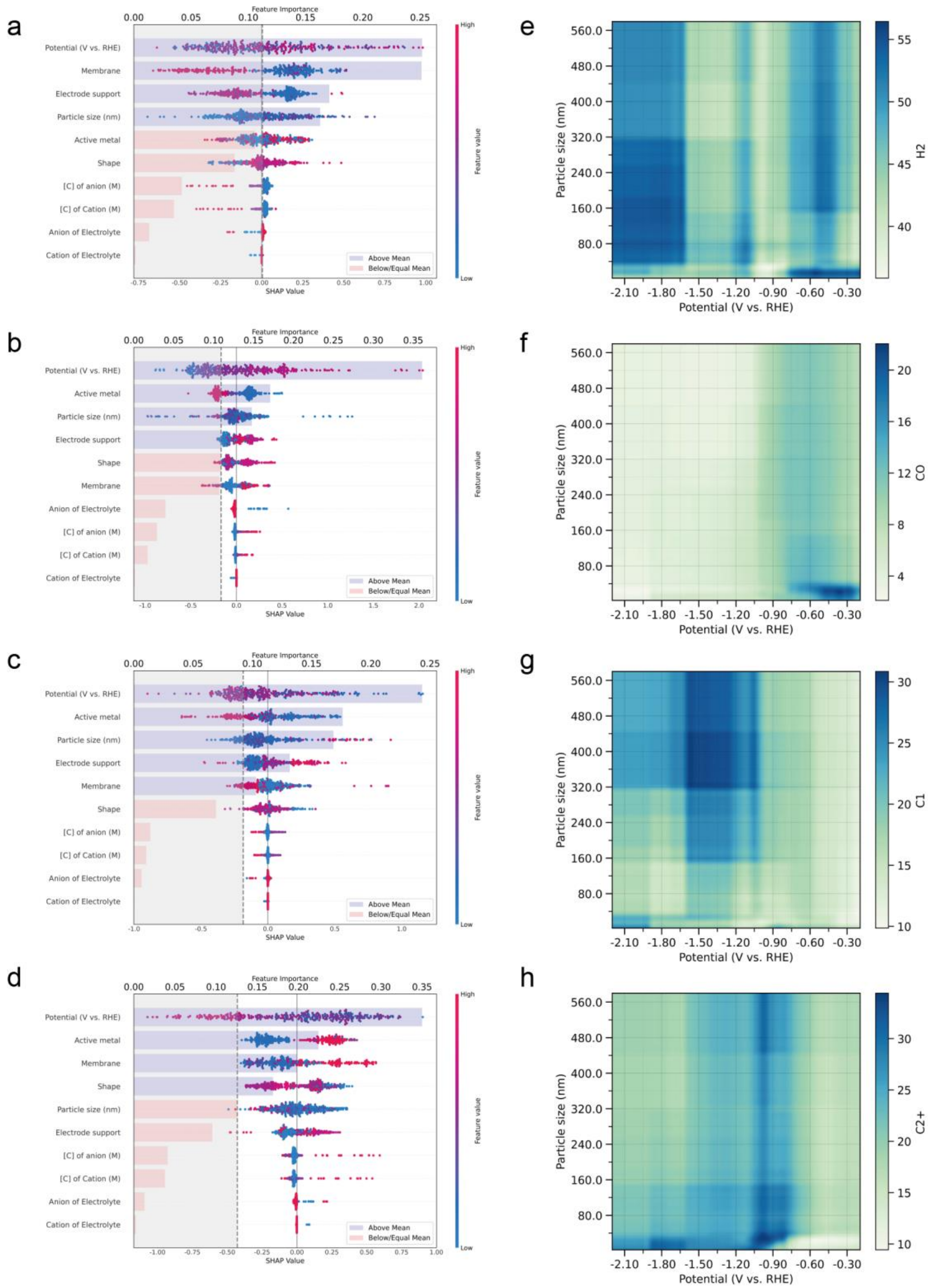

Figure S27. SHAP summaries (a–d) and potential–particle size response maps (e–h) for H_2, CO, C1, and C2+ predicted by the selected CatBoost model (α = 0.3).

To translate descriptor rankings into response patterns, we generated pairwise inference maps using the two leading continuously varying variables, applied potential and particle size. The resulting landscapes were output-specific and clearly non-monotonic (**Fig. S27e–h**). H_2 was favored at more cathodic potentials across broad size windows, whereas CO increased toward less negative potentials and smaller particles. C1 reached higher predicted fractions in an intermediate potential regime and over mid-to-large particle sizes, while C2+ occupied a narrower region jointly determined by potential and particle size. These patterns indicate that the framework captures descriptor interactions rather than simple one-variable trends.

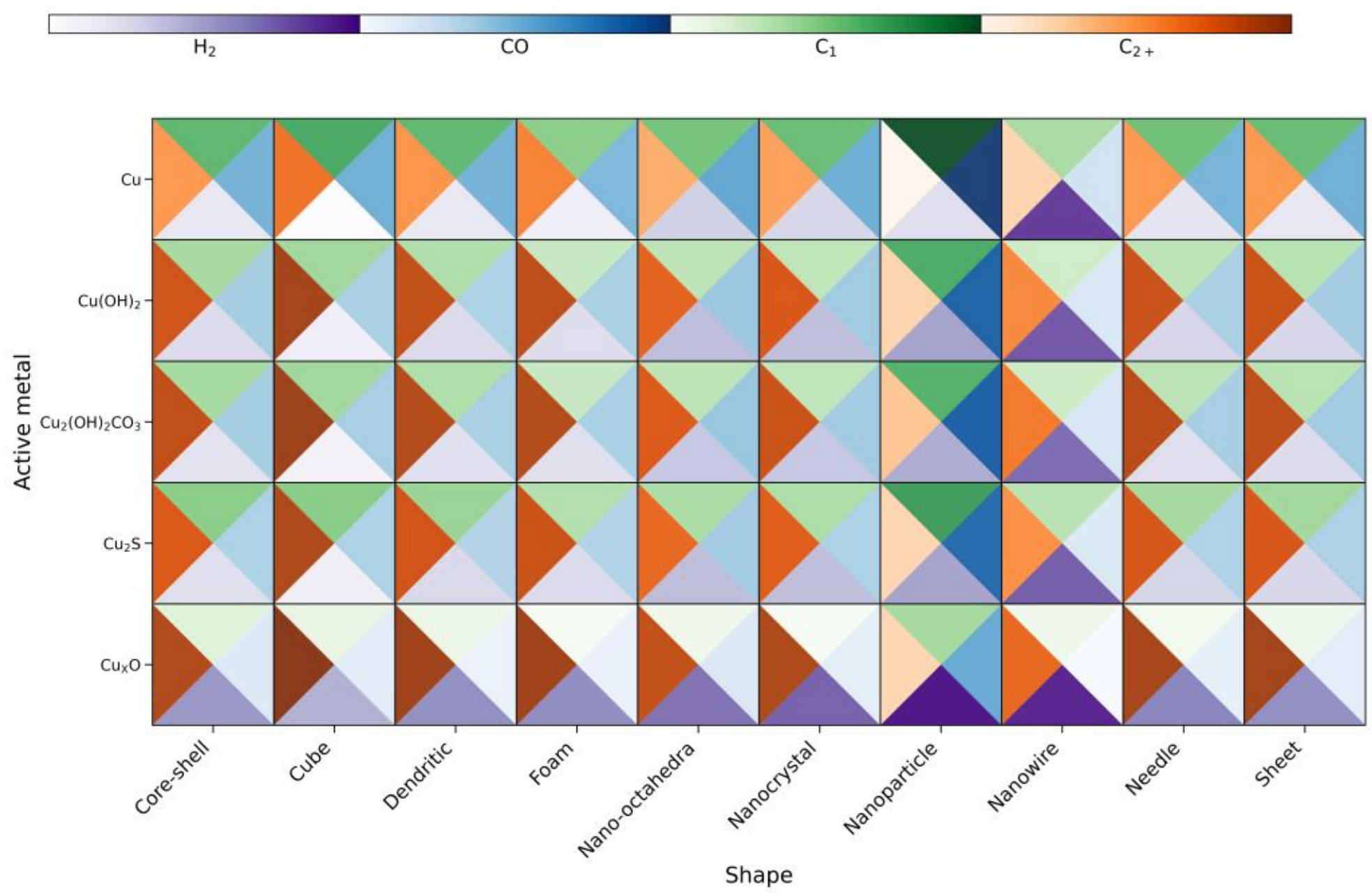

Figure S28. Active phase–shape map of predicted H2, CO, C1, and C2+ fractions from the selected CatBoost model (α = 0.3).

A complementary categorical inference map across active phase and catalyst shape further showed that product distributions are jointly reorganized by phase identity and morphology (**Fig. S28**). Rather than pointing to a single universally optimal phase–shape pair, these predictions emphasize that different combinations redistribute the balance among H_2, CO, C1, and C2+ channels in distinct ways, consistent with the strong morphology- and phase-sensitivity of CO_2RR.

This auxiliary case study is not intended to establish a universal predictive model for all CO_2RR chemistries, nor to claim full automation of literature extraction. Rather, it serves as a transferability test of the workflow itself. The recovery of chemically meaningful electrocatalytic drivers and interpretable multidimensional response surfaces across a reaction regime fundamentally different from thermocatalytic methanol synthesis supports the broader applicability of the present literature-driven interpretable machine-learning framework when sufficiently structured data can be curated.

Supplementary References

1. Bukhtiyarova, M., Lunkenbein, T., Kähler, K. & Schlögl, R. Methanol Synthesis from Industrial CO_2 Sources: A Contribution to Chemical Energy Conversion. *Catal. Letters* **147**, 416–427 (2017).
2. Murzin, D. Y. On Apparent Activation Energy of Structure Sensitive Heterogeneous Catalytic Reactions. *Catal. Letters* **149**, 1455–1463 (2019).
3. Wang, Y. *et al.* Strong Evidence of the Role of H_2O in Affecting Methanol Selectivity from CO_2 Hydrogenation over Cu-ZnO-ZrO_2. *Chem* **6**, 419–430 (2020).
4. Yao, C. *et al.* Exploring the Influence of Applied Potential and Catalyst Morphology on the Selectivities of Copper Toward Carbon Dioxide Electroreduction Using Machine Learning. *Adv. Intell. Syst.* **7**, (2025).
5. Bhardwaj, A., Ahluwalia, A. S., Pant, K. K. & Upadhyayula, S. A principal component analysis assisted machine learning modeling and validation of methanol formation over Cu-based catalysts in direct CO_2 hydrogenation. *Sep. Purif. Technol.* **324**, 124576 (2023).
6. Suvarna, M., Araújo, T. P. & Pérez-Ramírez, J. A generalized machine learning framework to predict the space-time yield of methanol from thermocatalytic CO_2 hydrogenation. *Appl. Catal. B* **315**, (2022).
7. Vanjari, P., Kamesh, R. & Rani, K. Y. Machine learning models representing catalytic activity for direct catalytic CO_2 hydrogenation to methanol. *Mater. Today Proc.* **72**, 524–532 (2023).
8. Ghafarian Nia, S. A. *et al.* Machine learning-driven optimization for sustainable CO_2-to-methanol conversion through catalytic hydrogenation. *Energy Convers. Manag.* **325**, 119373 (2025).
9. Roy, D., Mandal, S. C. & Pathak, B. Machine Learning Assisted Exploration of High Entropy Alloy-Based Catalysts for Selective CO_2 Reduction to Methanol. *J. Phys. Chem. Lett.* **13**, 5991–6002 (2022).
